# Supplementary material for: The CCCH-type zinc-finger PfMD3 promotes translation for malaria parasite transmission
Source: Nat Commun. 2026 Nov 3;17:11370. doi: 10.1038/s41467-026-77156-1 (PMC13731652; doi:10.1038/s41467-026-77156-1)
Supplement: Supplementary file 1 [file 41467_2026_77156_MOESM10_ESM.pdf]

nature portfolio

Corresponding author(s): Dr. Heather Painter
Last updated by author(s): Jul 24, 2026

Reporting Summary

Nature Portfolio wishes to improve the reproducibility of the work that we publish. This form provides structure for consistency and transparency in reporting. For further information on Nature Portfolio policies, see our Editorial Policies and the Editorial Policy Checklist.

Statistics

For all statistical analyses, confirm that the following items are present in the figure legend, table legend, main text, or Methods section.

n/a	Confirmed	
☐	☒	The exact sample size (*n*) for each experimental group/condition, given as a discrete number and unit of measurement
☐	☒	A statement on whether measurements were taken from distinct samples or whether the same sample was measured repeatedly
☐	☒	The statistical test(s) used AND whether they are one- or two-sided<br>*Only common tests should be described solely by name; describe more complex techniques in the Methods section.*
☐	☒	A description of all covariates tested
☒	☐	A description of any assumptions or corrections, such as tests of normality and adjustment for multiple comparisons
☐	☒	A full description of the statistical parameters including central tendency (e.g. means) or other basic estimates (e.g. regression coefficient) AND variation (e.g. standard deviation) or associated estimates of uncertainty (e.g. confidence intervals)
☐	☒	For null hypothesis testing, the test statistic (e.g. *F*, *t*, *r*) with confidence intervals, effect sizes, degrees of freedom and *P* value noted<br>*Give P values as exact values whenever suitable.*
☒	☐	For Bayesian analysis, information on the choice of priors and Markov chain Monte Carlo settings
☒	☐	For hierarchical and complex designs, identification of the appropriate level for tests and full reporting of outcomes
☒	☐	Estimates of effect sizes (e.g. Cohen's *d*, Pearson's *r*), indicating how they were calculated

Our web collection on statistics for biologists contains articles on many of the points above.

Software and code

Policy information about availability of computer code

Data collection	No code used for data collection.
Data analysis	Open source software for nucleotide sequence analysis: Galaxy v24.1.3 including tools:Kallisto v0.48, Freebayes (v1.3.8), SNPeff (version 5.2), BWA-MEM2 v2.2.1, FastQC v 0.74, Galaxy CLIP-explorer for access to PureCLIP and Piranha tools (https://clipseq.usegalaxy.eu/.)<br>For scRNAseq: Cell Ranger v9.0.1, Seurat (v5.2.0), DoubletFinder (v2.0.4), SCTransform (v0.4.2), Harmony v1.2.3, Slingshot (v2.14.0).<br>For GO analysis and basic data analysis and visualization: ShinyGO 0.80., In Rstudio v4.2.3, packages Pheatmap v1.0.12 and ggplot2 v3.5.1.<br>For Mass Spectrometry processing and analysis: EncyclopeDIA version 1.12.31 using Open JDK 13 on a Slurm 19.05.5 cluster running under Ubuntu 20.04, msConvert version 3.0.19106 through Proteowizard, Prosit, DIA-NN 1.8.1<br>For analysis of RNA-bind-n-seq:RBNS pipeline (https://github.com/cburgelab/RBNS_pipeline)<br>Open source algorithms for motif searches, STREME (https://meme-suite.org/meme/doc/streme.html) and domain identification: NCBI conserved domain search (https://www.ncbi.nlm.nih.gov/Structure/cdd/wrpsb.cgi), Interproscan (https://www.ebi.ac.uk/interpro/search/sequence/), NoD (Nucleolar localization sequence Detector), NLStradamus.<br>Open source software for sequence alignment and phylogenetic tree construction: MUSCLE (https://www.ebi.ac.uk/Tools/msa/muscle/), (iTOL) https://itol.embl.de/tree.<br>Database used for accessing Plasmodium data: www.Plasmodb.org<br>Open source software used for Plasmid vector design: Benchling (https://www.benchling.com) and Casoffinder

For manuscripts utilizing custom algorithms or software that are central to the research but not yet described in published literature, software must be made available to editors and reviewers. We strongly encourage code deposition in a community repository (e.g. GitHub). See the Nature Portfolio guidelines for submitting code & software for further information.

Data

Policy information about availability of data

All manuscripts must include a data availability statement. This statement should provide the following information, where applicable:

- Accession codes, unique identifiers, or web links for publicly available datasets
- A description of any restrictions on data availability
- For clinical datasets or third party data, please ensure that the statement adheres to our policy

For genomic sequencing and scRNAseq results, raw reads were submitted to SRA:PRJNA1111533. For experiments that generated RNA, files were submitted to GEO: RNA-seq experiments, raw fastq.gz and processed Kallisto quantification files, accession number: GSE267361 and for CLIP-seq experiment raw fastq.gz and processed bigwig files, accession number: GSE267362. Data from proteomics and IP-MS experiments were submitted to the MassIVE repository (https://massive.ucsd.edu/) with the identifier MSV000094788 and ProteomeXchange (http://www.proteomexchange.org) with the identifier PXD052415. Original Western blot image for Figure 1D is included in Supplementary information and microscopy data were submitted to the BioImage Archive with accession number: S-BIAD3759 (https://www.ebi.ac.uk/biostudies/bioimages/studies/S-BIAD3759). All raw data included in Source Data.

Research involving human participants, their data, or biological material

Policy information about studies with human participants or human data. See also policy information about sex, gender (identity/presentation), and sexual orientation and race, ethnicity and racism.

Reporting on sex and gender	n/a
Reporting on race, ethnicity, or other socially relevant groupings	n/a
Population characteristics	n/a
Recruitment	n/a
Ethics oversight	n/a

Note that full information on the approval of the study protocol must also be provided in the manuscript.

Field-specific reporting

Please select the one below that is the best fit for your research. If you are not sure, read the appropriate sections before making your selection.

[x] Life sciences [ ] Behavioural & social sciences [ ] Ecological, evolutionary & environmental sciences

For a reference copy of the document with all sections, see nature.com/documents/nr-reporting-summary-flat.pdf

Life sciences study design

All studies must disclose on these points even when the disclosure is negative.

Sample size	For phenotypic experiments, n=3 biological replicates were chosen as starting point and viewed as sufficient based on low variability between experimental outcomes. For mosquito transmission and -Omics experiments, n=2 biological replicates were chosen as starting point and viewed as sufficient based on low variability between experimental outcomes. Had there been enough variability in these experiments to create significant uncertainty in the experimental outcomes, additional replicates would have been performed.
Data exclusions	No data excluded
Replication	All attempts at replication were successful
Randomization	For most of the study, randomization was not applicable. Many of the experiments were conducted with in vitro cultured parasites, in which entire populations of the parasites were included for each biological replicate. For mosquito transmission experiments, batches of parasitized blood containing either the knockout or wild-type parasite strains were fed to equal numbers of female Anoheles stephensi mosquitoes and mosquitoes selected for dissection at random.
Blinding	For experiments involving microscopy comparing the knockout and wild-type parasite strains, sample slides were de-identified prior to counting, such that microscopists were blind to which groups or timepoints slides were representative of.

Reporting for specific materials, systems and methods

We require information from authors about some types of materials, experimental systems and methods used in many studies. Here, indicate whether each material, system or method listed is relevant to your study. If you are not sure if a list item applies to your research, read the appropriate section before selecting a response.

Materials & experimental systems

n/a	Involved in the study
	[x] Antibodies
	[x] Eukaryotic cell lines
[x]	Palaeontology and archaeology
[x]	Animals and other organisms
[x]	Clinical data
[x]	Dual use research of concern
[x]	Plants

Methods

n/a	Involved in the study
[x]	ChIP-seq
	[x] Flow cytometry
[x]	MRI-based neuroimaging

Antibodies

Antibodies used	Antibodies from Santa Cruz Biotechnology: rat anti-HA (Santa Cruz, sc-53516), mouse anti-HA (Santa Cruz, sc-7392)<br>Antibodies from Abcam: anti-Pfaldolase (ab207494), anti-histone H3 (ab18521), anti-rat HRP (ab7097), anti-rabbit HRP (ab6721)<br>Additional suppliers: α-HA antibody (Roche, 3F10) or 20 μg IgG antibody (Millipore Sigma, PP68) rat Alexa-488 (1:333, Invitrogen, ab150157) or mouse Alexa-568 (1:333, Invitrogen, A10037)<br>Antibody gifted by researcher: anti-P230p, anti-P47
Validation	Anti-P230p antibody validated for use in identifying male Plasmodium falciparum gametocytes in doi:10.1038/s41598-018-33236-x (2018).<br>Antibodies from Santa Cruz Biotechnology validated for use in Western Blot and immunoprecipitation experiments (https://www.scbt.com/p/ha-probe-antibody-153, https://www.scbt.com/p/ha-probe-antibody-f-7<br>Antibodies from Abcam validated for use in Western Blot experiments: https://www.abcam.com/en-us/products/primary-antibodies/histone-h3-antibody-ab18521, https://www.abcam.com/en-us/products/primary-antibodies/plasmodium-aldolase-antibody-ab207494<br>Roche 3F10 antibody used for immunoprecipitation of 3xHA tagged Plasmodium falciparum protein: https://www.nature.com/articles/s41467-020-15026-0#Sec9<br>Millipore sigma rat IgG antibody control: https://www.sigmaaldrich.com/US/en/product/mm/pp68?srsltid=AfmBOoqADd3Ca9i8Aye3aZPdJsQ1E5WjnUNjFTqH7oOXwc7HNth_xEAk

Eukaryotic cell lines

Policy information about cell lines and Sex and Gender in Research

Cell line source(s)	Plasmodium falciparum strain NF54e parent was obtained from BEI Resources (MRA-1000) and the strain was validated by whole genome sequencing by the vendor.
Authentication	Cell lines generated from NF54e parent strain were authenticated via whole genome sequencing and compared to both the NF54e parent genome as well as the isogenic 3D7 reference strain. These results can be found in Table S6.
Mycoplasma contamination	All cell lines tested negative for mycoplasma contamination by e-Myco PCR Mycoplasma Detection Kit
Commonly misidentified lines<br>(See ICLAC register)	Not applicable

Plants

Seed stocks	n/a
Novel plant genotypes	n/a
Authentication	n/a

Flow Cytometry

Plots

Confirm that:

- [ ] The axis labels state the marker and fluorochrome used (e.g. CD4-FITC).
- [ ] The axis scales are clearly visible. Include numbers along axes only for bottom left plot of group (a 'group' is an analysis of identical markers).
- [ ] All plots are contour plots with outliers or pseudocolor plots.
- [x] A numerical value for number of cells or percentage (with statistics) is provided.

Methodology

Sample preparation	Following culture in vitro, samples were collected for flow cytometric analysis at 0-, 10- and 15-min post-activation of exflagellation, which involves nuclear division, to measure the nuclear content over time. Parasites were fixed with 4% formaldehyde, 0.0075% glutaraldehyde in 1x phosphate buffered saline (PBS) and stored at 4°C until measurement. For flow cytometry, fixative was removed by washing three times with PBS before incubation with 1x SYBR Green I (Fisher Scientific, BMA50513) in PBS for 1 h while shaking. Following staining, parasites were washed three times with PBS and nuclear content determined on an Attune Flow Cytometer, using SYBR Green I-stained uninfected erythrocytes as background control for gating.
Instrument	Attune Flow NxT Cytometer
Software	Attune Flow NxT Cytometer software
Cell population abundance	This experiment was only used to enumerate cells with increased nuclear content, cells were not sorted for this experiment.
Gating strategy	An initial gate was constructed from uninfected erythrocytes based on FSC and SSC values to exclude lysed cell material and doublets. A secondary gate for excluding erythrocytes was created to enumerate cells emitting FITC-channel fluorescence higher than technical triplicates of the stained uninfected erythrocyte control population. Finally the timepoint prior to activation (0 min) was used to construct a gate denoting the parasite population prior to DNA synthesis and cell division of exflagellating parasites, as has been published: 10.1016/S0092-8674(04)00449-0

- [ ] Tick this box to confirm that a figure exemplifying the gating strategy is provided in the Supplementary Information.
