# Supplementary material for: The CCCH-type zinc-finger PfMD3 promotes translation for malaria parasite transmission
Source: Nat Commun. 2026 Nov 3;17:11370. doi: 10.1038/s41467-026-77156-1 (PMC13731652; doi:10.1038/s41467-026-77156-1)
Supplement: Supplementary file 2 [file 41467_2026_77156_MOESM11_ESM.pdf]

nature portfolio

Peer Review File

The CCCH-type zinc-finger *Pf*MD3 promotes translation for malaria parasite transmission

Corresponding Author: Dr Heather Painter

This file contains all reviewer reports in order by version, followed by all author rebuttals in order by version.

Version 1:

Reviewer comments:

Reviewer #1

(Remarks to the Author)
The CCCH-type zinc-finger PfMD3 regulates translation needed for malaria parasite transmission by van Biljon et al presents an in-depth characterisation of PfMD3 across the P. falciparum asexual and gametocyte stages. The authors multi-omics approach has identified PfMD3 as 'the first male-associated regulator of translation during gametocytogenesis'. This work builds on the finding of Farrukh et al. (Ref 63) who previously undertook a similar phenotypic analysis of PfMD3. Biljon et al. characterisation of PfMD3 KOs impact on transmission, identification of the RNA binding motif and characterisation of this proteins role in facilitating translation of male gametocyte specific genes builds significantly beyond our current understanding of this proteins function.

This work provides important insight into malaria parasite gene and protein regulation for which there are still many unknowns and identifies potential avenues for future drug and vaccine exploration. This paper is generally well-written and enjoyable to read. Reasoning and conclusions are generally well-supported. Some experimental outcomes need to be clarified and the text around the outcomes of some of the 'omics' experiments could be improved as the message is being lost.

Major comments

Lines 144-146, Figure 1D, S1C and S1D: The authors state that PfMD3 expression peaks in early gametocytes (stages II – IV) however there is only samples for stages II and V on this western blot image. Suggest re-wording to exclude mention of stage 'IV'. Or change 'IV' to 'V' as both stages look to have a very similar level of expression (based on S1D also).

Data and interpretation could be strengthened here by completing additional replicates for the time course western blots (n=3), as it appears there is only one replicate represented here. There is also minimal banding evident for the ring, troph, schizont fractions, yet the conclusion is that the protein is expressed across the lifecycle. While it is true that the protein is more obvious on the IFAs and in the western blot of Farrukh et al. (Ref 63), the text implies (Line 143 and Line 151) that Fig 1D and S1D demonstrate expression in asexual blood stages, and it is not at all clear that it is meaningfully expressed in these stages from this data.

The distinction between nuclear and cytoplasmic extracts is unclear in S1C. It would help to describe what is being addressed here, it is not even mentioned in the Figure Legend. What stages are represented in the fractionation blots? The PfMD3 bands are barely visible in these blots, and the aldolase bands are visible in both extracts, so it would be better to repeat or remove than leave as is.

Figure 2B, C, D: The idea that the increased asexual parasite growth seen for PfMD3 KO parasites is a result in reduced commitment to gametocytemia (2C, 2D) is nice. Some things to consider.
1) If the assay conditions between 2B and 2C are equivalent, then it should be possible to mathematically assess whether the lost gametocyte production in 2C is equivalent to the increased asexual parasite growth seen in 2B. Would you actually expect a 50% reduction in sexual stage commitment to result in a near 20% increase in asexual parasite growth? That would imply 30-40% of your asexual stage parasites are converting every cycle I think, which seem high for general in vitro parasite culture.
2) If the assays are not equivalent (e.g. 2B is a standard growth assay whereas 2C is a gametocyte production that relies on

stressing the parasites to induce higher rates of gametocyte commitment) then it would difficult to assess such proportionality between the two assays. But likewise, the pressures leading to gametocytogenesis differ between the assays.
Other possible reasons for increased growth are not covered, such as a change in nutrient acquisition, protein expression, or cloning artefacts. So while the idea is nice, the argument would be strengthened if proportionality could be indicated and perhaps some other possible reasons explored or discounted around how the assays are done.

Line 345 onwards: The text through this section is difficult to follow and trying to keep track of what protein sets are being discussed is not that clear.
Suggested change Line 347: 'This means that, surprisingly, most of the….'
Suggested change line 349: 'Overall, the changes in the PfMD3 mRNA targets were also observed at the protein level where a greater proportion of proteins were significantly decreased for Stage II (decreased 24; increased ??) and Stage V (decreased 27; increased ???) gametocytes (Figure S6B).'
This section would benefit by keeping the clear message that it is the protein products of the 66 bound transcripts that are being discussed (e.g. Line 361 '…., the protein products') being discussed or not.

Results, Line 199 onwards: One of the strengths of this paper is the number of transcriptomics, proteomics and variations thereof applied and how these are used to gain insight into PfMD3 function. However, this also means there is a lot of data and hypothesis generation to digest through this long section of the results. It is encouraged for the authors to look for ways to simplify and link their story better.
An example of how it becomes a challenge to follow the text through here is the number of potential functions for this one protein that are indicated. E.g.:
Line 215: may be a mis-regulation of gametocyte maturation in ΔPfMD3 or that there may be more female transcripts represented in ΔPfMD3 compared to NF54e.
Line 232: the transcript abundance of male-specific gametocyte markers is decreased beginning at Stage II of development onward, which supports the observed reduction in fertile male gametocytes produced by the ΔPfMD3 strains (Figure 2F).
Line 260: Taken together, our proteomics results indicate that PfMD3 is important for the proper regulation of proteins involved in sexual maturation and fertility.
Line 266: suggesting that translational dynamics impacting protein synthesis are also being affected by PfMD3.
Line 292: These results suggest that mRNAs turnover might be an important function in developing male gametocytes.
Line 362: were significantly decreased compared to the wild-type (Figure 5A), suggesting that PfMD3 may be necessary for the proper translation of these mRNAs.
Line 378: transcripts bound by PfMD3 were preferentially being translated into proteins and are not being degraded or stabilized.
Line 417: Interaction with these proteins that have putative roles in RNA transport from the cytoplasm into the nucleus suggests that PfMD3 may also function as an mRNA chaperone.
Line 421: Therefore, PfMD3 may bind certain mRNAs as they are being translated.
Line 428: Taken together, these data suggest that PfMD3 is a multi-functional protein that can interact with mRNA-Protein (mRNP) complexes as well as binding directly to mRNAs to facilitate their export from the nucleus to the cytoplasmic ribosomes, ultimately enhancing translation.
Line 504: the PfMD3-protein interaction data supports a multi-functional role for PfMD3 in male-specific translation potentially mediating efficient shuttling of mRNA targets out of the nucleus through interaction with nuclear pore proteins to enable translation by direct interaction with the mRNA translation complex.
The authors have provided a lot of really nice data to demonstrate that PfMD3 is a busy protein. It is suggested that the authors put together a summary Figure of their findings that demonstrate simply and clearly the functions and interactions indicated by the data. It would also be good to see whether descriptions and text can be simplified.

Discussion: It is surprising that there is no discussion about the work by Farrukh et al. (Ref 63) on the same protein in the discussion, particularly around broad similarities and differences in the BioID dataset of Farrukh et al. (Ref 63) and this study.

Minor comments

Line 18: Repeated word, 'from host to host'. Perhaps consider changing this to something such as 'between hosts' or 'from host to vector'.

Line 35: Remove the comma between 'parasite' and 'which'.

Line 36: 'Female anopheline mosquito'. Change to 'female anopheles…' or 'anopheline mosquito'.

Line 257: remove 'either'.

Line 277: 'they' instead of 'the'.

Line 289: Have these RNA regulating enzymes been specifically linked to gametocytogenesis before?

Line 311: Is there another way to demonstrate the frequency of occurrence. Stating the total number of occurrences

throughout the genome does not seem that helpful to assess this with out some sort of comparator.

Line 457: 'Genetic disruption of PfMD3 (ΔPfMD3) did not reduce the overall number of male gametocytes but decreased the number of exflagellating males, which consequently resulted in a drastic reduction of all stages of development beyond oogony.'
Figure 1D suggests a 50% reduction in Sexual stage parasite commitment. So is this statement correct then? Is it that the overall numbers drop but the sex ratio stays the same.

Line 517: 'Female' rather than 'male'.

Line 517: The following sentence seems contradictory between the 'lack of extensive regulation; but there still being 'highly coordinated production'.
"Likely, the rapid production of these simple cells does not allow for extensive regulation of macromolecular (protein and RNA) synthesis suggesting there is a highly coordinated production of the transcripts and proteins needed for gametogenesis.'

Line 523: In the following sentence, if PfMD3 is found to be expressed in asexual stages, how can it be male specific?
'we have provided an in-depth analysis of the molecular mechanism of the male-specific RNA-binding protein, PfMD3'

Line 546: Sentence clarity, '….Pennsylvania State University for their contributions…..'

Lines 815, 935, 945: space between number and SI unit.

Figure 1C and 1E: Scale annotation is difficult to read on some images with the 'm' partially cut off, potentially by the black border around the individual images.

Figure 1E and 1F, lines 1407 – 1423: labelling does not match what is stated in the figure legend (E and F are the wrong way around in the figure).

Reviewer #2

(Remarks to the Author)
I co-reviewed this manuscript with one of the reviewers who provided the listed reports. This is part of the Nature Communications initiative to facilitate training in peer review and to provide appropriate recognition for Early Career Researchers who co-review manuscripts.

Reviewer #3

(Remarks to the Author)
This manuscript by Riëtte et al. investigates the role of the CCCH-type zinc finger protein PfMD3 in Plasmodium falciparum gametocyte development, proposing that it functions as a male-specific translational regulator during gametocytogenesis. The authors apply a multi-omics approach，including RNA-seq, CLIP-seq, proteomics, and CRISPR-based gene editing，to support their conclusions. The key finding suggests that PfMD3 enables the accumulation of proteins required for male gamete formation, contrasting with the translational repression mechanisms commonly observed in female gametocytes.
Despite the breadth of data presented, the manuscript is significantly weakened by several critical issues. The mechanistic insight into PfMD3's function remains vague, and many of the results are internally inconsistent, insufficiently validated, or correlative rather than causative. Key claims are not supported by direct experimental evidence (e.g., motif function, domain activity, translational activation).
Importantly, the novelty of the work is overstated. A recent study (Farrukh et al., 2024) has already described PfMD3's role in male fertility and its interaction with RNA regulatory machinery. This prior work is not meaningfully acknowledged, compared, or contrasted in the current manuscript. Additionally, PfMD3 is not discussed in relation to other well-characterized male regulators, such as PfMD1, PfMD4, and PfMD5 (PyRBPm1), whose established roles in translational control during male gametocytogenesis are highly relevant to this study.
In the absence of functional validation (e.g., domain mutagenesis, motif disruption, rescue experiments) and with limited integration of existing literature, the conceptual foundation of the manuscript remains incomplete. Substantial revisions are necessary to clarify the contribution of PfMD3 to gametocyte biology and to distinguish this work from overlapping prior studies.

Major Comments
1. Lack of Mechanistic Clarity: The core claim that PfMD3 promotes translation via binding to GAAGAA-rich RNA motifs is not convincingly supported. While CLIP-seq identifies an enriched motif, no functional validation is provided. EMSA data lack specificity, as scrambled RNA probes bind similarly, and competition assays do not clearly demonstrate specific motif recognition. The authors do not show whether PfMD3 interacts with ribosomal components, initiation factors, or polysomes. Furthermore, no reporter assays or mutational studies are used to confirm motif-dependent translation. The role of the CCCH-type zinc finger domain is not dissected.
2. Expression Contradictions: The authors claim that PfMD3 is male-specific, but IFA and western blot results show strong

expression in schizont-stage asexual parasites. Quantification is based on too few cells (n=15–20), and no female-specific marker (e.g., Pfg377) is used to exclude expression in female gametocytes. The evidence for sex-specificity is therefore weak and potentially misleading.
3. Inadequate Functional Validation: The CRISPR KO strategy targets only the first two exons of PfMD3 (a 485-aa protein), leaving the possibility that the C-terminal region may retain function. The absence of rescue or complementation experiments makes it difficult to assign observed phenotypes specifically to PfMD3 disruption. The upregulation or downregulation of transcripts and proteins in the knockout strain is not validated by qRT-PCR or western blot. The distinction between direct and indirect effects remains unclear.
4. Dataset Inconsistencies and Limited Integration: The overlap between datasets is minimal. Only four proteins overlap between CLIP-seq and IP-MS (Fig. 6B), and proteomic changes often do not correlate with transcriptomic changes. The experimental timepoints used across datasets are inconsistent. Replicate variation is high (e.g., in IP-MS data). Such inconsistencies raise concerns about data robustness and target specificity.
5. Incomplete Comparative Context: The manuscript does not position PfMD3 within the broader family of male-specific RNA regulators such as PfMD1, PfMD4, and PfMD5 (PyRBPm1), which have known roles in translational control during gametocyte development. The lack of comparison with these factors obscures PfMD3's unique or overlapping functions. Without such context, the claim of novelty is significantly undermined.
6. Overstated Novelty: Farrukh et al. (Mol Microbiol, 2024) has already demonstrated that PfMD3 is critical for male gamete fertility and physically interacts with PUF1, ALBA3, ZNF4, and CITH. These results are not thoroughly discussed. The authors' claim that PfMD3 is the first male-specific translational regulator is inaccurate and disregards both Farrukh's work and previously known factors like PfMD1. The current study does not offer deeper biochemical insight or identify direct translational targets convincingly.
7. Ambiguous Phenotypic Results: Several results are reported without proper interpretation or alignment with previous work. For instance, the increased asexual replication rate upon PfMD3 deletion contradicts earlier findings and lacks mechanistic explanation. The upregulation of AP2-FG and HDP1 is noted but not discussed, despite their known relevance. The authors report changes in male gametocyte protein abundance, but do not explore whether this affects downstream stages like ookinete formation or mosquito infectivity beyond oocyst counts.

Detailed Comments
1. Title Inconsistency: The manuscript title ("The CCCH-type zinc-finger PfMD3 regulates translation needed for malaria parasite transmission") differs from the supplementary title ("The PfMD3 CCCH-type zinc-finger protein acts as an enhancer of protein translation for malaria parasite transmission"). These should be harmonized to accurately reflect the study's mechanistic depth and avoid overstating the function.
2. Lines 42–44: The rationale for targeting PfMD3 for malaria control is not well explained. The authors should justify the significance of translational regulation in male gametocytes as a viable or novel intervention strategy.
3. Lines 73–75: The conclusion regarding protein translation during sexual differentiation relies on an outdated 1979 reference. Authors should update this with recent data (e.g., single-cell RNA-seq) supporting translational control in gametocyte development.
4. Lines 110–111: Misrepresentation of Specificity. The claim that PfMD3 is solely expressed in male gametocytes is not supported by western blot and IFA results (Fig. 1C/D), which clearly show expression in schizonts. The authors should revise this claim and quantify expression more rigorously.
5. Lines 113–116: Confusing Role. Although PfMD3 KO reduces male-specific transcript/protein abundance, it does not reduce male gametocyte numbers. The authors should clarify whether PfMD3 is a differentiation marker, a translation regulator, or both, and what its direct biological role is, or does it function prior to Stage II ?
6. KO Design and Functionality: The CRISPR knockout targets only the first two exons of the 485-aa PfMD3 protein. The retained C-terminal region may still be functional. A full gene knockout or domain deletion is needed, and complementation is essential to confirm phenotypes.
7. Lines 145–148: Inadequate Cell Count: Only 15–20 gametocytes were counted to determine sex specificity. This is insufficient, and a female marker such as PfG377 should be used to distinguish cell types accurately.
8. Lines 160–162: Unexplained Growth Phenotype, an increase in asexual multiplication in PfMD3 KO parasites is reported but not mechanistically explained. FACS analysis, transcriptomics. And the phenotype was contrasts with Farrukh et al. (2024)'s conditional KD strategy, comparison with published data is needed.
9. Lines 173–174: Inconsistent Maturation Claim: The claim that PfMD3 does not impact gametocyte maturation contradicts earlier results showing molecular disruptions. This needs clarification.
10. Figures 2F, H, I: Statistical Ambiguity, no statistical information (e.g., replicates, error bars, p-values) is provided. The authors should detail the analysis and annotate figures appropriately.
11. Lines 195–196: Cross Outcome Unclear, the ΔPfMD3 × ΔMACFET cross yielded normal oocyst numbers, despite ΔMACFET being deficient in gametocyte development. This result needs further explanation.
12. Lines 210–211: Unexplored Expression Changes, AP2-FG and HDP1 are upregulated in PfMD3 KO parasites, but the authors do not interpret their relevance. These genes are functionally important in gametocytogenesis and should be discussed.
13. Lines 227–228: qPCR Validation Missing, RNA-seq is based on only two replicates. There is no RT-qPCR validation of key genes. Moreover, Figure 3A and 3B use the same dataset, which is methodologically circular.
14. Lines 228–230, 250–252: RNA-Protein Discordance, Numerous female gametocyte transcripts (e.g., P47) are unchanged at RNA level but decreased at the protein level. These inconsistencies are not explored or validated.
15. Lines 253–261: Timing Claims Not Validated, The authors propose that PfMD3 misregulates the timing of protein expression, but provide no validation by IFA or WB over developmental timepoints.
16. Lines 295–298: RNA-Binding Domain Unclear, PfMD3's RNA-binding domain is not dissected. A CCCH-domain deletion or domain swap could clarify binding specificity.
17. Lines 323–326: Weak Motif Enrichment, The GAAGAA motif is common across the genome, with modest enrichment (p

= 3.2e-4 to 6.7e-6). EMSA results show similar binding to scrambled probes and no clear competition. These data do not convincingly demonstrate specific RNA binding.
18. Supplementary Figure S5: Confused Labeling, Legends for S5A and S5B are mismatched. The motifs differ between CLIP-seq and RNA-bind-n-seq, and no p-values are shown for the latter.
19. Lines 335–337: Overinterpretation, The authors overstate conclusions based on modest statistical enrichment and weak binding validation. Statements should be revised for caution and accuracy.
20. Lines 345–352: Binding vs. Function, Of 207 CLIP targets, only 66 show protein changes. If PfMD3 regulates translation, more overlap would be expected. The role of the remaining bound RNAs is not explained.
21. Figure 6B and IP-MS Data：Poor Replicate Consistency Only four proteins overlap between IP-MS and CLIP-seq. Replicates vary greatly, and stages (S II-III) used for IP differ from other datasets. A no-tag control strain should be used. Known interactors (e.g., ZNF4, RNF1) were already validated in prior studies.
22. Line 507–509: Core Conclusion Contradicted, The authors admit that direct targets of PfMD3 are unknown, despite extensive multi-omics profiling. The overlap between datasets is minimal. As a result, the central claim of PfMD3 as a sequence-specific translational activator remains unproven, and the novelty of the study is diminished.

Reviewer #4

(Remarks to the Author)
This manuscript presents a dissection of the role of Md3 in male gametocytes. It is a careful and considered study and is an important addition to the knowledge on RNA biology in male gametocytes which is currently being studied by several groups. The study fills an important gap in understanding male-specific translational control during gametocytogenesis. The data is of good quality and the conclusions are in majority supported throughout the manuscript. I have a number of small and a few more substantial comments to improve the manuscript:

Comments:
Introduction
-It’s unclear why the introduction details sexual commitment to this extent as it’s not central to the message of the paper (also oddly repeated in the discussion).
-It is not known how long gametocytogenesis takes in all Laverania species (also in the discussion)
Fig 1
-It is odd that there is no detection of the protein in the asexuals whilst the immunofluorescent image of schizont is compelling.

Fig 2
-The enhanced asexual multiplication rate is very interesting and potentially important in defining the md3 functon(this was not detected in a genome wide screen in Pb). The author imply that this may be due to the lesser sexual commitment of these clones but this is not tested. The authors could culture these parasites with lysoPC or choline to establish an intrinsic differential growth rate.
-Scoring sex ratios by Giemsa is difficult and often inaccurate especially when mutants may display subtle morphological changes, since the authors use the p230 antibody, it is not clear why they have not used that to accurately quantify the sex ratio. This is an important conclusion to validate.
-Commitment is sensitive to cloning out parasites, to my mind it is not sufficient to have reduced commitment in two clones to claim that commitment is affected by the Md3 disruption in the absence of complementation studies. I am not suggesting that this is needed for the paper but the claim is not supported.
-The statement portraying an absence of maturation defect based on simple morphology is also too hasty, giemsa-morphology is very crude measure of proper maturation. Especially given the much more granular understanding that the authors go on to describe
-Exflagellation rate should be corrected for the gametocytemia of the cultures since the overall gametocyte population is reduced.
- A FACs plot example should accompany the ploidy measurement for the nuclear division in the males
-For the crossing experiment, the data is compelling but I would rather present it as the heterologous cross shows enhanced fertilisation in relation to the homologous one as the normal range statement is not compared to any control…
Fig 3
-line 243- mitosis not meiosis
Fig4
For the occurrence of clusters of motifs within MD3-peaks, an IGV example is shown but no global analysis or metric/stat is given.
-its unclear why the EMSA probe has a reduced motif (GAAGAA) rather than the full one.
-For the RBNS, is an enrichment of GAA conclusive enough?
-Line 335 Fig S5A not B.
-legend of S5A and B mixed up
Fig 5
The integration of the data brings the paper nicely together at this stage.
Fig 6
These data are interesting and the IP is interesting, but none of the identifications are validated by another method so some of the conclusions are quite bold in this part and could be transferred to the discussion.

Reviewer #5

(Remarks to the Author)
I co-reviewed this manuscript with one of the reviewers who provided the listed reports. This is part of the Nature Communications initiative to facilitate training in peer review and to provide appropriate recognition for Early Career Researchers who co-review manuscripts.

Version 2:

Reviewer comments:

Reviewer #1

(Remarks to the Author)
Van Biljon et al. provide a revised manuscript that has significantly addressed the concerns we raised, incorporated many of the suggestions and produced a much stronger manuscript. Other than some minor formatting problems, we have no further suggestions or comments on the manuscript.

Minor comments

Line 135: The wording in the following sentence is unclear and we recommend modifying. It implies whole genome sequencing can detect mRNA changes. This seems likely to be dealing with two different datasets (gDNA and mRNA sequencing).

'and whole genome sequencing (Figures S2C) which revealed a loss in pfmd3 mRNA abundance in gametocytes by RNA sequencing from ΔPfMD31.1 (Figure S2D, Table S1).'

Line 1625: 'with its male a development defect'. Is this meant to read 'with a male…..'?

Reviewer #2

(Remarks to the Author)
I co-reviewed this manuscript with one of the reviewers who provided the listed reports. This is part of the Nature Communications initiative to facilitate training in peer review and to provide appropriate recognition for Early Career Researchers who co-review manuscripts.

Reviewer #3

(Remarks to the Author)
In this revised manuscript, the authors have addressed most major concerns raised in the previous reviewing with additional experimental data or reanalysis. Though a few concerns are still not well-resolved, I feel that the main conclusions of the current version are solid, and important for the related field in malaria research. Thus I would recommend to publish it on Nature Communications.

Reviewer #4

(Remarks to the Author)
The authors have done a thorough revision based on all other reviewers' comments. I have two further comments on the revised items:
The authors have made a significant effort by incorporating a single-cell dataset, but they don't exploit it to its full potential. It would be useful to incorporate a representation of developmental stages in each branch. The authors state "cells map along the entire male developmental trajectory"—this is too vague. We need to see the sampling of cells at different differentiation stages. It would also be useful to isolate the male branch and plot the integrated object without the MCA data to gauge the qualitative difference of the parasites during differentiation. There is a missed opportunity to perform dynamic DE in pseudotime to understand the timing of the MD3 effect. I think the authors should also include a comparison of other stages to contextualize the potential role (even if not essential) at other developmental stages, including in stalk cells.
L166. This suggests that the overall decrease in gametocytemia is due to a sex-specific impact of PfMD3 in males. I don't think this is shown by the data. The male/female bifurcation is observed around stage III, yet the loss is already seen in stage II. There is also still a question of the numbers here: the % of gametocytes that are PF47+ or P230+ in the KO don't add up to 100%, suggesting the presence of potentially double-negative parasites (although I understand this is not double-stained). I am not suggesting new experiments but the authors should be more cautious.

Reviewer #5

(Remarks to the Author)
I co-reviewed this manuscript with one of the reviewers who provided the listed reports. This is part of the Nature Communications initiative to facilitate training in peer review and to provide appropriate recognition for Early Career Researchers who co-review manuscripts.

Version 3:

Reviewer comments:

Reviewer #4

(Remarks to the Author)
The authors have addressed all my comments, I would like to congratulate them on a very nice piece of work.

Open Access This Peer Review File is licensed under a Creative Commons Attribution 4.0 International License, which permits use, sharing, adaptation, distribution and reproduction in any medium or format, as long as you give appropriate credit to the original author(s) and the source, provide a link to the Creative Commons license, and indicate if changes were made.
In cases where reviewers are anonymous, credit should be given to 'Anonymous Referee' and the source.
The images or other third party material in this Peer Review File are included in the article's Creative Commons license, unless indicated otherwise in a credit line to the material. If material is not included in the article's Creative Commons license and your intended use is not permitted by statutory regulation or exceeds the permitted use, you will need to obtain permission directly from the copyright holder.
To view a copy of this license, visit https://creativecommons.org/licenses/by/4.0/

Reviewer #1 (Remarks to the Author):

The CCCH-type zinc-finger PfMD3 regulates translation needed for malaria parasite transmission by van Biljon et al presents an in-depth characterisation of PfMD3 across the P. falciparum asexual and gametocyte stages. The authors multi-omics approach has identified PfMD3 as 'the first male-associated regulator of translation during gametocytogenesis'. This work builds on the finding of Farrukh et al. (Ref 63) who previously undertook a similar phenotypic analysis of PfMD3. Biljon et al. characterisation of PfMD3 KOs impact on transmission, identification of the RNA binding motif and characterisation of this proteins role in facilitating translation of male gametocyte specific genes builds significantly beyond our current understanding of this proteins function.

This work provides important insight into malaria parasite gene and protein regulation for which there are still many unknowns and identifies potential avenues for future drug and vaccine exploration. This paper is generally well-written and enjoyable to read. Reasoning and conclusions are generally well-supported. Some experimental outcomes need to be clarified and the text around the outcomes of some of the 'omics' experiments could be improved as the message is being lost.

We are grateful to the reviewer for their positive assessment of our manuscript and for recognizing that our multi-omics characterization of *Pf*MD3 indeed builds upon other recent work and extends our knowledge of the role of *Pf*MD3 in malaria parasite gene regulation. We appreciate the identification of areas where our experimental results required further elaboration. To address this, we have revised the presentation of our results with enhanced data visualization, additional statistical analyses, and improved methodological descriptions to ensure greater clarity and reproducibility. Regarding the "-omics" data communication, we have streamlined the presentation of RNA-seq, CLIP-seq, and proteomics results to highlight key biological insights. We also improved the narrative flow between datasets and have included summary statements to clearly state the biological significance of our findings. We believe these revisions have substantially improved the readability of our manuscript and depict the scientific rigor of our study. We have responded in line to each of your comments below.

Major comments:

Lines 144-146, Figure 1D, S1C and S1D: The authors state that PfMD3 expression peaks in early gametocytes (stages II – IV) however there is only samples for stages II and V on this western blot image. Suggest re-wording to exclude mention of stage 'IV'. Or change 'IV' to 'V' as both stages look to have a very similar level of expression (based on S1D also).

Thank you for correcting this error. We have now amended the text by changing "stage II-IV" to "stage II" in the text (line 122) to match the data presented in Figure 1D.

Data and interpretation could be strengthened here by completing additional replicates for the time course western blots (n=3), as it appears there is only one replicate represented here. There is also minimal banding evident for the ring, troph, schizont fractions, yet the conclusion is that the protein is expressed across the

lifecycle. While it is true that the protein is more obvious on the IFAs and in the western blot of Farrukh et al. (Ref 63), the text implies (Line 143 and Line 151) that Fig 1D and S1D demonstrate expression in asexual blood stages, and it is not at all clear that it is meaningfully expressed in these stages from this data.

We agree that the data for the western blot data demonstrating protein expression could be strengthened with additional replicates. The blot included in Figure 1D was a representative example of one of two biological replicates, in which equal concentrations of protein lysate were loaded per sample. We have now added a third biological replicate and increased the asexual lysate sample concentration to 10x that of the gametocyte lysate to ensure that the *Pf*MD3 protein is detectable on the western blot (amended in Figure 1D and S1C). Although *Pf*MD3-HA is present throughout the asexual stages, this does not establish the functional relevance of *Pf*MD3 in these stages, particularly given that the gene encoding *Pf*MD3 can be knocked out, implying that it is not essential for asexual blood stage development. Therefore, in this study, we focus on the role of *Pf*MD3 during mid- to late-stage gametocytogenesis, where it has been shown to be essential. While this does not preclude a possible role in other stages of development (e.g. asexual blood stages), we note that only ~30% of asexual stage parasites expressed *Pf*MD3-3xHA as detected by IFA.

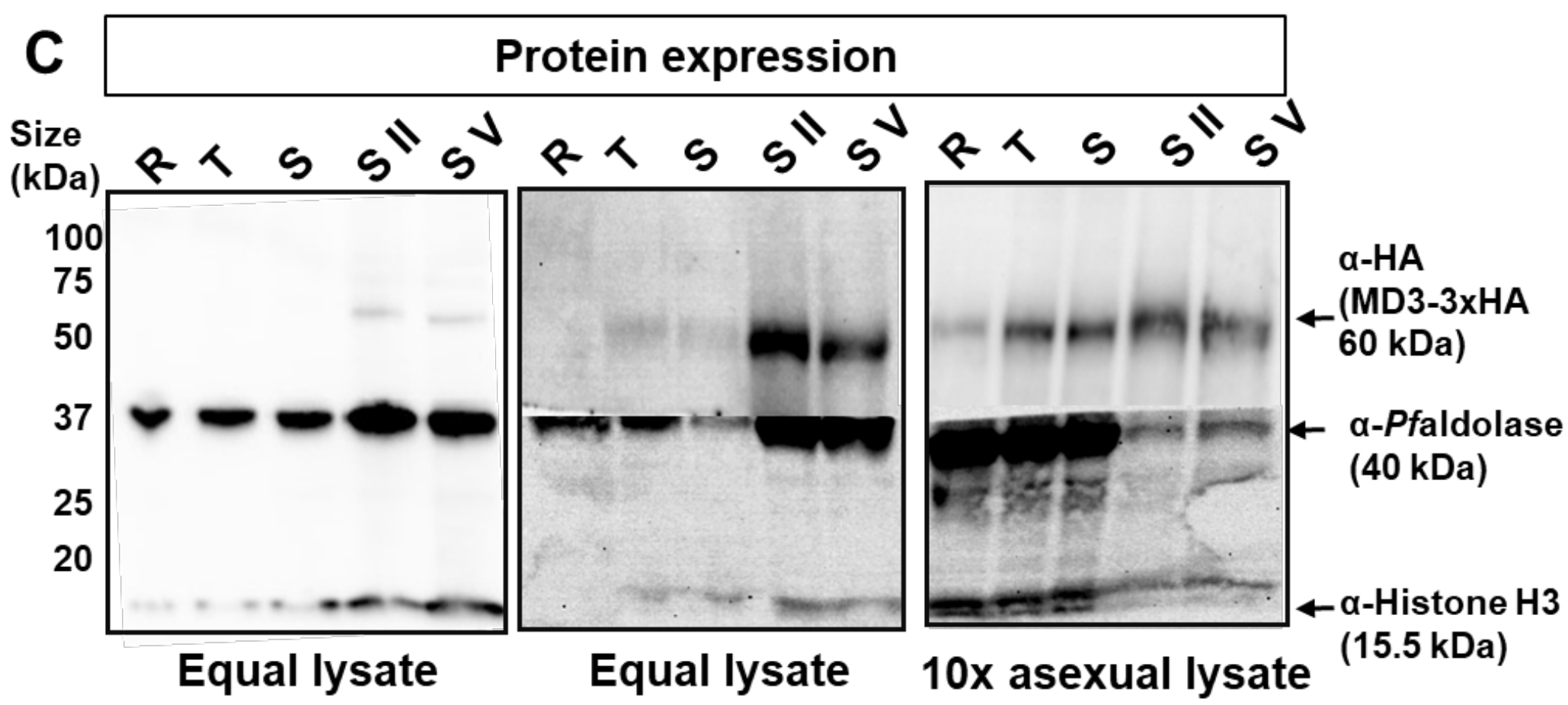

The distinction between nuclear and cytoplasmic extracts is unclear in S1C. It would help to describe what is being addressed here, it is not even mentioned in the Figure Legend. What stages are represented in the fractionation blots? The PfMD3 bands are barely visible in these blots, and the aldolase bands are visible in both extracts, so it would be better to repeat or remove than leave as is.

We agree that the cellular fractionation results do not significantly add to the paper and we have decided to remove this result in the newly revised manuscript.

Figure 2B, C, D: The idea that the increased asexual parasite growth seen for PfMD3 KO parasites is a result in reduced commitment to gametocytemia (2C, 2D) is nice. Some things to consider.

1) If the assay conditions between 2B and 2C are equivalent, then it should be possible to mathematically assess whether the lost gametocyte production in 2C is equivalent to the increased asexual parasite growth seen in 2B. Would you actually expect a 50% reduction in sexual stage commitment to result in a near 20% increase in asexual parasite growth? That would imply 30-40%

of your asexual stage parasites are converting every cycle I think, which seem high for general in vitro parasite culture.

2) If the assays are not equivalent (e.g. 2B is a standard growth assay whereas 2C is a gametocyte production that relies on stressing the parasites to induce higher rates of gametocyte commitment) then it would difficult to assess such proportionality between the two assays. But likewise, the pressures leading to gametocytogenesis differ between the assays.

Other possible reasons for increased growth are not covered, such as a change in nutrient acquisition, protein expression, or cloning artefacts. So while the idea is nice, the argument would be strengthened if proportionality could be indicated and perhaps some other possible reasons explored or discounted around how the assays are done.

We thank the reviewer for their thoughtful comments regarding this interesting result and we have carefully considered the possible reasons proposed for the increased growth in the Δ*Pf*MD3 parasite strain. With respect to the growth assessments performed for Figures 2B and 2C, the two assays are not equivalent for the reason the reviewer specifies: while gametocyte commitment was induced under stress conditions, asexual replication was measured under standard, non-stress culture conditions. We agree with point #2 that directly comparing the multiplication rate and sexual commitment experiments to assess proportionality in the growth phenotypes is not straightforward.

To address the limitations of the assays performed in our original submission and to strengthen the assertion that the modest increase in asexual multiplication rate observed in the Δ*Pf*MD3 line is attributable to a reduction in sexual commitment, we created a conditional *Pf*MD3 knockdown (KD) line. The *Pf*MD3 KD line allows us to control for potential confounding factors—including cloning artefacts, differences in nutrient acquisition, and variation in protein expression—that may arise from comparing two distinct parasite strains. To create a this conditional knockdown strain, an inducible *glmS* ribozyme (PMID: 24023691), activated by the addition of glucosamine, was inserted at the 3' end of the gene. Growth phenotypes were then assessed in the presence and absence of glucosamine (i.e., under knockdown and non-knockdown conditions, respectively). This KD system recapitulates the growth phenotype observed in the Δ*Pf*MD3 line, with a modest increase in asexual replication and a decrease in sexual commitment in the presence of glucosamine (new Figures S3C and S3D, shown below).

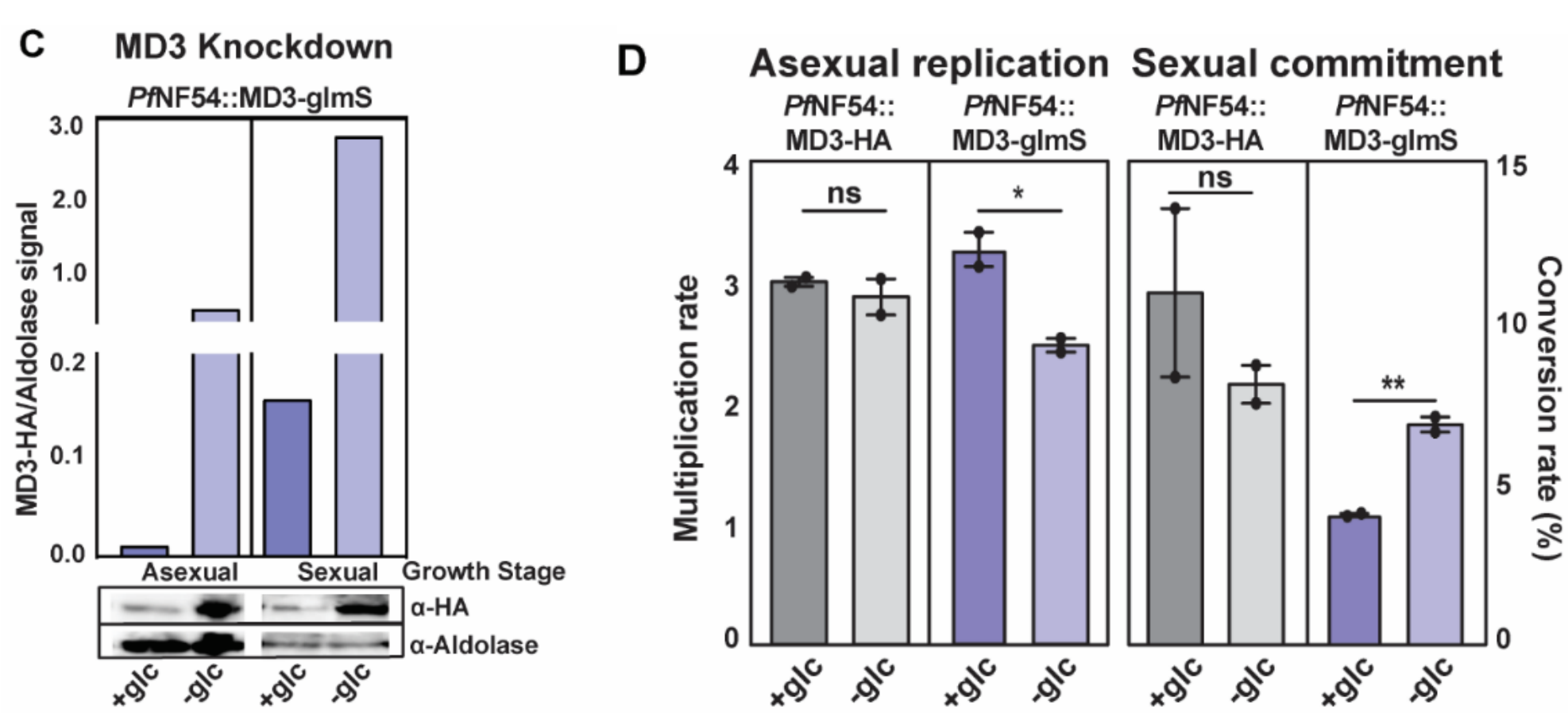

To directly quantify asexual multiplication rates in the wild-type and Δ*Pf*MD3 parasite strains, parasites were cultured in the presence of choline, which prevents sexual commitment (PMID: 29129376) and thereby enables the direct measurement of asexual replication. Following incubation of both the wild-type NF54 and Δ*Pf*MD3 in the presence of choline—starting at the ring stage and continuing for a full cycle to capture reinvasion—no significant difference in multiplication rate was observed between the two strains (Figure S2F). This finding suggests that the increase in asexual multiplication observed standard culture conditions (i.e., without choline; Figure 2B) is primarily due to the reduction in sexual commitment in the absence of *Pf*MD3. These results are presented on page 6 and lines 148-151.

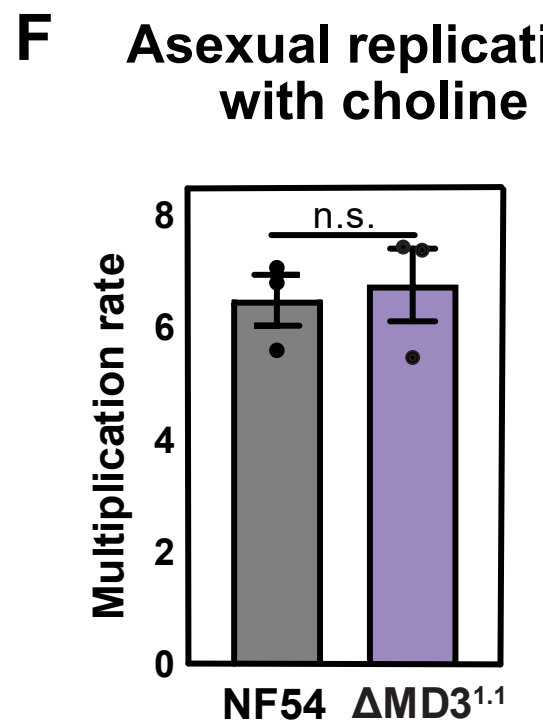

Line 345 onwards: The text through this section is difficult to follow and trying to keep track of what protein sets are being discussed is not that clear. Suggested change Line 347: 'This means that, surprisingly, most of the….' Suggested change line 349: 'Overall, the changes in the PfMD3 mRNA targets were also observed at the protein level where a greater proportion of proteins were significantly decreased for Stage II (decreased 24; increased ??) and Stage V (decreased 27; increased ???) gametocytes (Figure S6B).' This section would benefit by keeping the clear message that it is the protein products of the 66 bound transcripts that are being discussed (e.g. Line 361 '…., the protein products') being discussed or not.

We have edited the text in the section noted by the reviewer to clearly distinguish when we are referring to proteins as opposed to mRNA transcripts.

Results, Line 199 onwards: One of the strengths of this paper is the number of transcriptomics, proteomics and variations thereof applied and how these are used to gain insight into PfMD3 function. However, this also means there is a lot of data and hypothesis generation to digest through this long section of the results. It is encouraged for the authors to look for ways to simplify and link their story better.

We agree that a more concise synthesis of the various multi-omics datasets presented would enhance overall clarity. To address this, we have reduced discussion of the numerous hypotheses emerging from these data and have revised the relevant text and figures (Figures 3 and 5) to better convey a more focused, cohesive narrative.

An example of how it becomes a challenge to follow the text through here is the number of potential functions for this one protein that are indicated. E.g.:

Line 215: may be a mis-regulation of gametocyte maturation in ΔPfMD3 or that there may be more female transcripts represented in ΔPfMD3 compared to NF54e.

Line 232: the transcript abundance of male-specific gametocyte markers is decreased beginning at Stage II of development onward, which supports the observed reduction in fertile male gametocytes produced by the ΔPfMD3 strains (Figure 2F).

Line 260: Taken together, our proteomics results indicate that PfMD3 is important for the proper regulation of proteins involved in sexual maturation and fertility.

Line 266: suggesting that translational dynamics impacting protein synthesis are also being affected by PfMD3.
Line 292: These results suggest that mRNAs turnover might be an important function in developing male gametocytes.
Line 362: were significantly decreased compared to the wild-type (Figure 5A), suggesting that PfMD3 may be necessary for the proper translation of these mRNAs.

Line 378: transcripts bound by PfMD3 were preferentially being translated into proteins and are not being degraded or stabilized.

Line 417: Interaction with these proteins that have putative roles in RNA transport from the cytoplasm into the nucleus suggests that PfMD3 may also function as an mRNA chaperone.

Line 421: Therefore, PfMD3 may bind certain mRNAs as they are being translated.

Line 428: Taken together, these data suggest that PfMD3 is a multi-functional protein that can interact with mRNA-Protein (mRNP) complexes as well as binding directly to mRNAs to facilitate their export from the nucleus to the cytoplasmic ribosomes, ultimately enhancing translation.

Line 504: the PfMD3-protein interaction data supports a multi-functional role for PfMD3 in male-specific translation potentially mediating efficient shuttling of mRNA targets out of the nucleus through interaction with nuclear pore proteins to enable translation by direct interaction with the mRNA translation complex.

The authors have provided a lot of really nice data to demonstrate that PfMD3 is a busy protein. It is suggested that the authors put together a summary Figure of their findings that demonstrate simply and clearly the functions and interactions indicated by the data. It would also be good to see whether descriptions and text can be simplified.

> The reviewer's point is well taken. To address this, we have clarified the observations underlying the statements identified by the reviewer, simplified the presentation of the results, and constructed a model figure to more clearly illustrate the functional roles of *Pf*MD3 during gametocytogenesis (Figure 6D, shown below) in the revised manuscript.
>
> Specifically, we have incorporated new results from single cell RNA-sequencing of the Δ*Pf*MD3 line, which allows for a more accurate assessment of changes in the male and female gametocyte populations. These new finding have led to revised conclusions at Lines 215 and 232. By integrating these results with the knockout proteomics (Lines 260 and 266), CLIP-seq (Lines 285, 362 and 376) and IP-MS (Lines 412 and 453-462), we have constructed an over-arching mechanistic model. Specifically, the IP-MS results reveal that *Pf*MD3 interacts with components of the pre-ribosome and U3 snRNPs in the nucleolus suggesting a role in ribosomal maturation. Additionally, *Pf*MD3 interacts with proteins involved in shuttling mRNA

from the nucleus to the cytoplasm (AMPD, THO2), as well as other nuclear pore proteins (NUP313, 335, 434), potentially in a transient association during mRNA is export. Our CLIP-seq further identified several *Pf*MD3-associated mRNAs that are important for gametogenesis—including *ap2-o2, md4/arid, p230,* and *not1-g*—and that are decreased in the *Pf*MD3-knockout proteome. Taken together with the observed association of *Pf*MD3 with translational machinery, these findings suggest that *Pf*MD3 is required for the adequate translation of these essential factors.

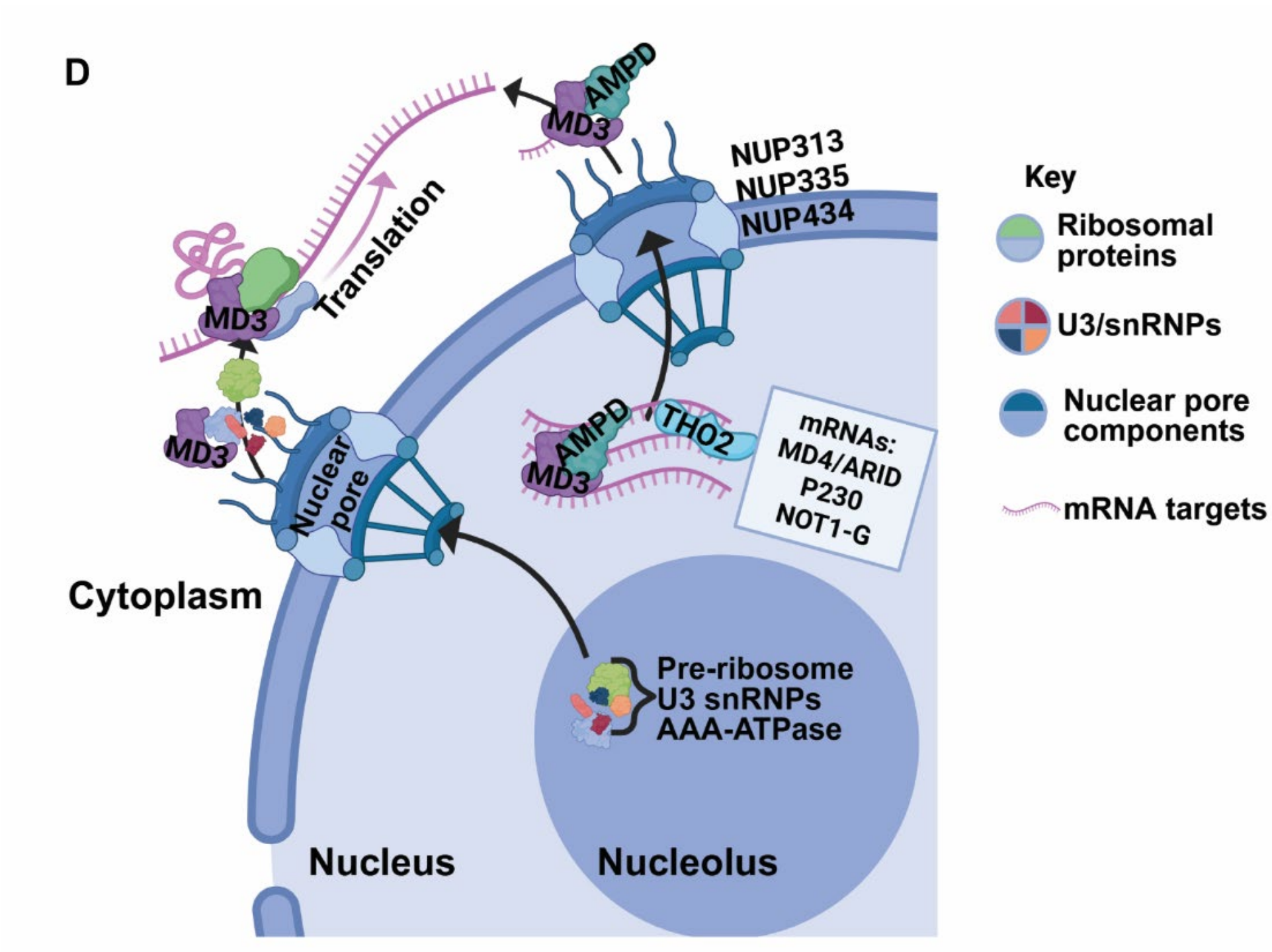

Discussion: It is surprising that there is no discussion about the work by Farrukh et al. (Ref 63) on the same protein in the discussion, particularly around broad similarities and differences in the BioID dataset of Farrukh et al. (Ref 63) and this study.

We are certainly aware of this work and do reference these finding throughout the manuscript, but agree that a more in-depth comparison with Farrukh et al. was warranted in both the Results and Discussion sections. To address this, we have now added discussion of the differences we observed between our IP-MS dataset and the BioID dataset of Farrukh et al, and have noted the overlap between the two datasets in the Results.

Specifically, consistent with Farrukh et al., we find *Pf*MD3 associated with other zinc finger proteins (lines 421-424, page 15): "Two interactors, both zinc finger domain containing proteins (ZNF4: PF3D7_1134600 and RNF1: PF3D7_0314700), were previously identified and verified via reciprocal immunoprecipitation[72], providing independent validation of our IP-MS approach (Figure 6B)."

However, the postulated association between *Pf*MD3 and translational repressors reported by Farrukh et al. is not supported by our data. We address this discrepancy in our Discussion section in line 519-524 page 18. "Our proposed role for *Pf*MD3 in interacting with translational machinery and other zinc finger

proteins (ZnF4, RNF1) is consistent with published data for *Pf*MD3[72]. However, our findings do not support a role for *Pf*MD3 as a translational repressor as previously reported[72]. Rather, our multi-omics analyses and complementary *in vitro* methods demonstrate *that Pf*MD3 targets are actively translated and that loss of *Pf*MD3 decreases protein production."

We further note that Farrukh et al. expressed *Pf*MD3-BioID using a predominantly female promoter (*fnpa* also known as *lap5*), which may introduce a sex-specific bias in expression. This could result in an artificial increase in *Pf*MD3 expression in female gametocytes, where translational repression is a prominant feature of gene regulation. Without expression of *Pf*MD3-BioID from its endogenous promoter, it is therefore difficult to determine whether the interactions reported by Farrukh et al. are genuine biological associations restricted to the female gametocyte or are attributable to differences in experimental difference.

Minor comments:

Line 18: Repeated word, 'from host to host'. Perhaps consider changing this to something such as 'between hosts' or 'from host to vector'. We have made the requested change to "between hosts".

Line 35: Remove the comma between 'parasite' and 'which'. Removed.

Line 36: 'Female anopheline mosquito'. Change to 'female anopheles…' or 'anopheline mosquito'. Changed as requested.

Line 257: remove 'either'. We removed this sentence from the paper as part of streamlining the discussion of our multi-omics results as requested.

Line 277: 'they' instead of 'the'. Removed "the".

Line 289: Have these RNA regulating enzymes been specifically linked to gametocytogenesis before?

We thank the reviewer for this question. As noted later in the manuscript (lines 382 and line 544), NOT1-G has been characterized as important for male gamete development in *P. yoelii* (PMID: 34673764), while the other three enzymes—NOT-1, DCP1 and DCP2—have not yet been functionally characterized during gametocytogenesis. However, all three have been detected in gametocyte transcriptomes and proteomes (PMID: 27298255, PMID: 20332084).

Line 311: Is there another way to demonstrate the frequency of occurrence. Stating the total number of occurrences throughout the genome does not seem that helpful to assess this without some sort of comparator.

We appreciate the reviewer's comment regarding the need for better contextualization of our frequency analysis and agree that the raw occurrence counts alone provide limited interpretive value without an appropriate comparator. To address this, we now present the occurrence of the GAA trinucleotide and the full AWGAAGAA consensus sequence within the CLIP-seq peaks themselves compared to the full transcriptome and incorporated these results into Figure S5C and S5D (shown below). We have also added the following interpretations to the results section (line 323-330 page 11): "Strikingly, the vast majority of the bound transcripts contained a similar enriched motif, AWGAAGAA, at both Stage II

(136/143 mRNA targets; $p = 6.7 \times 10^{-6}$) and Stage V (84/86 mRNA targets; $p = 3.2 \times 10^{-4}$) (Figure 4C), occurring at a frequency above the transcriptome-wide background (8415 occurrences in transcripts, www.Plasmodb.org). Closer examination revealed that this motif occurred in repeated clusters within 66 of the 207 enriched regions identified by CLIP-seq to be bound by *Pf*MD3 (Figure 4D, Figure S5C), suggesting that the AWGAAGAA sequence repeats may be preferred over single GAA trinucleotide occurrences."

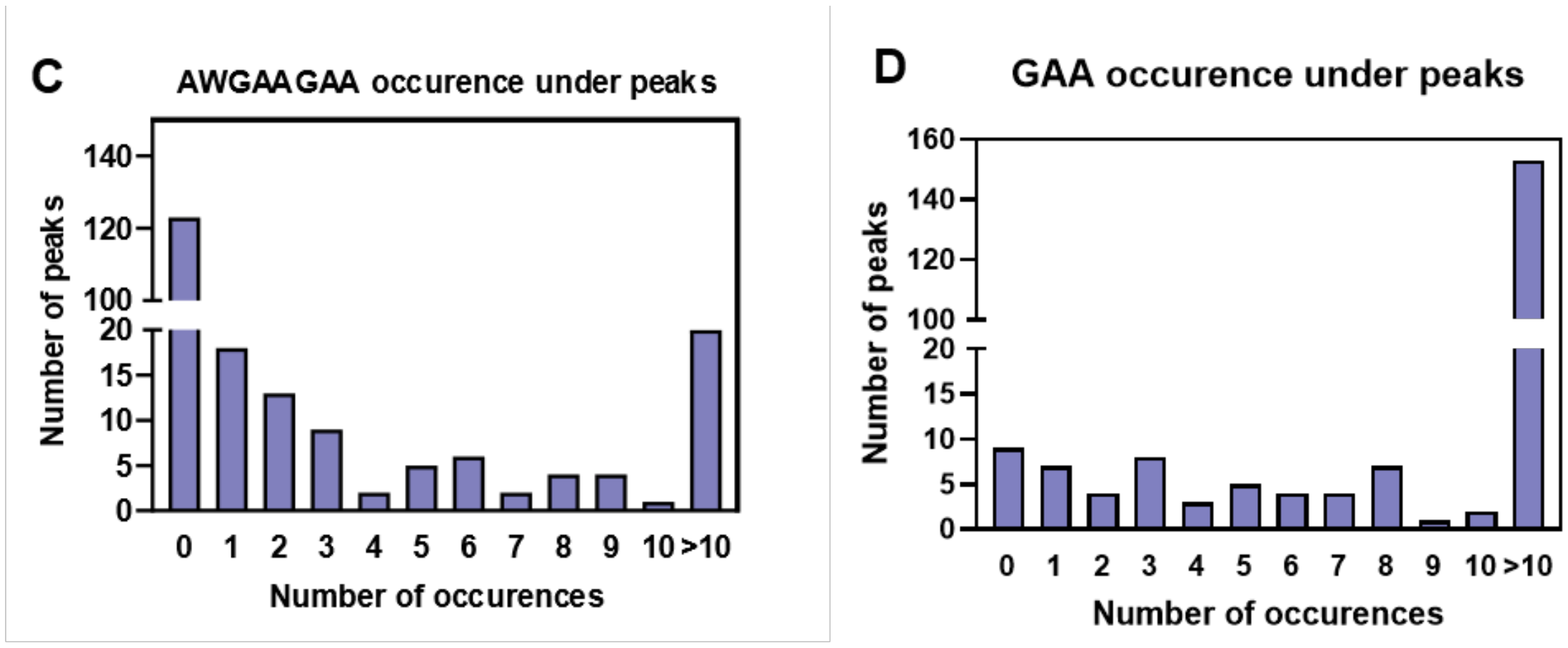

Line 457: 'Genetic disruption of PfMD3 (ΔPfMD3) did not reduce the overall number of male gametocytes but decreased the number of exflagellating males, which consequently resulted in a drastic reduction of all stages of development beyond oogony.'

Figure 1D suggests a 50% reduction in Sexual stage parasite commitment. So is this statement correct then? Is it that the overall numbers drop but the sex ratio stays the same.

We thank the reviewer for this important request for clarification regarding the interpretation of sexual stage commitment data presented in Figure 1D. In response to a related comment from another reviewer noting that morphology alone is not always a reliable method for determining gametocyte sex, we have performed new experiments in which P230p and P47 antibodies were used to enumerate male and female gametocytes, respectively. These experiments reveal that, although the Δ*Pf*MD3 gametocytes appeared morphologically normal by Giemsa staining, there is a pronounced reduction in P230p-positive male gametocytes in the Δ*Pf*MD3 compared to the wild-type, with no corresponding reduction in P47-positive female gametocytes (see below and amended Figure 2E). We have revised our conclusions throughout the manuscript to reflect this selective reduction in mature male gametocytes upon disruption of *Pf*MD3.

E

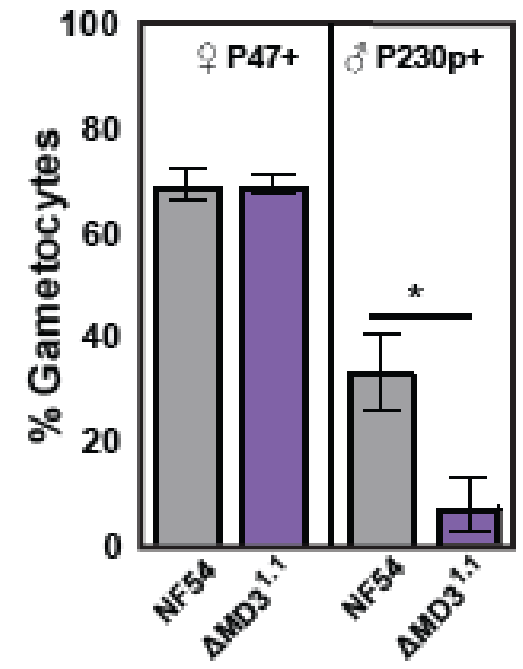

Line 517: 'Female' rather than 'male'. This line is meant to specifically contrast male gametocyte development from male gametogenesis and is not referring to female gametocytes. No changes have been made.

Line 517: The following sentence seems contradictory between the 'lack of extensive regulation; but there still being 'highly coordinated production'.

"Likely, the rapid production of these simple cells does not allow for extensive regulation of macromolecular (protein and RNA) synthesis suggesting there is a highly coordinated production of the transcripts and proteins needed for gametogenesis."

> We thank the reviewer for identifying this contradiction and have revised the sentence accordingly. We intended to contrast the rapid nature of gamete development which does not permit extensive regulation, with the prolonged development period afforded by *P. falciparum* gametocytogenesis. The sentence has been revised (Lines 406-407) as follows "The expression of *Pf*MD3 in gametocytes thus allows for the sufficient production of proteins involved in male gamete production and suggests that the accumulation of these proteins during gametocyte development is crucial prior to the rapid and energy-intensive process of male gamete production[97]."

Line 523: In the following sentence, if PfMD3 is found to be expressed in asexual stages, how can it be male specific?
'we have provided an in-depth analysis of the molecular mechanism of the male-specific RNA-binding protein, PfMD3'

> Thank you for the recommendation to be more precise with our description of *Pf*MD3. We amended our statements to be more accurate regarding expression of *Pf*MD3.

Line 546: Sentence clarity, '….Pennsylvania State University for their contributions…..' We have provided clarification for this description.

Lines 815, 935, 945: space between number and SI unit. Amended, as requested.

Figure 1C and 1E: Scale annotation is difficult to read on some images with the 'm' partially cut off, potentially by the black border around the individual images. We have edited the scale annotation to be more readable.

Figure 1E and 1F, lines 1407 – 1423: labelling does not match what is stated in the figure legend (E and F are the wrong way around in the figure). Thank you for catching these errors. We have corrected them.

Reviewer #2 (Remarks to the Author):

I co-reviewed this manuscript with one of the reviewers who provided the listed reports. This is part of the Nature Communications initiative to facilitate training in peer review and to provide appropriate recognition for Early Career Researchers who co-review manuscripts.

Reviewer #3 (Remarks to the Author):

This manuscript by Riëtte et al. investigates the role of the CCCH-type zinc finger protein PfMD3 in Plasmodium falciparum gametocyte development, proposing that it functions as a male-specific translational regulator during gametocytogenesis. The authors apply a multi-omics approach， including RNA-seq, CLIP-seq, proteomics, and CRISPR-based gene editing， to support their conclusions. The key finding suggests that PfMD3 enables the accumulation of proteins required for male gamete formation, contrasting with the translational repression mechanisms commonly observed in female gametocytes.
Despite the breadth of data presented, the manuscript is significantly weakened by several critical issues. The mechanistic insight into PfMD3's function remains vague, and many of the results are internally inconsistent, insufficiently validated, or correlative rather than causative. Key claims are not supported by direct experimental evidence (e.g., motif function, domain activity, translational activation).
Importantly, the novelty of the work is overstated. A recent study (Farrukh et al., 2024) has already described PfMD3's role in male fertility and its interaction with RNA regulatory machinery. This prior work is not meaningfully acknowledged, compared, or contrasted in the current manuscript. Additionally, PfMD3 is not discussed in relation to other well-characterized male regulators, such as PfMD1, PfMD4, and PfMD5 (PyRBPm1), whose established roles in translational control during male gametocytogenesis are highly relevant to this study.

In the absence of functional validation (e.g., domain mutagenesis, motif disruption, rescue experiments) and with limited integration of existing literature, the conceptual foundation of the manuscript remains incomplete. Substantial revisions are necessary to clarify the contribution of PfMD3 to gametocyte biology and to distinguish this work from overlapping prior studies.

> We appreciate the comprehensive evaluation of our manuscript and for highlighting important areas that require clarification and further elaboration. While we are surprised at the general negative tone of the review, we recognize the validity of a number of the concerns raised and have incorporated the constructive feedback into our revised manuscript. We believe that the additional experiments motivated by this reviewer and edits to the current manuscript have increased the clarity of the functional role of *Pf*MD3 and distinguished this work from prior studies.

We also acknowledge that our initial manuscript insufficiently addressed the recent work by Farrukh et al. (2024) and failed to provide sufficient context within the broader landscape of male gametocyte regulators. We have therefore revised the manuscript to:

1. Include a detailed discussion directly comparing our findings with Farrukh et al. (2024), clearly delineating overlapping observations from novel contributions specific to our study.
2. We have added substantial discussion of *Pf*MD1, *Pf*MD4, and *Pf*MD5 (*Py*RBPm1) and their established roles in male gametocyte translational control, positioning *Pf*MD3 within this regulatory framework.
3. We have revised our overall manuscript to more accurately reflect the advances of our contributions to the understanding of the role of *Pf*MD3 as presented in the Farrukh et al. manuscript, while highlighting the specific mechanistic insights revealed by our multi-omics approach.

We recognize the reviewer's concerns regarding mechanistic depth and have addressed these through:

1. We have included additional experiments where we studied DNA sequence motif binding specificity and inactivation of the zinc finger domain in lieu of motif deletion and phenotypic rescue experiments for our *Pf*MD3 knockout using a newly generated *pfmd3* conditional knockdown line.
2. We have strengthened the integration between our RNA-seq, CLIP-seq, and proteomics datasets to provide more robust conclusions regarding the effect of *Pf*MD3 on translation.
3. We have carefully combed the data for inconsistencies and provided additional validation where necessary.

Lastly, the reviewer noted that our mechanistic model requires further substantiation. To address this, we have:

1. Refined our translational activation model by adding direct experimental evidence to support the role of *Pf*MD3 in protein translation versus transcriptional effects.
2. Provided RNA sequence motif function validation by including additional experiments to demonstrate the functional significance of these RNA binding motifs.

We believe these revisions substantially address the reviewer's concerns and provide a rigorous foundation for our conclusions. We encourage the reviewer to evaluate the novelty of the methods used to understand the molecular role of *Pf*MD3 and note that this is the first time that direct RNA-binding activity has been characterized in *Plasmodium falciparum.* As such, this study paves the way for the characterization of hundreds of other RNA-binding proteins that are also likely essential for parasite development and transmission.

We have replied to each comment from the reviewer in-line below.

Major Comments:

1. Lack of Mechanistic Clarity: The core claim that PfMD3 promotes translation via binding to GAAGAA-rich RNA motifs is not convincingly supported. While CLIP-seq identifies an enriched motif, no functional validation is provided. EMSA data lack specificity, as scrambled RNA probes bind similarly, and competition assays do not clearly demonstrate specific motif recognition. The authors do not show whether PfMD3 interacts with

ribosomal components, initiation factors, or polysomes. Furthermore, no reporter assays or mutational studies are used to confirm motif-dependent translation. The role of the CCCH-type zinc finger domain is not dissected.

We assert that both the RBNS and EMSA *in vitro* experiments provide functional validation of our *in vivo* CLIP-seq findings that *Pf*MD3 binds to GAAGAA-rich RNA. To further support these claims, we have conducted additional RBNS and EMSA experiments using both an inactivated (through EDTA-based Zn depletion) *Pf*MD3 CCCH-zinc finger domain and additional RNA probes. These experiments conclusively demonstrate that GAA-specific RNA binding is mediated by the *Pf*MD3 zinc finger domain (See updated Figure 4F, G).

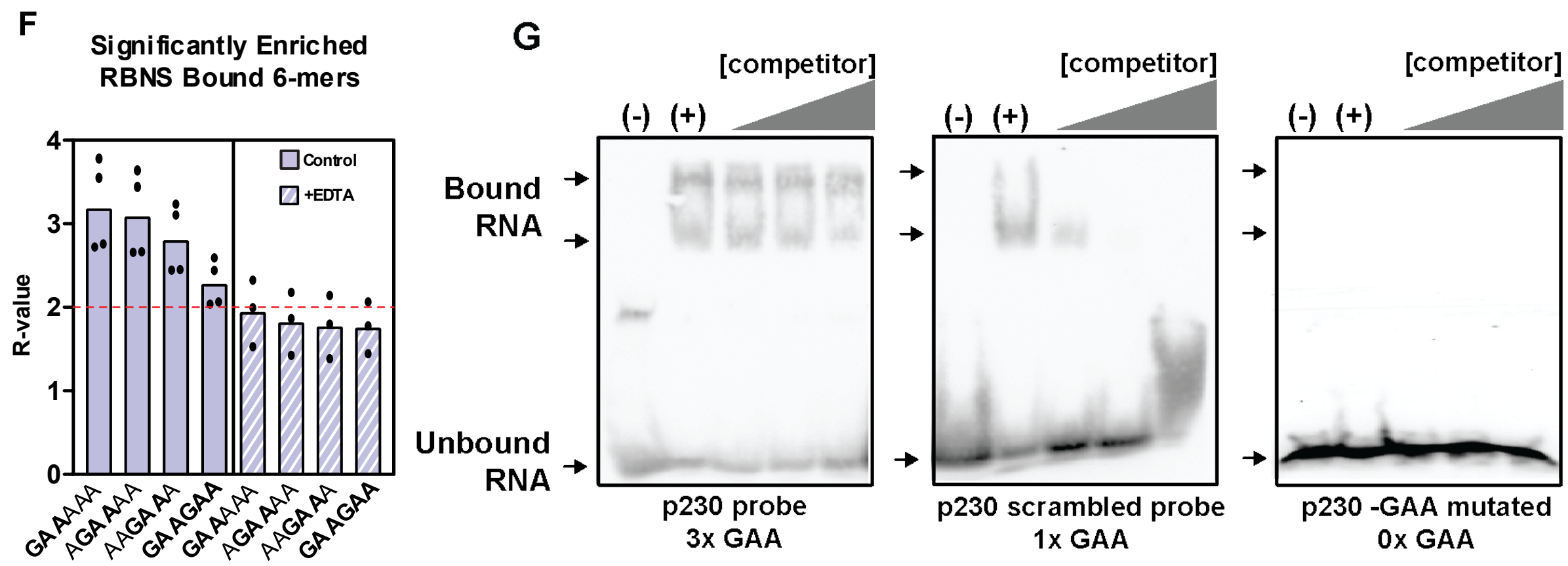

To show that *Pf*MD3 interacts with ribosomal components or initiation factors, we performed protein pulldown assays to identify an interaction between *Pf*MD3 and ribosome biogenesis component MPP10, as well as AMP-deaminase (Figure S6C, D). Additionally, we performed polysome sucrose gradient fractionation to demonstrate that *Pf*MD3 associates with early fractions containing mRNPs and translating monosomes (Figure 6C). Finally, a reporter assay measuring translation activity using wild-type NF54 and Δ*Pf*MD3 parasite lysates revealed a decrease in overall translation in the knockout line (Figure 5D). We believe that these additional experiments support the functional validation of *Pf*MD3 in the promotion of translation.

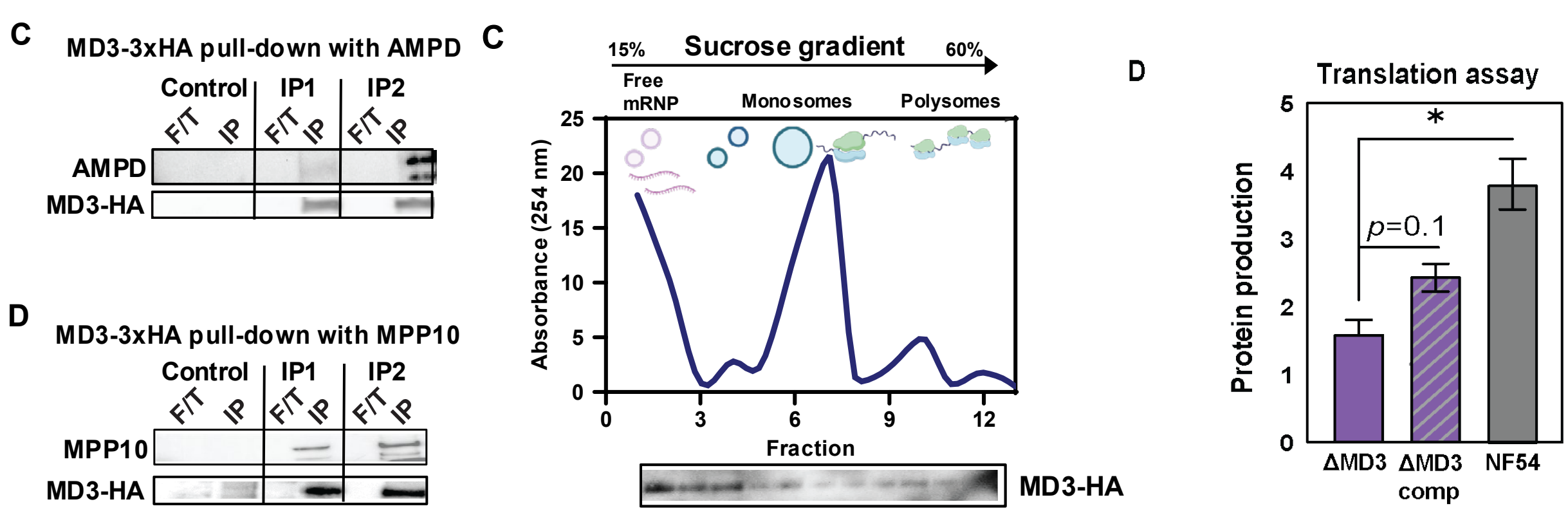

2. Expression Contradictions: The authors claim that PfMD3 is male-specific, but IFA and western blot results show strong expression in schizont-stage asexual parasites. Quantification is based on too few cells (n=15–20),

and no female-specific marker (e.g., Pfg377) is used to exclude expression in female gametocytes. The evidence for sex-specificity is therefore weak and potentially misleading.

We recognize that the presentation of the data may have been unclear, and we have revised the text accordingly to explicitly state that the *Pf*MD3 is expressed in both asexual blood stages and gametocytes (line 127-129, page 5): "Taken together, these results demonstrate that *Pf*MD3 is expressed throughout both the asexual and sexual blood stages but is most highly expressed in male gametocytes."

Regarding quantification, our original analysis was based on gametocytes counted from two independent biological replicates of at least 30 gametocytes per replicate, presented as the average number of P230p-positive (male) gametocytes expressing *Pf*MD3. As described in our response to Reviewer 1, we have now included additional experiments using a female marker (P47) to assess sex-specificity more rigorously. Quantification across two biological replicates encompassing a total of 67 P230p-positive and 66 P47-positive gametocytes reveals that *Pf*MD3 is detected in all male cells, while only a small fraction of female cells express detectable amounts of the protein. These findings are now presented in Figure 1E and 1F (shown below) and discussed on page 5 (Lines 124-127).

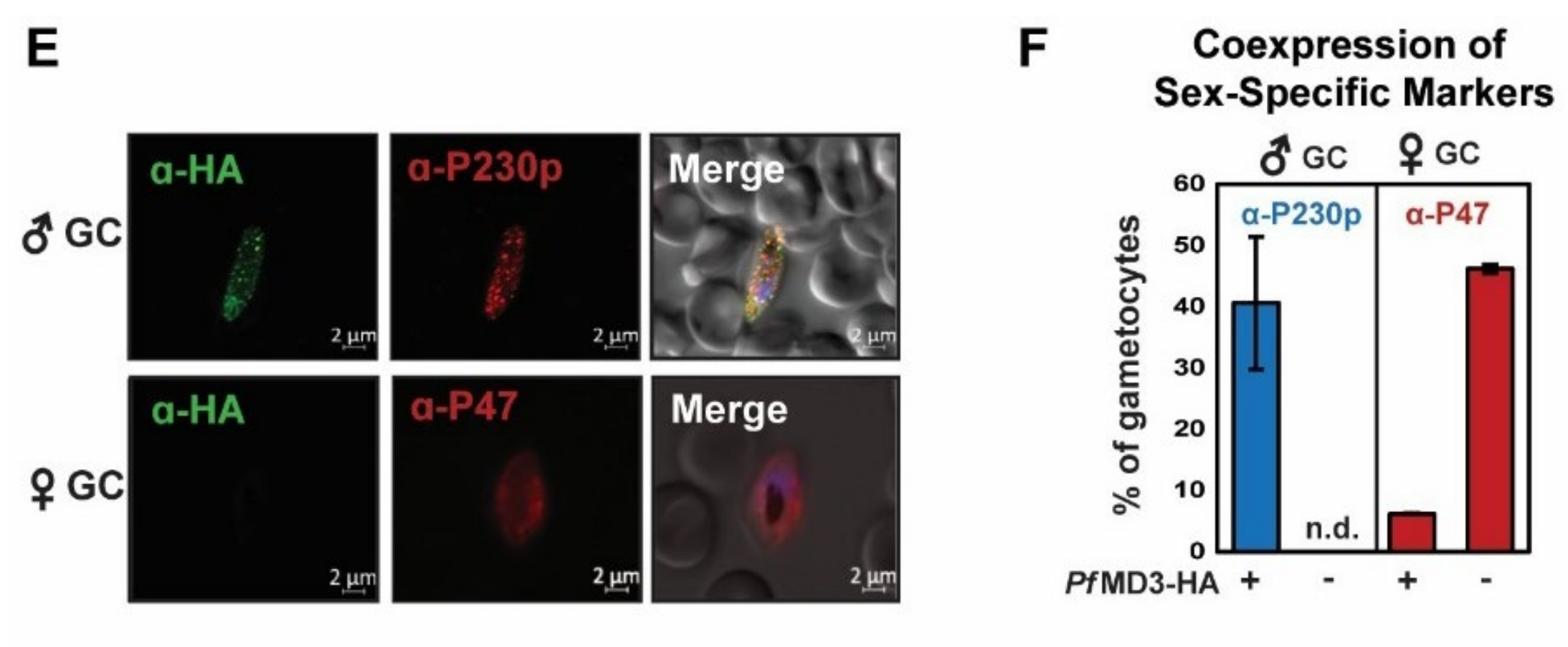

3. Inadequate Functional Validation: The CRISPR KO strategy targets only the first two exons of PfMD3 (a 485-aa protein), leaving the possibility that the C-terminal region may retain function. The absence of rescue or complementation experiments makes it difficult to assign observed phenotypes specifically to PfMD3 disruption. The upregulation or downregulation of transcripts and proteins in the knockout strain is not validated by qRT-PCR or western blot. The distinction between direct and indirect effects remains unclear.

We thank the reviewer for raising this concern regarding our knockout strategy. The genetic disruption of *Pf*MD3 was carefully designed using CRISPR-Cas9, in which the first two codons were replaced with a drug selectable marker and the flanking C-terminal homology region used for repair was modified to introduce a premature stop codon, thereby precluding expression of the remaining coding sequence (shown in the Figure 2A schematic). The absence of any residual C-terminal *Pf*MD3 expression was confirmed by proteomics data from Δ*Pf*MD3 parasites, in which no unique peptides matching the *Pf*MD3 protein were detected, whereas such peptides were readily detected in the wild-type NF54 (Figure S2E). This has now been clarified in the text as follows: "We confirmed the genetic disruption in two clones, Δ*Pf*MD3$^{1.1}$ and Δ*Pf*MD3$^{1.2}$ by genotyping PCR (Figure S2B) and whole genome sequencing (Figures S2C)

which revealed a loss in *pfmd3* mRNA abundance in gametocytes by RNA sequencing from Δ*Pf*MD3[1.1] (Figure S2D, Table S1). We also confirmed that no unique peptides corresponding to *Pf*MD3 were detected by mass spectrometry in the Δ*Pf*MD3[1.1] parasites, while the protein was well-detected in NF54e wild-type parasites (Figure S2E). ”

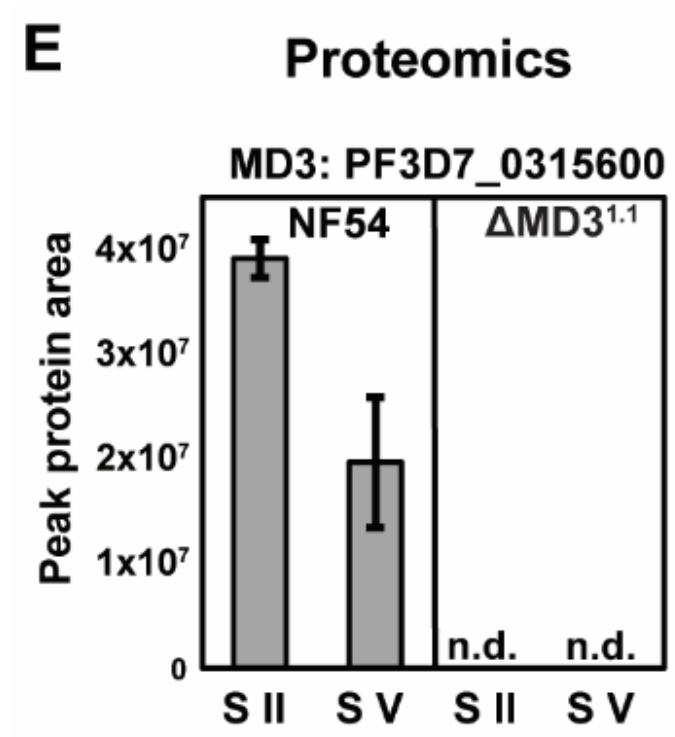

The reviewer is correct that we did not include a secondary method to validate some of the significant changes we see in both the transcriptomics and proteomics of the *Pf*MD3 knockout strain. In this revised manuscript, we have now included RT-qPCR and western blot assessment in Figures S4B and C. We also used RT-qPCR to further verify the down regulation of two mRNAs that encode proteins that have been demonstrated to be critical for male gametocyte development, P230p and MAPK2 (PMID: 30297725, PMID: 32681115). We also verified a decrease in P230 and P47 protein to confirm that we have both male (P230) and female (P47) enriched proteins decreased in our *Pf*MD3-knockout proteome.

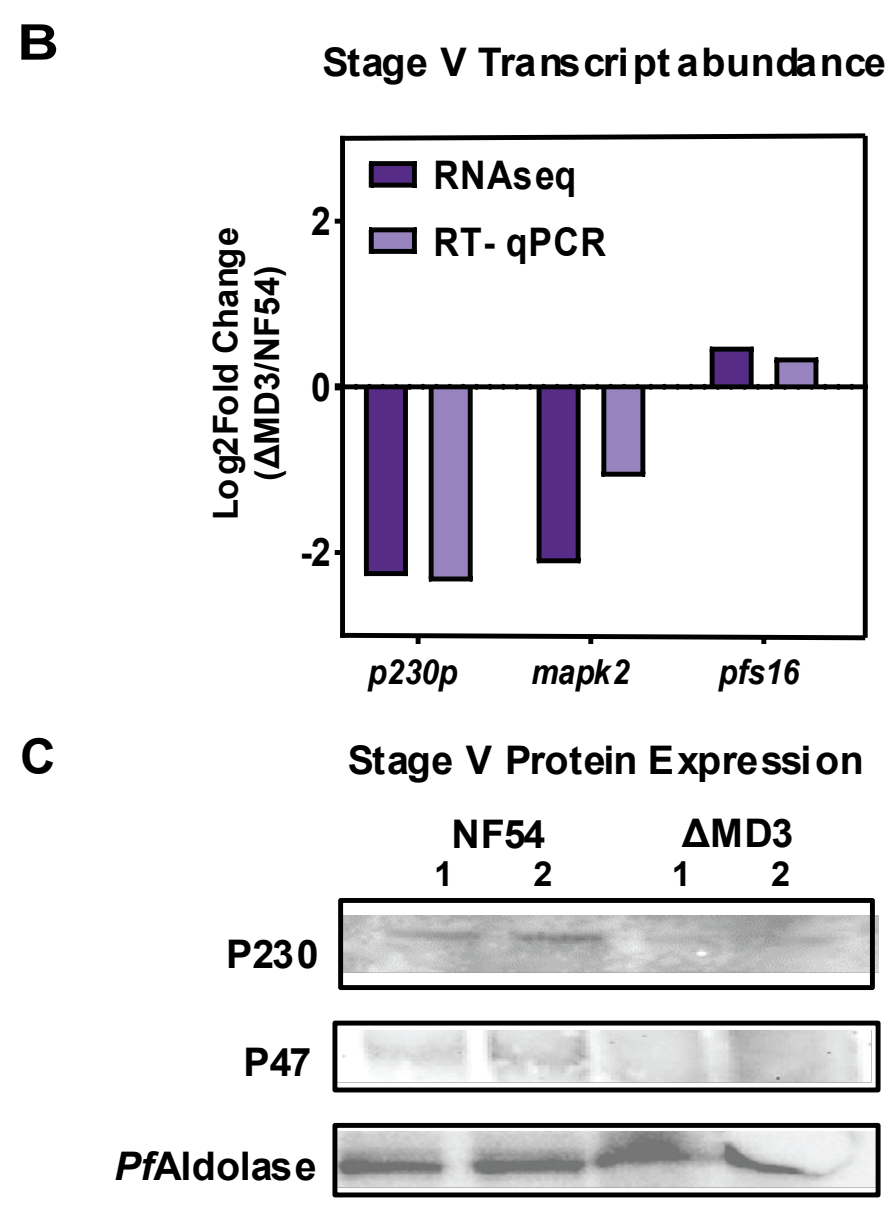

4. Dataset Inconsistencies and Limited Integration: The overlap between datasets is minimal. Only four proteins overlap between CLIP-seq and IP-MS (Fig. 6B), and proteomic changes often do not correlate with

transcriptomic changes. The experimental timepoints used across datasets are inconsistent. Replicate variation is high (e.g., in IP-MS data). Such inconsistencies raise concerns about data robustness and target specificity.

We respectfully disagree with the reviewer's characterization of dataset inconsistencies and maintain that sample collection was coordinated across multiple experiments and that datasets were integrated when appropriate. We provide the following clarifications in response to each specific concern:

1. **Overlap between CLIP-seq and IP-MS** – Since CLIP-seq captures RNA directly bound to *Pf*MD3 and IP-MS identifies proteins associated with *Pf*MD3 in complex, substantial overlap between these two datasets would not be expected, as they capture fundamentally different functional relationships. The limited overlap is therefore not indicative of an inconsistency but rather reflects the distinct nature of each assay.
2. **Proteomic changes not correlating to the transcriptomic changes** – The lack of correlation between our proteomic and transcriptomic datasets is not a result of experimental inconsistency but rather constitutes a major finding of this study. Specifically, it suggests that *Pf*MD3 functions as a post-transcriptional regulator, that influences translation of proteins as opposed to affecting transcript abundance.
3. **Inconsistent timepoints** – All experimental timepoints consist of early-stage (day 5) and late-stage (day 11) gametocyte collections. The one exception is the IP-MS, where we chose to only collect our samples at day 5 where the *Pf*MD3 protein is at peak abundance. The transcriptomics dataset included an additional day 8 timepoint collected early in the study; however, as the day 8 results did not differ significantly from those at day 11, this timepoint did not justify inclusion in subsequent experiments. To avoid confusion, we have removed the day 8 data from the analysis in the revised manuscript.
4. **Replicate variation is high in IP-MS data** – This is among the first to apply DIA-MS for the identification of immunoprecipitated proteins in *Plasmodium*, and to our knowledge, no standardized threshold for inter-replicate variation has been established for this highly sensitive method. DIA-MS identifies many more proteins with high confidence (Q-value <0.01) compared to DDA-MS and, to further increase the confidence in the proteins detected, we restricted our analysis to the 95th percentile of proteins enriched in each sample. Experimental variation was additionally controlled for by applying a stringent threshold requiring significant enrichment in the IP sample across <u>all three</u> experimental replicates. Taken together, our final reporting of *Pf*MD3 interacting proteins is restricted to high confidence results that are consistent among our biological replicates, as is the standard for reporting proteomics data.

5. Incomplete Comparative Context: The manuscript does not position PfMD3 within the broader family of male-specific RNA regulators such as PfMD1, PfMD4, and PfMD5 (PyRBPm1), which have known roles in translational control during gametocyte development. The lack of comparison with these factors obscures PfMD3's unique or overlapping functions. Without such context, the claim of novelty is significantly undermined.

We agree that our original manuscript did not adequately position the role of *Pf*MD3 within the context of other male-specific regulators, and we have now expanded comparisons to *Pf*MD1 and *Pf*MD5 throughout the discussion. However, we respectfully note that the reviewer's characterization of these

proteins as having established roles in translational control during gametocyte development is not fully supported by the literature. Determining the sexual lineage-specific functions of *Plasmodium* proteins remains an active area of research that is in the early stages of investigation by a number of groups, as highlighted by Pradel and Peter (PMID 41107154), which outlines the many remaining knowledge gaps in the field.

Of the proteins mentioned, only PyRBPm1 has been associated with a specific RNA-binding role, which we acknowledge in the introduction (Lines 64-72, Page 3): "Despite the abundance of RNA-binding proteins and evidence supporting RNA-centric mechanisms of gene regulation during the long maturation of *P. falciparum* gametocytes, only a handful of proteins that possess nucleic acid interacting domains have been studied in-depth to date[55-58]. Specifically, Pumilio family (PUF) proteins[46,48,59], an OST-HTH/LOTUS domain containing protein (*Pf*MD1)[60], Male Development Protein 5 (PF3D7_0414500, MD5)[61] also known as RBPm1 in *P. yoelii*[40], the RNA helicase Development of Zygote Inhibited (DOZI)[49], and an Sm-like homolog of worm CAR-I and fly Trailer Hitch (CITH)[44] have all been shown to be essential for sexual maturation and fertilization."

Nevertheless, the *P. falciparum* orthologue of RBPm1 has not been characterized with respect to its RNA-binding function (PMID: 3882412), and since gametocytogenesis is highly different between rodent malaria parasites and *Plasmodium falciparum*, analogous roles cannot be assumed. Similarly, while *Pf*MD1 contains a predicted RNA-binding domain and has been thoroughly characterized phenotypically—including transcriptional profiling of an MD1-knockout—its RNA-binding activity has not been directly characterized (PMID: 36477538). Regarding MD4 (*Pf*ARID), its nucleic acid-binding activity has not been characterized, and is not predicted to be an RNA-binding protein nor to regulate translation, as the ARID domain is a predicted DNA-binding domain (PMID: 35638735). We have endeavored to cite and acknowledge all of these relevant studies for the valuable contributions they make to our understanding of *Pf*MD3, are unable to draw the specific comparisons this reviewer is seeking based on the noted inaccuracies or absence of mechanistic details regarding their roles in RNA regulation in the existing literature.

6. Overstated Novelty: Farrukh et al. (Mol Microbiol, 2024) has already demonstrated that PfMD3 is critical for male gamete fertility and physically interacts with PUF1, ALBA3, ZNF4, and CITH. These results are not thoroughly discussed. The authors' claim that PfMD3 is the first male-specific translational regulator is inaccurate and disregards both Farrukh's work and previously known factors like PfMD1. The current study does not offer deeper biochemical insight or identify direct translational targets convincingly.

The reviewer's point is well taken, and while we do credit the contributions of Farrukh et al. (2024), we did not sufficiently discuss our results in the context of that published study. With respect to the reviewer's criticism regarding our novelty claims in light of prior work on *Pf*MD1, we note that, as described in our previous response, Gomes et al (2022) does not provide a mechanistic basis for male-specific translational regulation during gametocyte development. To address the reviewer's concerns, we have now added a more thorough comparative discussion of *Pf*MD3 and MD1 results in the revised manuscript.

We do, however, contend that our study offers deeper insight into the functional role of P*f*MD3 than the preliminary study by Farrukh et al. Our study employs an endogenously tagged P*f*MD3 protein to capture protein-protein interactions that link the role of *Pf*MD3 in translation through polysome associations. We also characterize the growth phenotypes of both a genetic knockout and a conditional knockdown of PfMD3 across multiple clones and investigate the molecular consequences of this disruption through integrated transcriptomic and proteomic analyses. Most importantly, we provide a full biochemical characterization of the RNA-binding capacity and sequence specificity of the CCCH-type zinc finger domain of *Pf*MD3.

In contrast, Farrukh et al. assessed the effect of *Pf*MD3 knockdown using a single clone generated with a *glmS* knockdown line that achieved only a 37% reduction in protein levels, representing a significant limitation for phenotypic assessment. Furthermore, the BioID approach used by Farrukh et al. expressed tagged *Pf*MD3 under the control of a non-endogenous promoter (*fnpa* AKA *lap5*), which is strongly biased to female gametocyte expression (Lasonder *et al.* 2016, PMID: 27298255 and www.PlasmoDB.org). Expressing *Pf*MD3 outside of its native context with a likely bias toward female gametocytes could result in the capture of artificial protein interactions, including interactions with highly expressed female-specific proteins such as *Pf*CITH. These potential interactions identified by Farrukh et al. were subsequently used to infer a role for *Pf*MD3 in translational repression. However, this proposed function was established by "guilt-by-association" and was never confirmed mechanistically in their study.

7. Ambiguous Phenotypic Results: Several results are reported without proper interpretation or alignment with previous work. For instance, the increased asexual replication rate upon PfMD3 deletion contradicts earlier findings and lacks mechanistic explanation. The upregulation of AP2-FG and HDP1 is noted but not discussed, despite their known relevance. The authors report changes in male gametocyte protein abundance, but do not explore whether this affects downstream stages like ookinete formation or mosquito infectivity beyond oocyst counts.

We thank the reviewer for directing our attention to phenotypic results that were not fully described or interpreted within the results sections of our manuscript. Several of the specific issues raised here are also addressed in greater detail in our responses to comment 8, 12, and 15 below. With respect to exploring the impacts of the loss of *Pf*MD3 downstream of gametocyte development, we have presented data that demonstrates reduced oocyst formation and sporozoite formation, similar to, but not as significant as, the defective exflagellation phenotype. Given this, it is unclear what additional mechanistic insight would be gained from a dedicated investigation of ookinete formation, and this specific request is beyond the scope of the work we present here which focuses largely on the role of *Pf*MD3 during gametocyte development.

Detailed Comments:

1. Title Inconsistency: The manuscript title ("The CCCH-type zinc-finger PfMD3 regulates translation needed for malaria parasite transmission") differs from the supplementary title ("The PfMD3 CCCH-type zinc-finger protein acts as an enhancer of protein translation for malaria parasite transmission"). These should be harmonized to accurately reflect the study's mechanistic depth and avoid overstating the function.

Thank you for catching this error. We have now harmonized our titles between the two documents.

2. Lines 42–44: The rationale for targeting PfMD3 for malaria control is not well explained. The authors should justify the significance of translational regulation in male gametocytes as a viable or novel intervention strategy.

We did not provide a rationale for targeting *Pf*MD3 for malaria control but highlight that there is relevance to understanding its role in the context of the molecular regulation of parasite transmission. Our text reads: "Thus, targeting processes or proteins essential to the formation of gametocytes or the fertilization of gametes presents an effective therapeutic intervention strategy to block parasite transmission[5]." We are trying to convey that gametocytes, as the only transmissible form of the parasite, are an attractive target for preventing disease transmission at this significant "bottleneck" in the lifecycle of *P. falciparum*.

3. Lines 73–75: The conclusion regarding protein translation during sexual differentiation relies on an outdated 1979 reference. Authors should update this with recent data (e.g., single-cell RNA-seq) supporting translational control in gametocyte development.

We include an updated reference for the translational control in gametocytes (PMID: 28531310).

4. Lines 110–111: Misrepresentation of Specificity. The claim that PfMD3 is solely expressed in male gametocytes is not supported by western blot and IFA results (Fig. 1C/D), which clearly show expression in schizonts. The authors should revise this claim and quantify expression more rigorously.

To address the reviewer's concerns and demonstrate the expression of *Pf*MD3 more precisely, we added two more replicates of the western blot (Figure S1C) and included IFA results with the female-specific P47 antibody (Figure 1E, F, both figure panels included earlier in this document on page 13). Based on these results, in addition to the data from the first draft of our manuscript, we have revised the text (Line 127-129, Page 5): "Taken together, these results demonstrate that *Pf*MD3 is expressed throughout both the asexual and sexual blood stages but is most highly expressed in male gametocytes."

5. Lines 113–116: Confusing Role. Although PfMD3 KO reduces male-specific transcript/protein abundance, it does not reduce male gametocyte numbers. The authors should clarify whether PfMD3 is a differentiation marker, a translation regulator, or both, and what its direct biological role is, or does it function prior to Stage II ?

We modified our text to convey our results more accurately (Lines 94-106, Page 4): "We demonstrate that *Pf*MD3 is preferentially expressed in male gametocytes and plays a critical role in male gametogenesis. Genetic disruption of *Pf*MD3 resulted in a pronounced decrease in mosquito transmission and in a reduction of P230p positive male gametocytes. By analyzing the transcriptome and proteome of the *Pf*MD3 knockout parasite line, we found a significant decrease in the abundance of male-associated transcripts and proteins. These findings are corroborated by single cell RNA-sequencing data that demonstrate a reduction in the male lineage of gametocytes lacking *Pf*MD3. We show that *Pf*MD3 is an RNA binding protein that directly binds to a sequence-specific motif in mRNA transcripts that are necessary for male gamete development. Protein-protein interactions with *Pf*MD3 identify an enrichment for RNP biogenesis proteins and components of the translational machinery. Through a

multi-omics integrative analysis of the datasets produced herein, we demonstrate that *Pf*MD3 binds specific mRNA targets to promote their translation to produce fertile male gametocytes. "

We do not claim that *Pf*MD3 is a differentiation marker, though the combination of 'omics' and CLIP-seq and IP-MS data together does point to a regulatory role in translation. We do find the *Pf*MD3 protein expressed in asexual parasites but its expression peaks in early-stage gametocytes, and we see particularly strong expression in male gametocytes. We don't discount the possibility that *Pf*MD3 functions prior to stage II, but for this study it made sense to focus on the stages where *Pf*MD3 is highly expressed and has the largest effect on parasite development. Also, since we and others (PMID: 29724925) have found that *pfmd3* can be genetically ablated, by definition it is non-essential to the asexual bloodstages.

6. KO Design and Functionality: The CRISPR knockout targets only the first two exons of the 485-aa PfMD3 protein. The retained C-terminal region may still be functional. A full gene knockout or domain deletion is needed, and complementation is essential to confirm phenotypes.

We understand the reviewer's concern that the phenotype observed in Δ*Pf*MD3 parasites may be influenced by residual expression of the C-terminal region resulting from our CRISPR-Cas9 disruption design. However, we confirm that there is no residual transcription or translation of the remaining coding region of *Pf*MD3 using RNA-sequencing (Figure S2D) and proteomics (new Figure S2E, provided earlier in this document on page 15). To address the concern regarding complementation, we created a new parasite strain where we placed the expression of *Pf*MD3 under the control of the *glmS* ribozyme (PMID: 24023691), allowing for inducible knockdown upon the addition of glucosamine. Growth phenotypes assessed in the presence and absence of glucosamine recapitulate the findings observed in the Δ*Pf*MD3, including effects on asexual replication, sexual commitment and exflagellation (new Figure S3, see below).

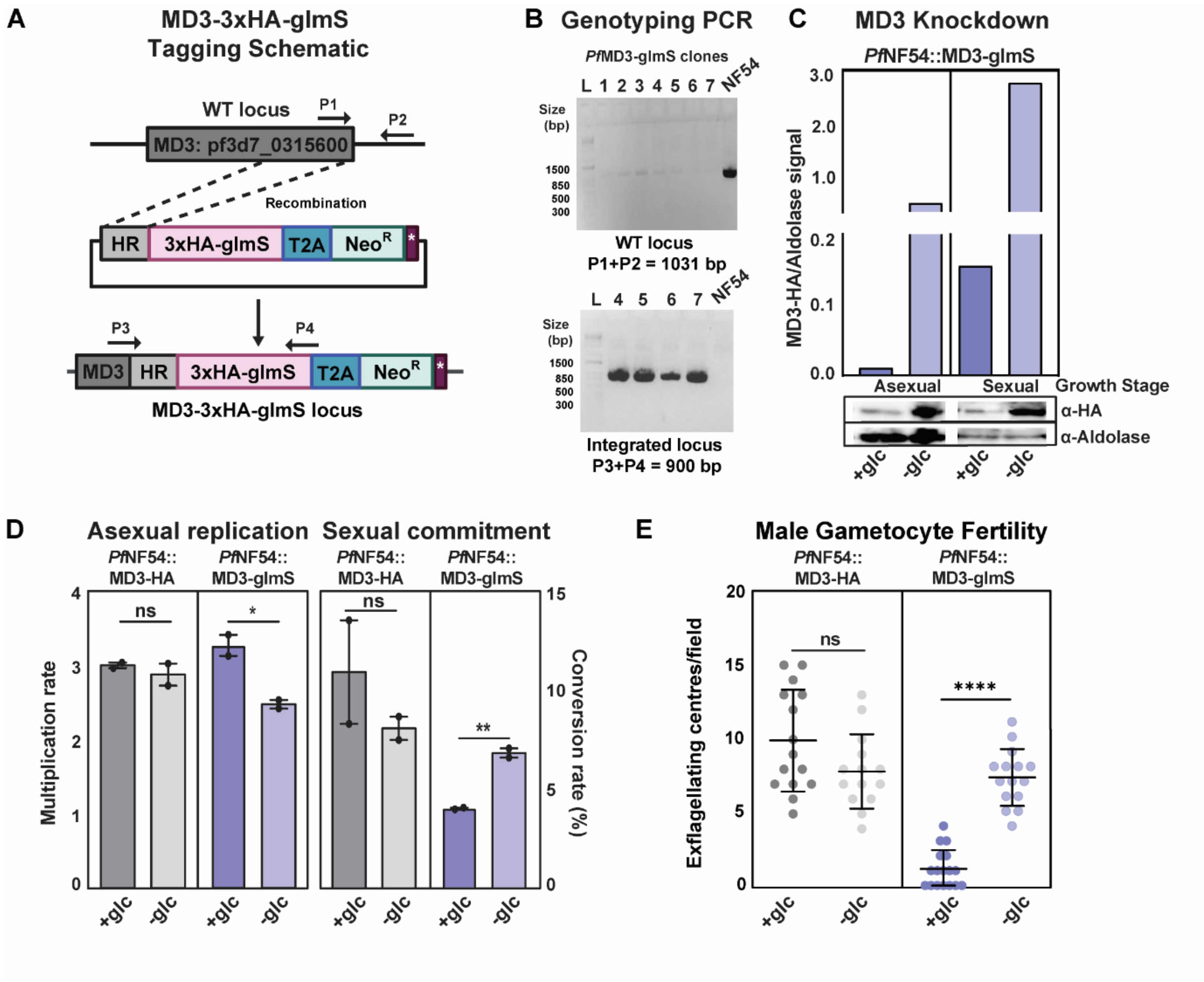

7. Lines 145–148: Inadequate Cell Count: Only 15–20 gametocytes were counted to determine sex specificity. This is insufficient, and a female marker such as PfG377 should be used to distinguish cell types accurately.

> We agree with the reviewer, and we have responded to this specific critique in Major Concern Comment #2 above.

8. Lines 160–162: Unexplained Growth Phenotype, an increase in asexual multiplication in PfMD3 KO parasites is reported but not mechanistically explained. FACS analysis, transcriptomics. And the phenotype was contrasts with Farrukh et al. (2024)'s conditional KD strategy, comparison with published data is needed.

> We acknowledge that the increase in asexual multiplication rate in Δ*Pf*MD3 parasites was not addressed in sufficient detail in the original. The explanation we put forth is that this increase is likely attributable to a reduction in sexual commitment in the absence of *Pf*MD3. To test this directly, we cultured parasites in the presence of choline to prevent sexual commitment, which resulted in equivalent multiplication rates under these conditions (Figure S2F, provided earlier in this document on page 5). Given this finding, we do not consider FACS analysis or assessing the transcriptome of asexual development to be necessary to support this conclusion and we respectfully consider such experiments beyond the scope of the present study.

We agree that our observed phenotype contrasts with the reported phenotype in Farrukh *et al.* However, we present data from our own conditional knockdown line (*Pf*MD3-glmS-3xHA) demonstrating that the phenotype observed in our knockout is recapitulated under knockdown conditions, using 1.25 mM glucosamine to achieve approximately 95% protein reduction (new Figure S3A-E; page 21 in this document).

We determined the optimal concentration of glucosamine to use in our experiments empirically as we could not repeat the knockdown experiments using the same concentration of glucosamine employed by Farrukh *et al.* (5 mM). In our hands, 5 mM glucosamine inhibited the asexual replication of our NF54e strain (shown below), consistent with previously reported toxicity associated with the *glmS* system at higher glucosamine concentrations (PMID: 12419814).

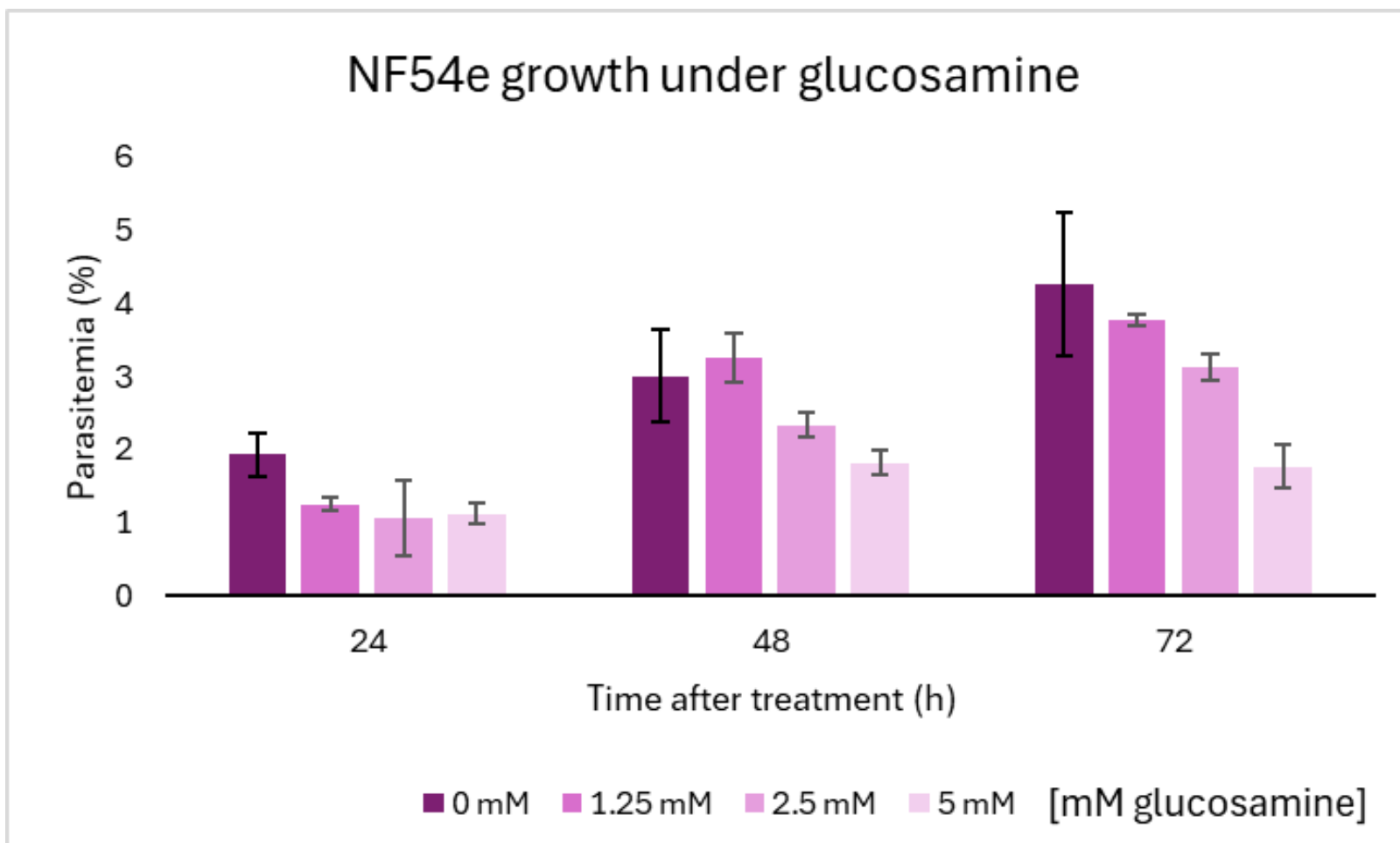

9. Lines 173–174: Inconsistent Maturation Claim: The claim that PfMD3 does not impact gametocyte maturation contradicts earlier results showing molecular disruptions. This needs clarification.

We wish to clarify the that the claim pertains specifically to morphological maturation, and we do not observe any disruption in the morphological development of gametocytes in the Δ*Pf*MD3 line. To further investigate this, we have now incorporated single-cell RNA sequencing (scRNA-seq) data from the *Pf*MD3 knockout, which reveals a decrease in the total proportion of male cells within the population. Importantly, cells map along the entire male developmental trajectory, reinforcing our initial observation that fewer male gametocytes are produced but their development is not arrested at any point during maturation (Figure 3C; shown below). However, we do observe a reduction in male gametocyte-associated transcripts within the male lineage (Figure 3D; shown below), suggesting that the morphologically normal male gametocytes are functionally sterile—analogous to inactive or defective spermatocytes—and therefore unable to participate in successful fertilization.

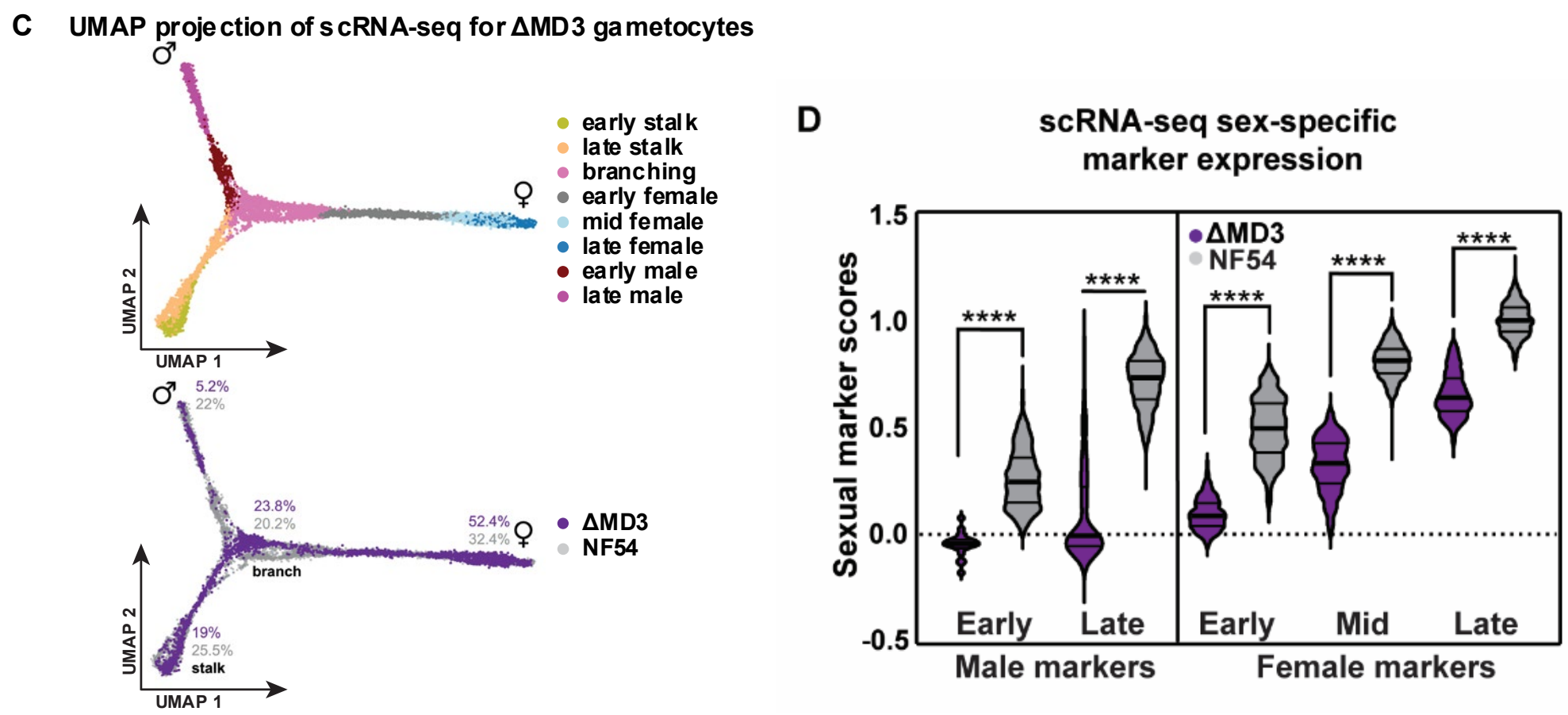

10. Figures 2F, H, I: Statistical Ambiguity, no statistical information (e.g., replicates, error bars, p-values) is provided. The authors should detail the analysis and annotate figures appropriately.

> We thank the reviewer for identifying this omission. The legend for Figure 2 has been amended in the revised manuscript to include all relevant statistical information, including the number of biological replicates and parasite clones used for each experiment, the statistical tests applied, and the corresponding p-values, which are indicated directly on the graphs where differences are significant, as annotated in the figure legend.

11. Lines 195–196: Cross Outcome Unclear, the Δ*Pf*MD3 × ΔMACFET cross yielded normal oocyst numbers, despite ΔMACFET being deficient in gametocyte development. This result needs further explanation.

> We respectfully note that the reviewer's concern regarding the outcome of the Δ*Pf*MD3 × ΔMACFET cross appears to stem from a mischaracterization of the ΔMACFET phenotype. The phenotype of the ΔMACFET line described in the original study (PMID: 35465336) is not a defect in gametocyte development but rather a female-specific defect in fertilization. To confirm that the reduction in oocyst development seen in the Δ*Pf*MD3 parasite line was attributable to a defect male fertility, we performed a complementation cross with the ΔMACFET parasite line, which is deficient in fertile females. This outcome of this cross is consistent with and confirmatory of the Δ*Pf*MD3 male fertility phenotype. To reduce any potential confusion regarding the experimental design and interpretation of this genetic cross, we have modified the relevant text as follows (Lines 185-193, Page 7): "To further characterize the male defect in gametogenesis, we performed a genetic cross in *Anopheles stephensi* mosquitoes between Δ*Pf*MD3$^{1.1}$ and the *P. falciparum* ΔMACFET[63] strain that is deficient in the production of fertile females. As expected, self-crossed ΔMACFET produced no observable oocysts due to the lack of fertile females. However, when Δ*Pf*MD3 was crossed with ΔMACFET the resulting oocyst numbers were increased (24.4 ± 2.4 oocysts per midgut) compared to either Δ*Pf*MD3 or ΔMACFET self-crosses (Figure 2I). These results suggest that, despite the normal morphological development of the Δ*Pf*MD3 gametocytes, disruption of *Pf*MD3 reduces the production of reproductively competent male gametocytes."

12. Lines 210–211: Unexplored Expression Changes, AP2-FG and HDP1 are upregulated in ΔPfMD3 parasites, but the authors do not interpret their relevance. These genes are functionally important in gametocytogenesis and should be discussed.

We agree that the transcriptional changes for *ap2-fg* and *hdp1* in the *ΔPfMD3* is an interesting observation. However, this increase in mRNA abundance was not associated with a similar change at the protein level. To streamline the discussion of our multi-omics results as requested by the editor and Reviewer #1, we have reduced our mention of many testable hypotheses emerging from these data including this observation of these two transcriptional changes.

13. Lines 227–228: qPCR Validation Missing, RNA-seq is based on only two replicates. There is no RT-qPCR validation of key genes. Moreover, Figure 3A and 3B use the same dataset, which is methodologically circular.

It is an accepted standard for transcriptomics to be based on an n = 2 over multiple timepoints, as demonstrated in Figure 3A. Time-series data have the added advantage that changes are assessed relative to other timepoints, and the inclusion of two biological replicates at each timepoint further reinforces the robustness of these comparisons. We also respectfully disagree with the assertion that Fig 3A and Fig 3B as methodologically circular. Figure 3A displays the full set of significantly changing transcripts across the time course, while Figure 3B highlights a biologically relevant subset of particular interest—these represent two distinct and complementary presentations of the same dataset, not a circular analysis. With respect to the request for RT-qPCR validation of key genes, we have now included this validation, with results presented in Figure S4B (shown below) and the corresponding primer list in Table S6.

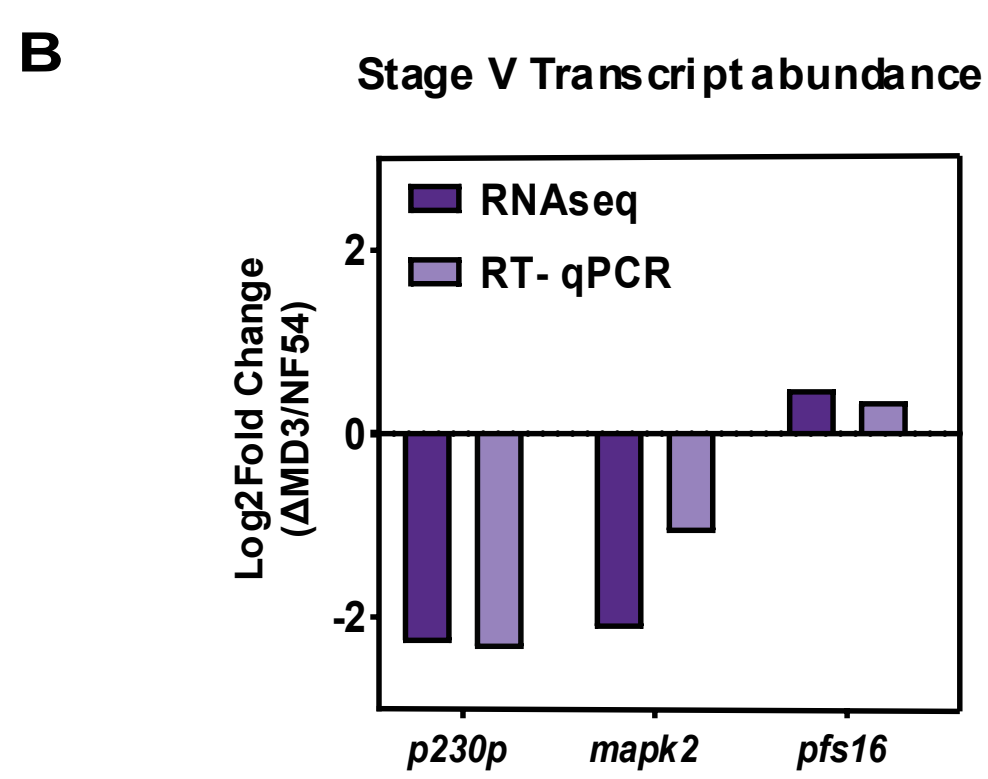

14. Lines 228–230, 250–252: RNA-Protein Discordance, Numerous female gametocyte transcripts (e.g., P47) are unchanged at RNA level but decreased at the protein level. These inconsistencies are not explored or validated.

We appreciate the reviewer's suggestion and agree that experimental validation of these changes would strengthen our conclusions. As such, we have added a Western blot data validating the reduction in both P230 and P47 protein levels in Δ*Pf*MD3, as well as RT-qPCR validation of select transcriptomic changes for male and female gametocyte markers. As shown in Figure 3B, a modest decrease in the female

transcript markers is observed at the RNA level, though this difference only reaches significance at the protein level. Our scRNA-seq data, which resolves male and female populations independently, further corroborates this finding by illustrating a decrease in both male and female transcript markers within their respective populations (Figure 3D; shown below). We propose that the reduction in both male and female markers at the protein level may reflect broader changes in translational dynamics in the absence of *Pf*MD3, a possibility that is explored in greater detail later in the manuscript.

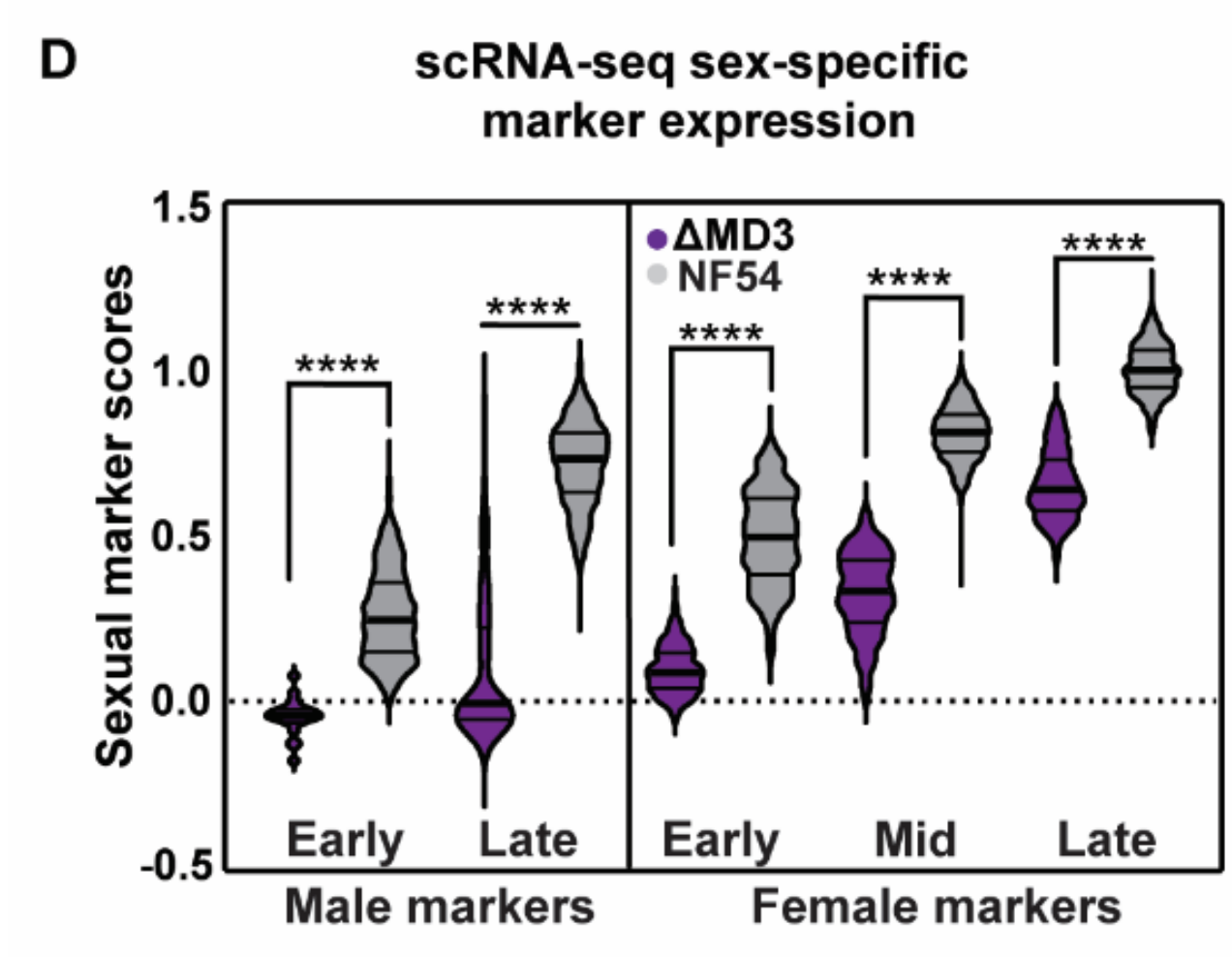

15. Lines 253–261: Timing Claims Not Validated, The authors propose that PfMD3 misregulates the timing of protein expression, but provide no validation by IFA or WB over developmental timepoints.

As part of streamlining the discussion of our multi-omics results, as requested by the editor and Reviewer 1, we have removed this hypothesis from the revised manuscript. While we do observe low levels of certain proteins in early-stage *Pf*MD3 knockout gametocytes that are not detected in early-stage NF54e gametocytes, we acknowledge that there alternative interpretations of these observations exist that cannot be adequately resolved using the available datasets, and we therefore consider it premature to draw firm conclusions regarding the timing of protein expression in the absence of *Pf*MD3.

16. Lines 295–298: RNA-Binding Domain Unclear, PfMD3's RNA-binding domain is not dissected. A CCCH-domain deletion or domain swap could clarify binding specificity.

We respectfully disagree with the suggestion that the RNA-binding domain of *Pf*MD3 is not dissected. We have rigorously characterized the binding specificity of the CCCH-type zinc finger domain using complementary *in vitro* and *in vivo* methods, representing one of the most thorough examinations of a malaria parasite-encoded RNA-binding domains reported to date and the first study in *P. falciparum to* employ three complementary approaches for this purpose. Nevertheless, to further demonstrate sequence-specific RNA-binding by the CCCH-type zinc finger domain, we have added a new experiment in which the structural integrity of the domain is disrupted by EDTA-mediated zinc chelation (described in the Materials and Methods section "RNA Bind-N-Seq and RNA Electrophoretic Mobility Shift *in vitro* binding assays" and shown in Figure 4F; added below). When the CCCH-type domain was incubated with

the zinc chelator, sequence-specific RNA-binding was abolished as assessed by RBNS, consistent with a loss of secondary structure in the RNA-binding domain in the absence of zinc.

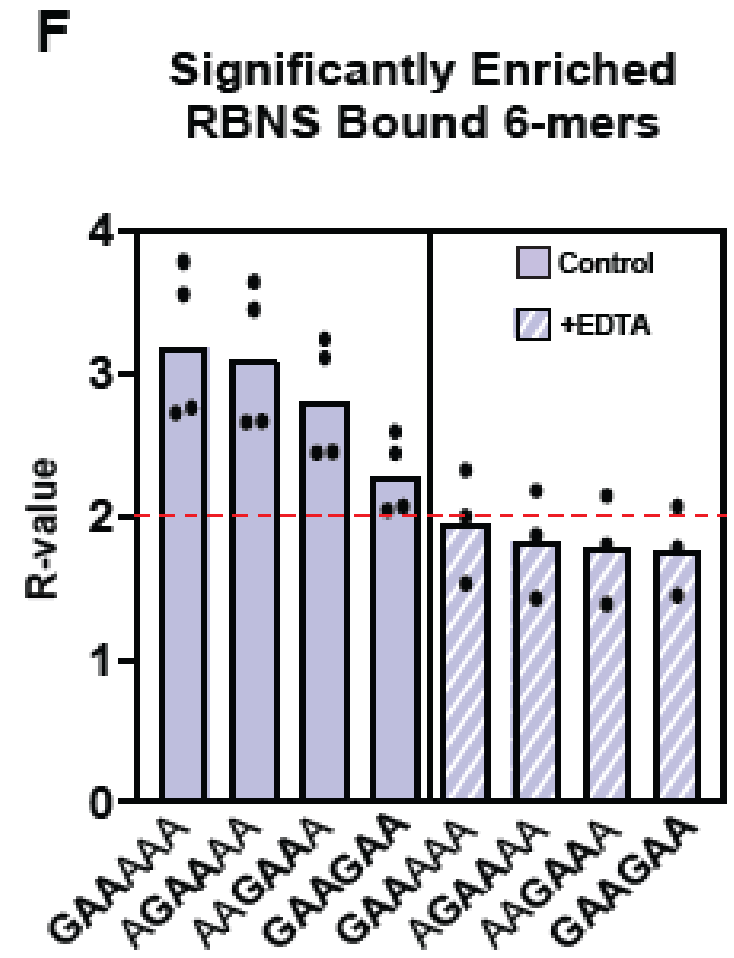

17. Lines 323–326: Weak Motif Enrichment, The GAAGAA motif is common across the genome, with modest enrichment (p = 3.2e-4 to 6.7e-6). EMSA results show similar binding to scrambled probes and no clear competition. These data do not convincingly demonstrate specific RNA binding.

We agree that the GAAGAA motif is common across the genome but disagree that this diminishes the likelihood of a sequence-specific interaction. Genome-wide sequence recognition remains a major question in the area of transcriptional regulation since it has been widely recognized that sequence motifs are far more abundant than the actual binding sites used by any given RNA- or DNA-binding protein. In fact, we have published on this topic previously (PMID: 38966997).

With respect to the EMSA data presented in Figure 4, the probe was generated from the coding sequence of *p230*, which contains three GAA repeats. The "scrambled" probe represents the full sequence of the *p230* probe that was randomized using the "Shuffle DNA" tool from bioinformatics.org but still retained one instance of the GAA trinucleotide. The competition observed is therefore less complete than would be expected from a probe in which all GAA instances are eliminated. To more convincingly demonstrate binding specificity, we have included an additional EMSA using a probe derived from the same *p230* sequence in which all GAA instances were removed by point mutations (Figure 4G; shown below). The complete loss of a gel shift with this GAA-depleted probe demonstrates that *Pf*MD3 interacts directly with the GAA motif within the *p230* sequence and supports our assertion that the *Pf*MD3 CCCH-type zinc finger domain binds RNA in a sequence-specific manner. This new data has been incorporated into Figure 4 and is described in the results as follows (Lines 349-357, Page 12-13): "Using a biotin-labeled RNA probe designed from the *p230* coding sequence containing multiple GAA-repeats, we observed robust binding by the recombinant *Pf*MD3 CCCH-type ZnF domain that could not be outcompeted by a scrambled control probe at any concentration tested (Figure 4G). Interestingly, we also observed a supershift in the *p230* RNA probe, which may indicate multimerization of the CCCH-type zinc finger. We also performed EMSAs with a scrambled *p230* RNA probe retaining a single GAA trinucleotide and a mutant probe with

all GAA instances replaced by CUU (Figure 4G). While the *p230* scrambled probe was weakly bound, the GAA-to-CUU mutant probe showed no detectable binding."

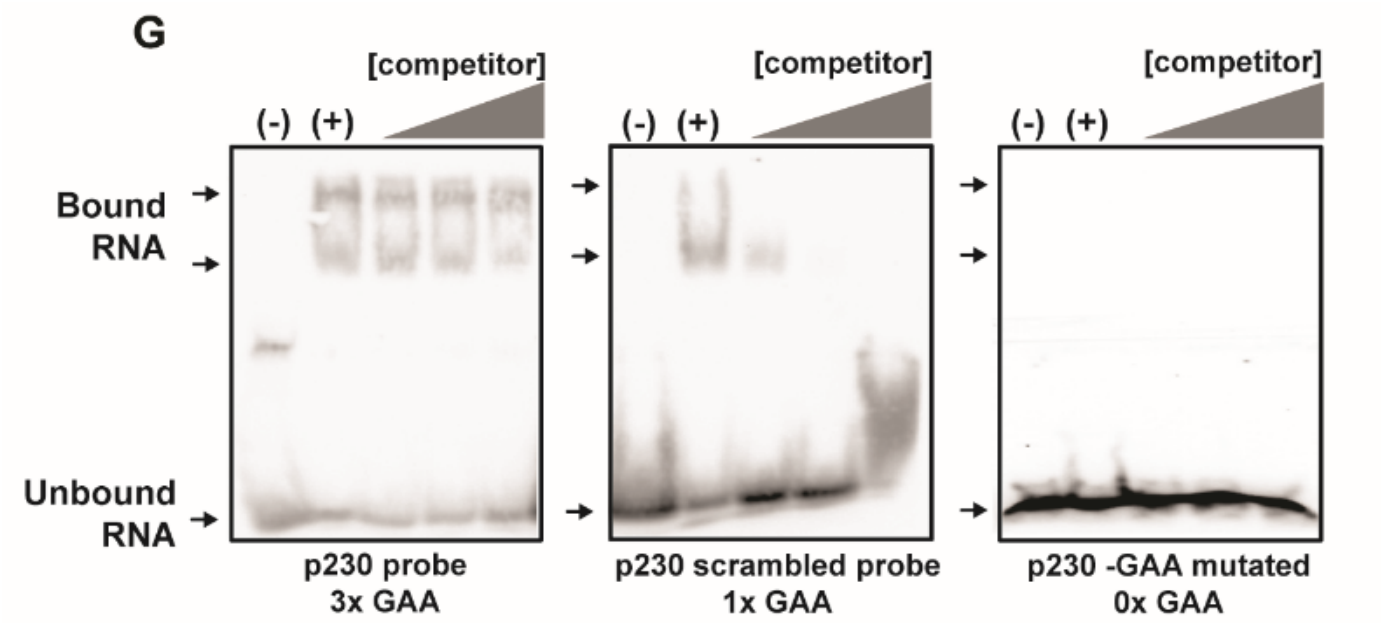

18. Supplementary Figure S5: Confused Labeling, Legends for S5A and S5B are mismatched. The motifs differ between CLIP-seq and RNA-bind-n-seq, and no p-values are shown for the latter.

We thank the reviewer for identifying the error in the legend for Figure S5. In the revised manuscript, we have repeated the RNA Bind-N-Seq (RBNS) experiments to a sequencing depth that is in accordance with ENCODE guidelines for RBNS requirements. We have replaced Figure S5A and the RBNS results are presented as part of Figures 4E and 4F below.

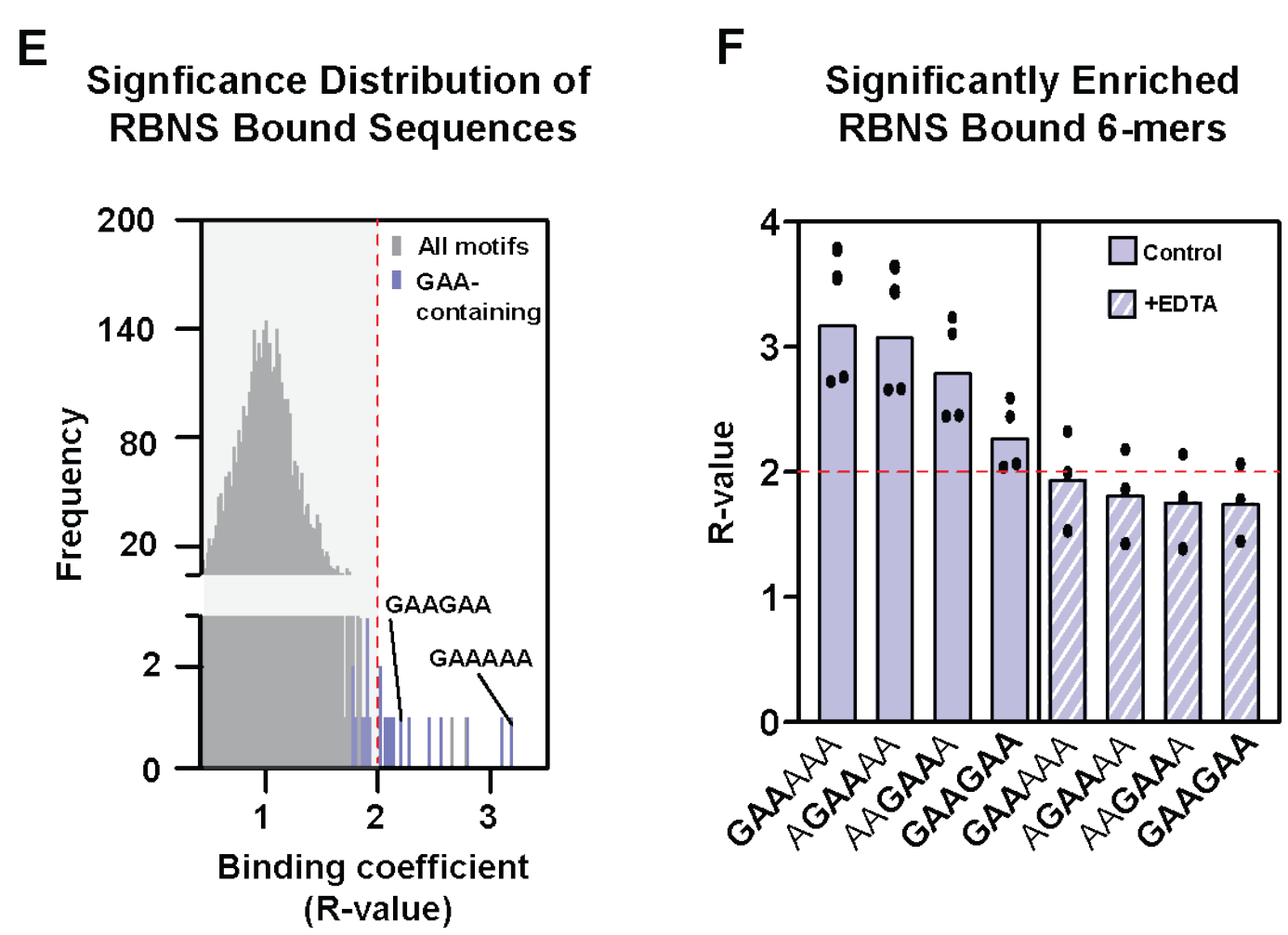

With respect to the significance statistics for the CLIP-seq and RBNS, we note that while we can calculate a P-value for the CLIP-seq results, the appropriate significance statistic for the RBNS output is the "R-value". As described in the original published method (PMID: 24837674), the R-value quantifies the enrichment of a specific k-mer relative to an input RNA library. Per ENCODE guidelines, the threshold for significance in RBNS is defined as "at least one 6-mer should show enrichment (R) value >2 in at least one concentration". We note that *P*-values and R-values are not directly comparable metrics, and we do not claim that the CLIP-seq and RBNS results are identical. Rather, we propose that a core "GAA" trinucleotide sequence is sufficient for *Pf*MD3 binding *in vitro*, while *in vivo* binding may be further influenced by the number of GAA repeats present within a given target sequence.

19. Lines 335–337: Overinterpretation, The authors overstate conclusions based on modest statistical enrichment and weak binding validation. Statements should be revised for caution and accuracy.

We strongly disagree with the reviewer's assessment that our conclusions are overstated. Nevertheless, to more precisely communicate our findings and ensure that the language accurately reflects the strength of the supporting evidence, we have revised the relevant text to be more specific with respect to sequence specificity, in line with the conclusions drawn from our additional experimental results. The revised statement, presented in Lines 357-360, Page 13, reads as follows: "We conclude that *Pf*MD3 binds a GAA-triplet motif via the CCCH-type ZnF domain and that these trinucleotide sequences often occur in repeated clusters within RNA transcripts bound by *Pf*MD3."

20. Lines 345–352: Binding vs. Function, Of 207 CLIP targets, only 66 show protein changes. If PfMD3 regulates translation, more overlap would be expected. The role of the remaining bound RNAs is not explained.

We understand the reviewer's concern that the overlap between CLIP-seq targets and detectable changes in protein levels may not appear substantial at first glance. However, when considered in the context of what can be detected from whole-cell proteomics, the overlap is in fact significant. Of the 3,031 proteins detected in our proteomics dataset, 170 are products of CLIP-seq target transcripts. Of these 170 CLIP-seq targets, 25% are significantly decreased in protein abundance in *ΔPf*MD3 parasites relative to the wild-type (with 31% showing any decrease). This represents a substantial over-representation of protein-level changes occurring in CLIP-seq targets, given that these targets comprise only 5.6% of the detected proteome, and this enrichment is statistically significant ($P<0.0001$, Fisher's exact test). We further note that published studies on both transcriptional (PMIDs: 26067602, 26417110, and 33368818) and translational regulators (PMIDs: 38702310, 25418785, and 31737630) generally report similar or lower overlap between directly bound targets and downstream transcript or protein abundance changes than that observed here for *Pf*MD3.

With respect to the remaining bound transcripts that do not show detectable protein-level changes, we acknowledge that *Pf*MD3 binding alone may not be sufficient to determine translational outcome. Additional layers of post-transcriptional regulation may supersede or modulate the effect of *Pf*MD3 binding for a subset of target transcripts, and fully resolving these interactions is beyond the scope of the present study.

21. Figure 6B and IP-MS Data : Poor Replicate Consistency -- Only four proteins overlap between IP-MS and CLIP-seq. Replicates vary greatly, and stages (S II-III) used for IP differ from other datasets. A no-tag control strain should be used. Known interactors (e.g., ZNF4, RNF1) were already validated in prior studies.

Below we respond to each of these points individually regarding Figure 6B and IP-MS Data.

- **Overlap between IP-MS and CLIP-seq -** While it is not expected that an RNA-binding protein would interact with both the transcript and the protein product of that same transcript, we noted that 9 out

of the 102 proteins significantly enriched in the *Pf*MD3 IP-MS, were also encoded by transcripts bound by *Pf*MD3 in the CLIP-seq dataset. We felt that this was an interesting observation and stated that "we found some overlap (9 proteins) between the *Pf*MD3 mRNA CLIP targets and protein interactors (Figure 6B, Table S5)." As we do not have a clear functional hypothesis to explain this co-occurrence, we have removed this statement from the revised manuscript to avoid overinterpretation.

- **Replicate consistency -** The proteins co-immunoprecipitate with *Pf*MD3 were identified by DIA-MS across three biological replicates of Stage II gametocytes (Day 5), each processed and run independently. To ensure that we reported on high confidence interactions, we applied a stringent criterion requiring enrichment to be consistent across all three replicates (Table S5, as described in the text).
- **No-tag control -** A standard and widely accepted control for non-specific antibody-mediated interactions in an IP-MS experiments is an IgG pulldown (PMID: 35300004). Based on common protocols, we included a rat-IgG control to identify any interactions that were a result of non-specific binding to the anti-rat-HA antibody used to immunoprecipitate tagged *Pf*MD3. A no-tag control strain is not a necessary control for this experiment.
- **Previously validated interactors -** We agree that a handful of the identified interactors have been validated in prior studies, and these are described and appropriately cited in the text on page 15 and line 421-424, "Two interactors, both zinc finger domain containing proteins (ZNF4: PF3D7_1134600 and RNF1: PF3D7_0314700), were previously identified and verified via reciprocal immunoprecipitation[72], providing independent validation of our IP-MS approach (Figure 6B)." In this study, we have substantially expanded the list of potential *Pf*MD3 interactors. To provide the reviewer with confidence in our results we have confirmed several of the newly identified interacting proteins via pulldown and Western Blot analysis (Figure S6C and S6D). We also demonstrate that *Pf*MD3 interacts with ribosomes using polysome profiling as suggested by some of the top hits from the IP-MS (Fig 6C). These results are shown below and are described in more detail on pages 15-16, lines 427-433 and 449-452.

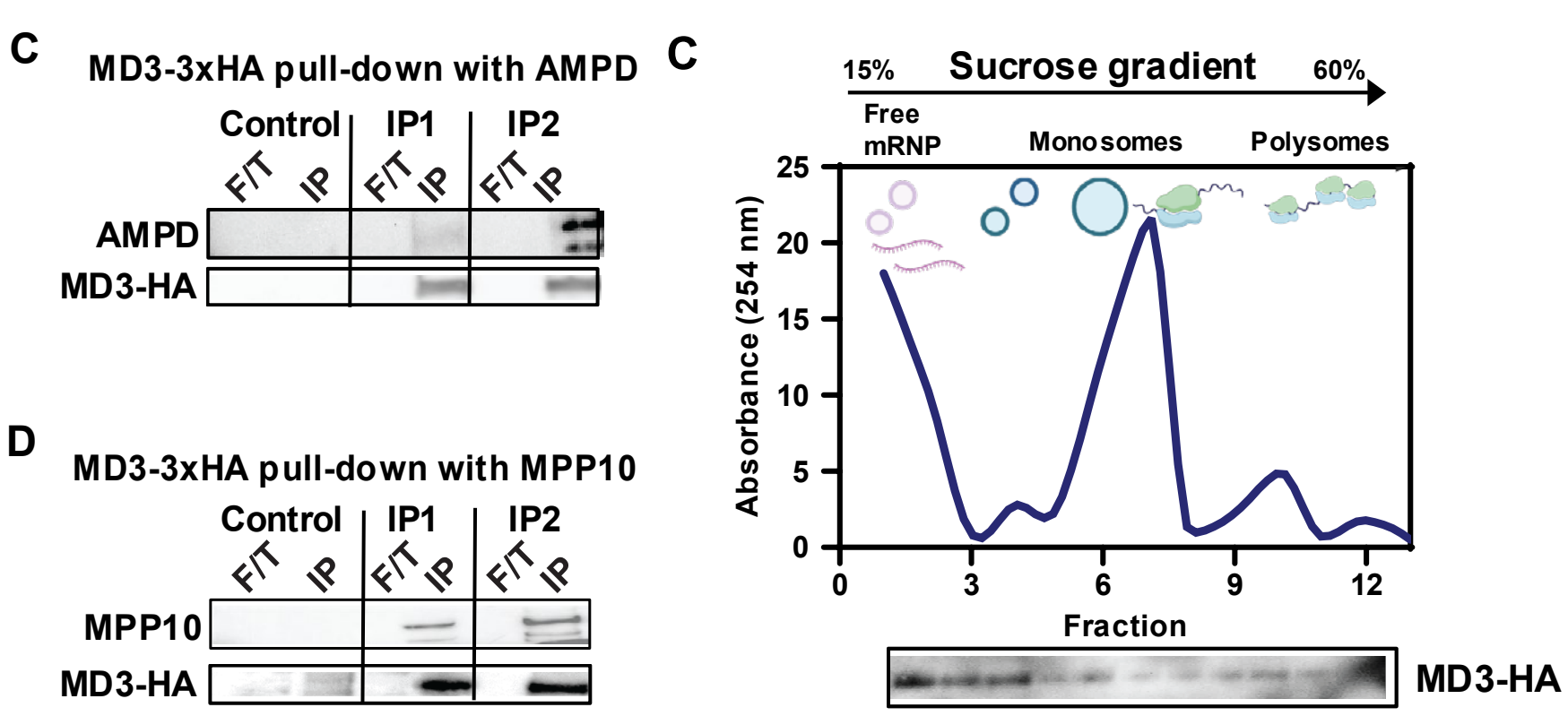

22. Line 507–509: Core Conclusion Contradicted, The authors admit that direct targets of PfMD3 are unknown, despite extensive multi-omics profiling. The overlap between datasets is minimal. As a result, the central claim

of PfMD3 as a sequence-specific translational activator remains unproven, and the novelty of the study is diminished.

We find that the reviewer misunderstood our conclusion where we stated, "the exact mechanisms and purpose of a single protein presenting with such diverse functions remains unknown." This does not imply that the direct targets of *Pf*MD3 are unknown. Rather, it acknowledges that it is not yet fully understood how a relatively simple protein is able to fulfil the complex and multifaceted functions revealed by our data. We clearly demonstrate that the CCCH-type zinc finger domain of *Pf*MD3 interacts with specific mRNA in a sequence-specific manner (Figures 4, S5 and S6) and provide evidence supporting a role for *Pf*MD3 as translational activator. This proposed function is supported by the combined multi-omics analysis of the *Pf*MD3 knockout strain (Figures 3 and S4) as well as the protein-protein interactions identified using the *Pf*MD3-HA tagged strain (Figure 6). We therefore disagree that the novelty of the study is diminished—all of the data presented in the figures referenced above are novel to this study and collectively serve to extend and clarify the molecular function of *Pf*MD3 during gametocytogenesis.

Reviewer #4 (Remarks to the Author):

This manuscript presents a dissection of the role of Md3 in male gametocytes. It is a careful and considered study and is an important addition to the knowledge on RNA biology in male gametocytes which is currently being studied by several groups. The study fills an important gap in understanding male-specific translational control during gametocytogenesis. The data is of good quality and the conclusions are in majority supported throughout the manuscript. I have a number of small and a few more substantial comments to improve the manuscript:

We appreciate this reviewer's thoughtful and positive assessment of our findings. We hope that our direct responses to your comments and the inclusion of additional experiments serve to further improve the manuscript.

Comments:

Introduction
-It's unclear why the introduction details sexual commitment to this extent as it's not central to the message of the paper (also oddly repeated in the discussion).

We have revised the Introduction and Discussion sections of the manuscript to be more focused on the information necessary to interpret the central message of the paper.

-It is not known how long gametocytogenesis takes in all Laverania species (also in the discussion)

We thank the reviewer for pointing out our over-generalization. In the revised manuscript, we have removed the reference to length of gametocytogenesis in *Laverania* species.

Fig 1

-It is odd that there is no detection of the protein in the asexuals whilst the immunofluorescent image of schizont is compelling.

We acknowledge that the absence of *Pf*MD3 detection in asexual parasites via western blot, while expression is clearly evident by IFA, would be inconsistent. The original western blots presented in the manuscript used equal concentrations of lysate per sample. A longer exposure revealed expression in trophozoites and schizonts; however, this was not included in the original manuscript due to high background signal (now shown in middle blot below and added to Figure S1C). We also note that only approximately 30% of asexual stage parasites expressed *Pf*MD3-3xHA as detected by IFA, which may contribute to the relatively weak signal observed by western blot under standard loading conditions. For this resubmission, we repeated the western blot using increased amounts of asexual stage cell lysate (Figure S1C, rightmost blot). This new blot clearly demonstrates *Pf*MD3 protein expression throughout the asexual intraerythrocytic development cycle (Figure 1D).

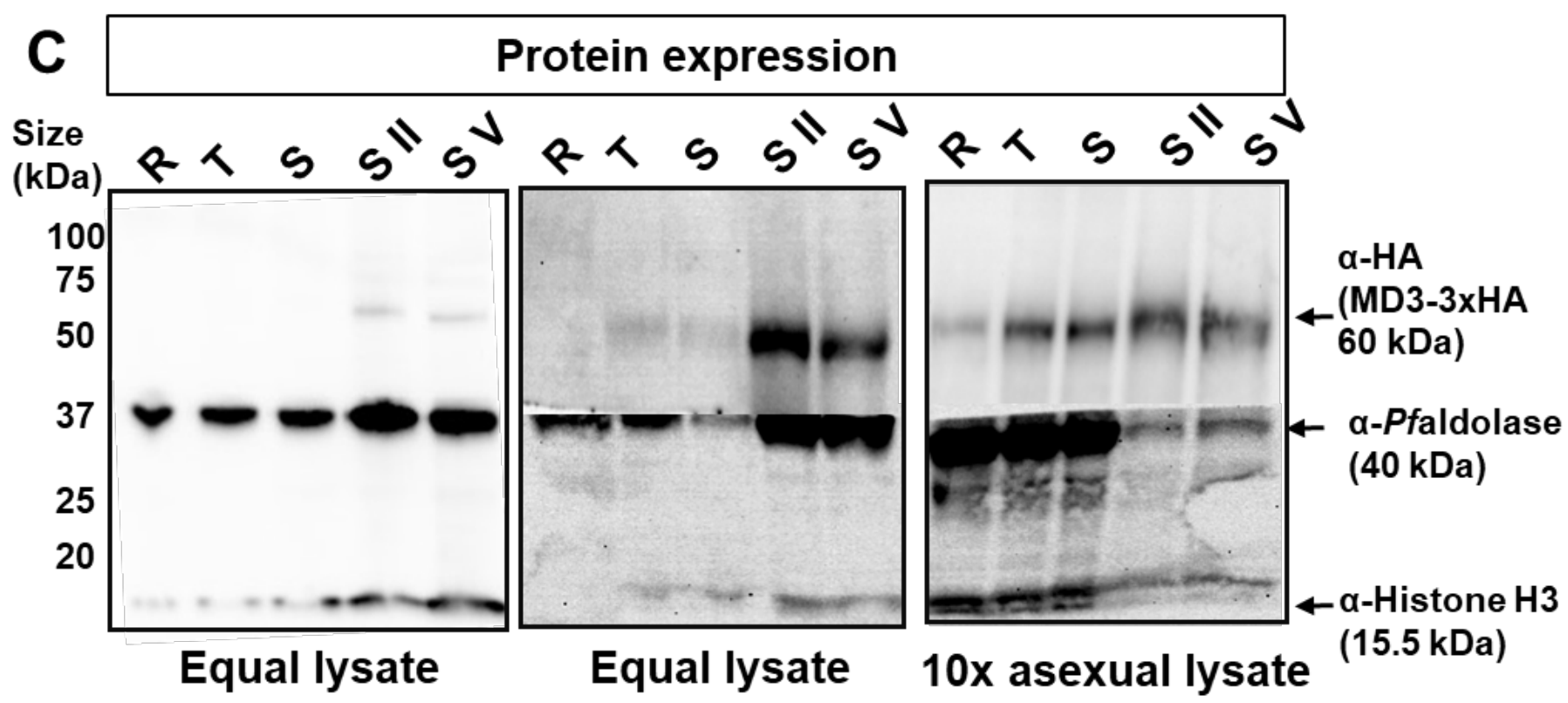

Fig 2

-The enhanced asexual multiplication rate is very interesting and potentially important in defining the md3 functon (this was not detected in a genome wide screen in Pb). The author imply that this may be due to the lesser sexual commitment of these clones but this is not tested. The authors could culture these parasites with lysoPC or choline to establish an intrinsic differential growth rate.

We agree with the reviewer that this is an interesting finding and appreciate the encouragement to further examine the asexual multiplication rate. Based on your suggestion, we have added new results from a growth comparison of NF54e and the Δ*Pf*MD3 parasites cultured in the presence or absence of choline. We find that no difference in asexual replication between the two strains when sexual conversion is blocked by choline treatment (Figure S2F; shown below), confirming that the enhanced replication rate observed in the Δ*Pf*MD3 knockout strain is due to a reduction in sexual conversion, likely driven by the decrease in male gametocyte production in this parasite line.

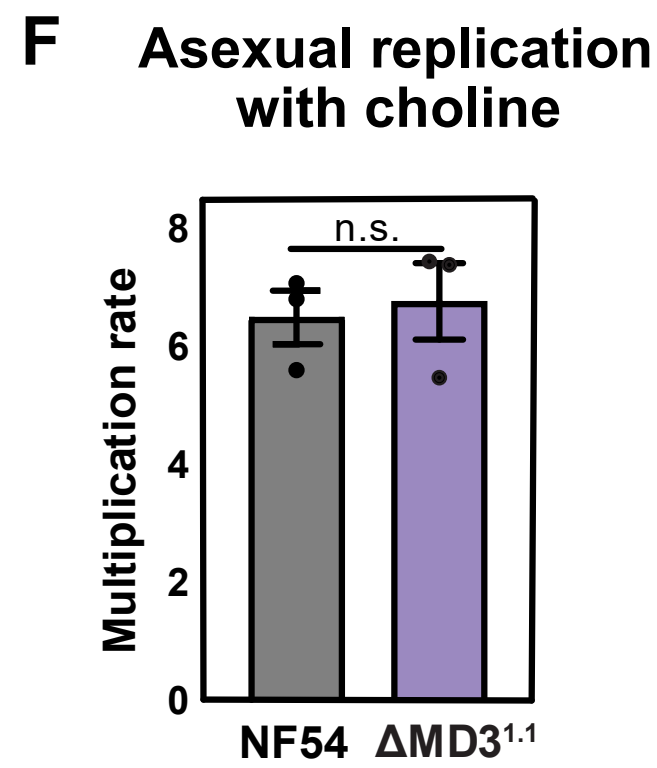

-Scoring sex ratios by Giemsa is difficult and often inaccurate especially when mutants may display subtle morphological changes, since the authors use the p230 antibody, it is not clear why they have not used that to accurately quantify the sex ratio. This is an important conclusion to validate.

> We agree that scoring sex ratios by Giemsa staining is technically challenging, requires an experienced eye, and may be influenced by subtle morphological differences that are unique to specific mutant lines. To address this concern, we have now included sex-scoring results comparing wild-type and Δ*Pf*MD3 parasite lines using immunostaining with either a P230p antibody to identify male gametocytes or a P47 antibody to identify female gametocytes (Figure 2E). These results clearly demonstrate a significant reduction in the number of P230p-positive male gametocytes in the Δ*Pf*MD3 line, providing a robust validation of this key conclusion.

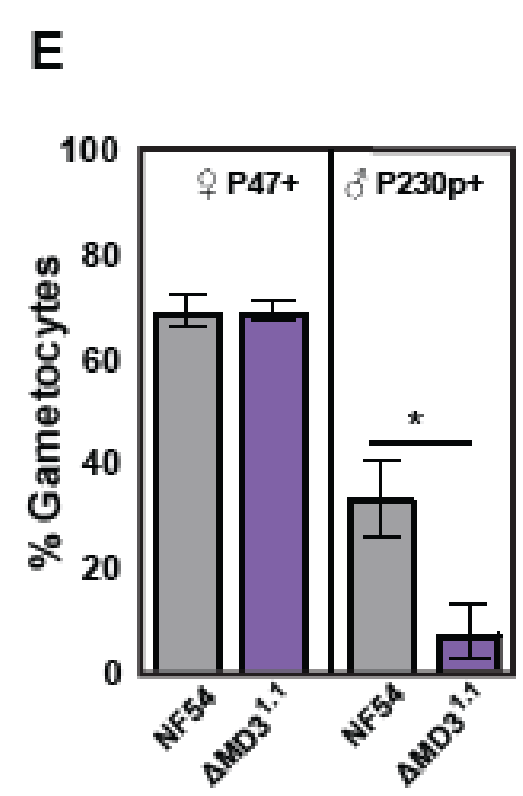

-Commitment is sensitive to cloning out parasites, to my mind it is not sufficient to have reduced commitment in two clones to claim that commitment is affected by the Md3 disruption in the absence of complementation studies. I am not suggesting that this is needed for the paper but the claim is not supported.

> We agree with the reviewer's concern and have now include additional data to support our claim by generating and characterizing a *Pf*MD3 conditional knockdown strain. This new strain uses the *glmS* ribozyme system (PMID: 24023691) to reduce the expression of *Pf*MD3 in the presence of glucosamine. Similarly, removal of glucosamine should restore *Pf*MD3 protein expression, in effect replacing a traditional full genetic complementation study. We first established that the glmS ribozyme is integrated into the genome at the 3' end of *Pf*MD3 and functional (Figures S3A and B; shown below). We then determined that the addition of 1.25 mM glucosamine is sufficient to knockdown expression of *Pf*MD3

in clone 7 (Figure S3C; shown below) and show that this reduction is sufficient to cause a reduction in sexual commitment (Figure S3D; shown below). This result confirms the phenotype we first presented with the *Pf*MD3 knockout strain (Figure 2C). Additionally, using this *Pf*MD3 knockdown strain we were also able to recapitulate the increase in asexual replication (Figure S3D; shown below) and reduction in exflagellation (Figure S3E; shown below) in the presence of glucosamine similar to the *Pf*MD3 knockout phenotype (Figures 2B and F). By comparing growth phenotypes with or without glucosamine treatment on the same genetically modified strain should erase the reviewer's concern that the reduction in conversion is an artifact of clonal selection. These results are now discussed in lines 153-175 of the revised mansucript.

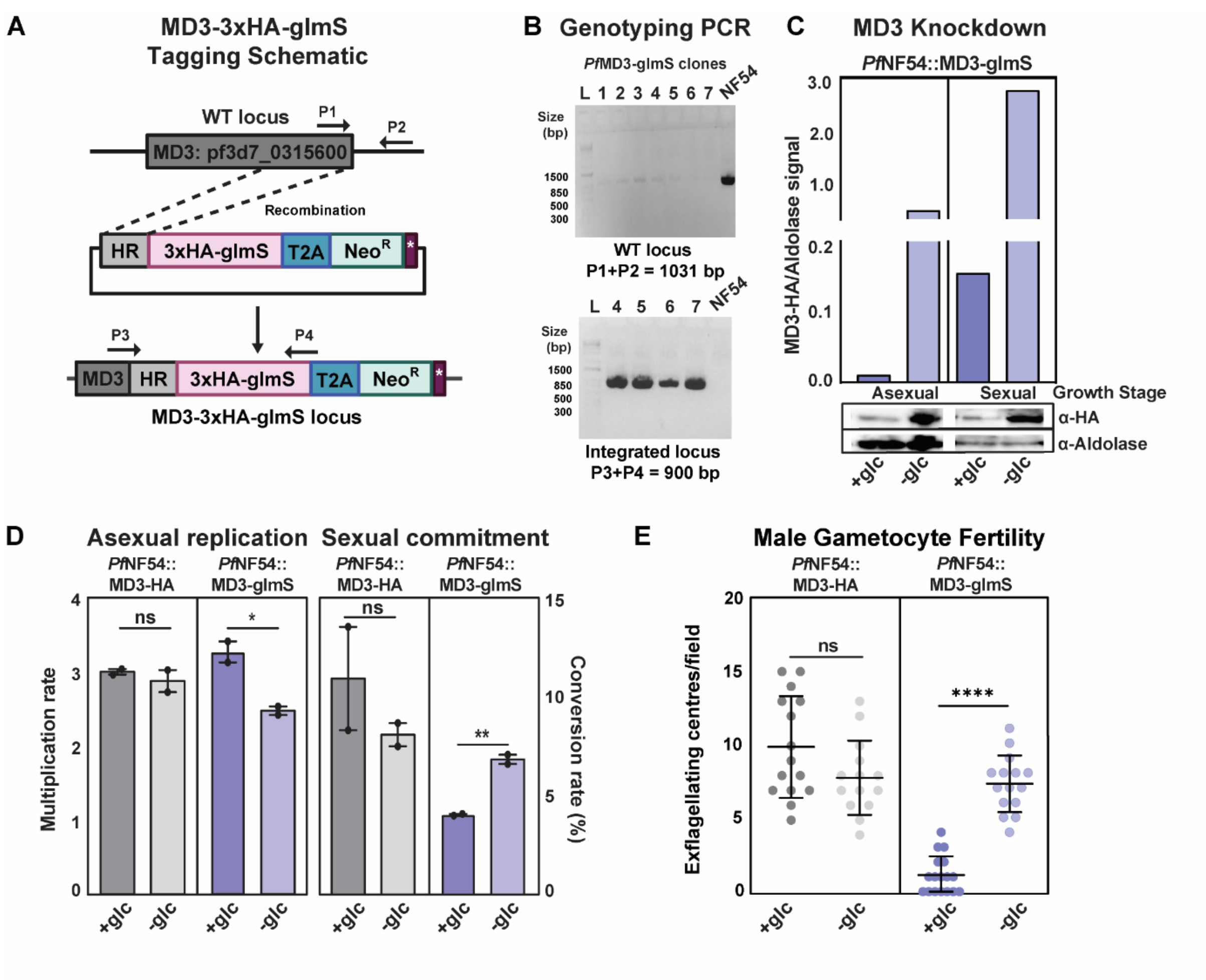

-The statement portraying an absence of maturation defect based on simple morphology is also too hasty, giemsa-morphology is very crude measure of proper maturation. Especially given the much more granular understanding that the authors go on to describe

We agree that Giemsa-based morphological assessment is a relatively crude measure of gametocyte maturation. To address this limitation, we have incorporated a more rigorous assessment of the sexual population in both the Δ*Pf*MD3 and wild-type lines using immunostaining with both a male-specific antibody (anti-P230p) and female-specific antibody (anti-P47) as alternative methods for determining

gametocyte sex (Figure 2E, included earlier in the document on page 32). Using this approach, we conclude that the loss of *Pf*MD3 results in a significant reduction in mature male gametocytes (Figure 2E). This finding is further corroborated by scRNA-seq data, which confirms that the relative population of male gametocytes is reduced within the gametocyte culture (Figure 3C; shown below). In light of these result, we have revised our statement on maturation and updated the interpretation of these findings throughout the manuscript accordingly.

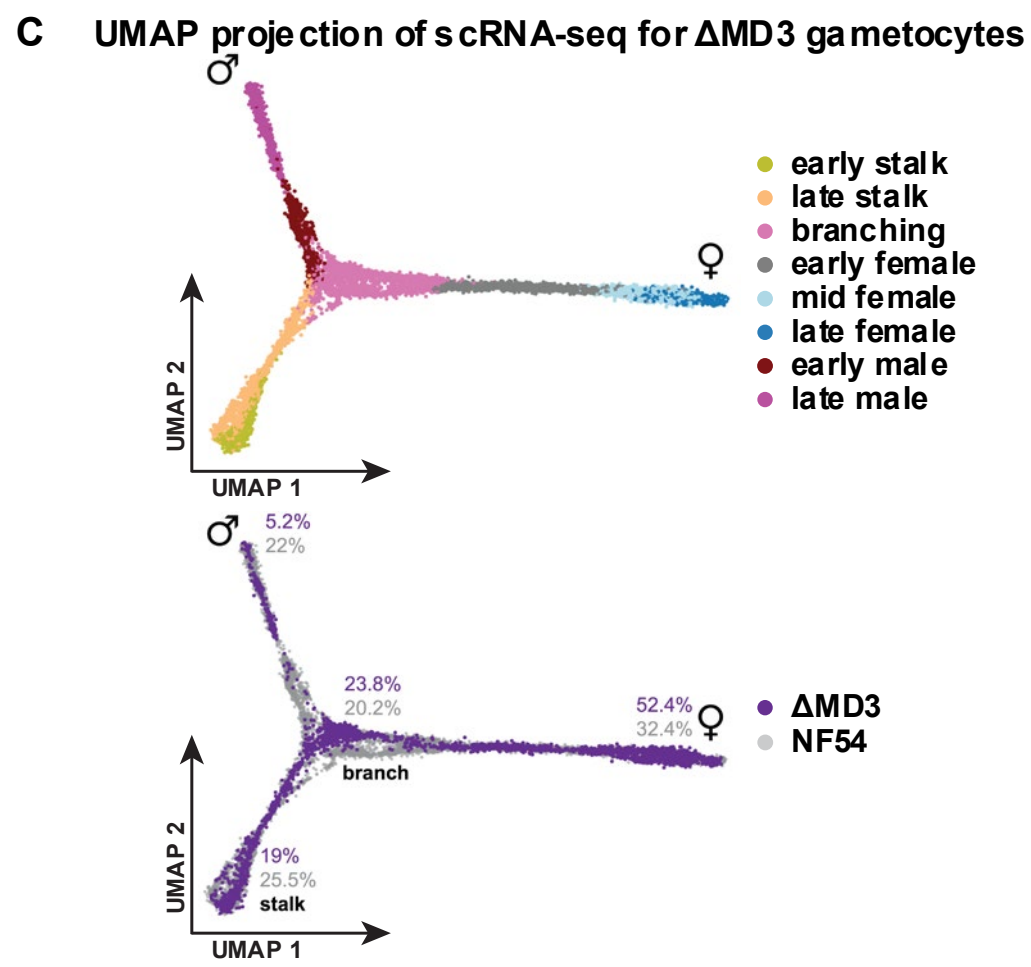

-Exflagellation rate should be corrected for the gametocytemia of the cultures since the overall gametocyte population is reduced.

We agree with the reviewer that the exflagellation rate should be evaluated in the context of the total gametocytemia within a culture. To correct for any differences in the overall gametocyte density between strains, we first adjusted each culture to 0.5% gametocytemia prior to induction and quantification of exflagellating males. This normalization procedure was described in the Methods section as follows: "To assess the ability of each parasite line to exflagellate, mature gametocyte cultures at 0.5% gametocytemia (day 15 post-induction) were resuspended in 10% human serum and incubated at room temperature for 15 min as previously described." (Page 26 and Line 749-751)

- A FACs plot example should accompany the ploidy measurement for the nuclear division in the males

We have amended Figure S2 to now include representative FACS plot examples (Figure S2H; shown below) for the ploidy measurement as requested.

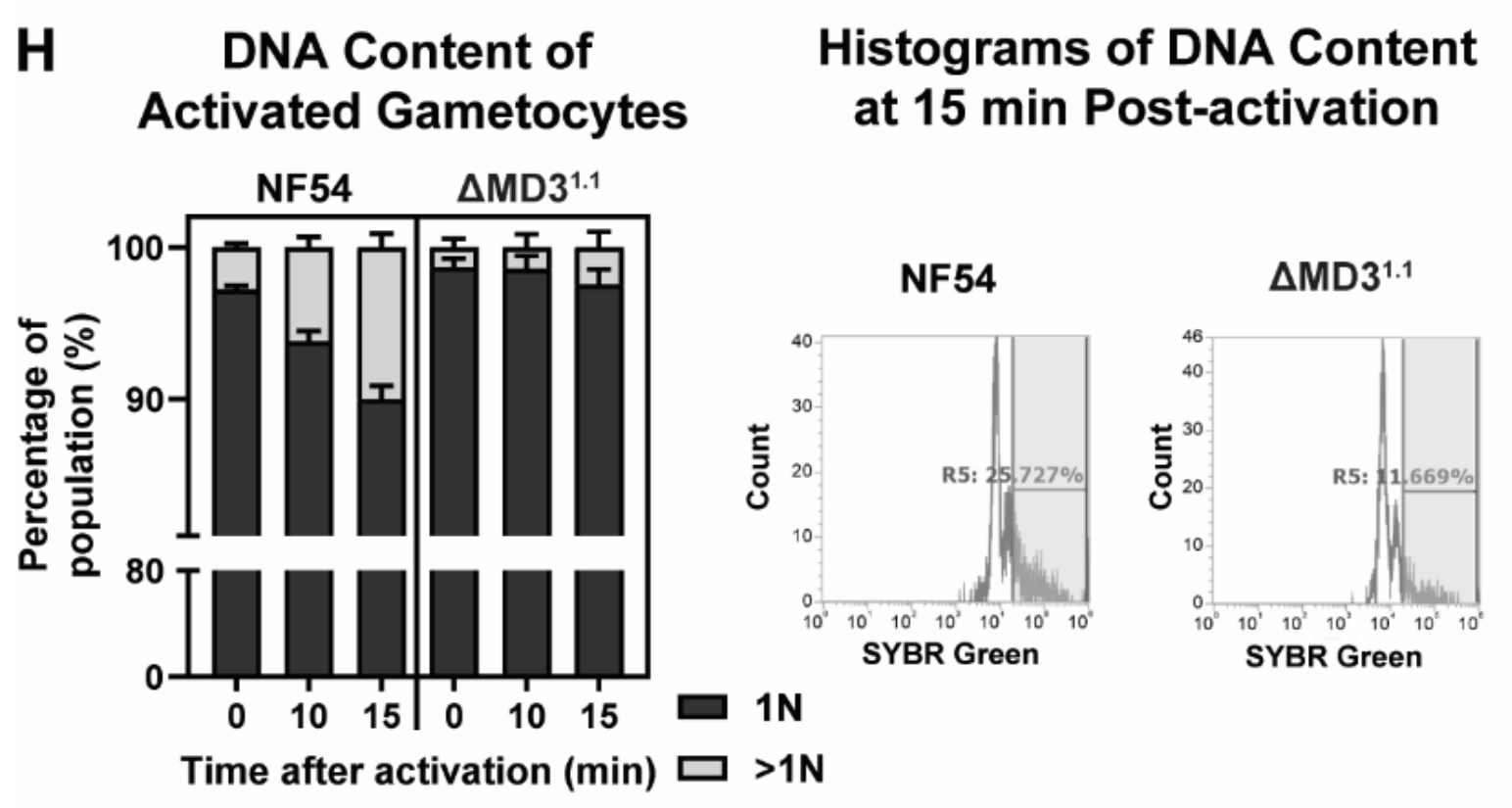

-For the crossing experiment, the data is compelling but I would rather present it as the heterologous cross shows enhanced fertilisation in relation to the homologous one as the normal range statement is not compared to any control…

> We have edited the text to reflect this point. It now reads as, "However, when Δ*Pf*MD3 was crossed with ΔMACFET the resulting oocyst numbers were increased (24.4 ± 2.4) compared to either Δ*Pf*MD3 or ΔMACFET self-crosses (Figure 2I)." (Page 7 and Line 189-191)

Fig 3

-line 243- mitosis not meiosis

> We thank the reviewer for noticing this error. In the process of editing the manuscript based on the comments from another reviewer as well as a request from the editor, we removed this sentence from the discussion of our multi-omics results.

Fig4

For the occurrence of clusters of motifs within MD3-peaks, an IGV example is shown but no global analysis or metric/stat is given.

> To provide a more global characterization of motif occurrence within PfMD3-associated CLIP-seq peaks, we have performed a comprehensive transcriptome-wide analysis of the occurrence of both the AWGAAGAA consensus sequence (Figure S5C; shown below) and GAA trinucleotide motifs (Figure S5D; shown below) within PfMD3 peaks. As noted in our response to an earlier comment, closer examination revealed that the AWGAAGAA motif occurred in repeated clusters within 66 of the 207 enriched regions identified by CLIP-seq as bound by PfMD3 (Figures 4D and S5C), suggesting that repeated AWGAAGAA sequences may be preferentially recognized over single GAA trinucleotide occurrences. These results are now included in the revised manuscript and discussed in lines 327–330 and 340–342 on page 12.

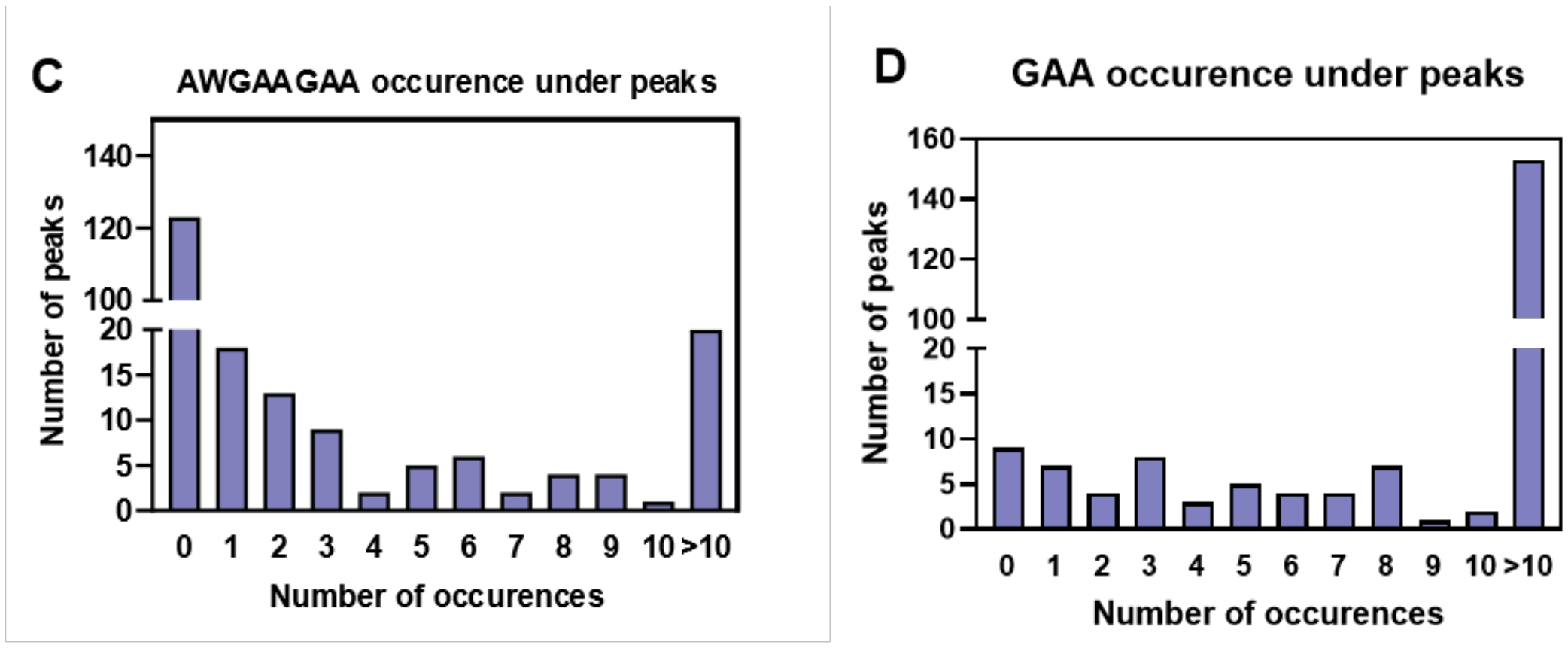

-its unclear why the EMSA probe has a reduced motif (GAAGAA) rather than the full one.

The confusion appears to stem from the labeling in the original Figure. To clarify, the full EMSA probe is 18 nucleotides in length and corresponds to the region of *p230* identified as bound by *Pf*MD3 via CLIP-seq (Figure 4D). While the probe was originally labeled as "GAAGAA-containing", we have amended the label to "*p230* Probe" in the revised Figure 4 (now Figure 4G; shown below) to more accurately convey that the probe sequence corresponds directly to the *p230* region bound by *Pf*MD3.

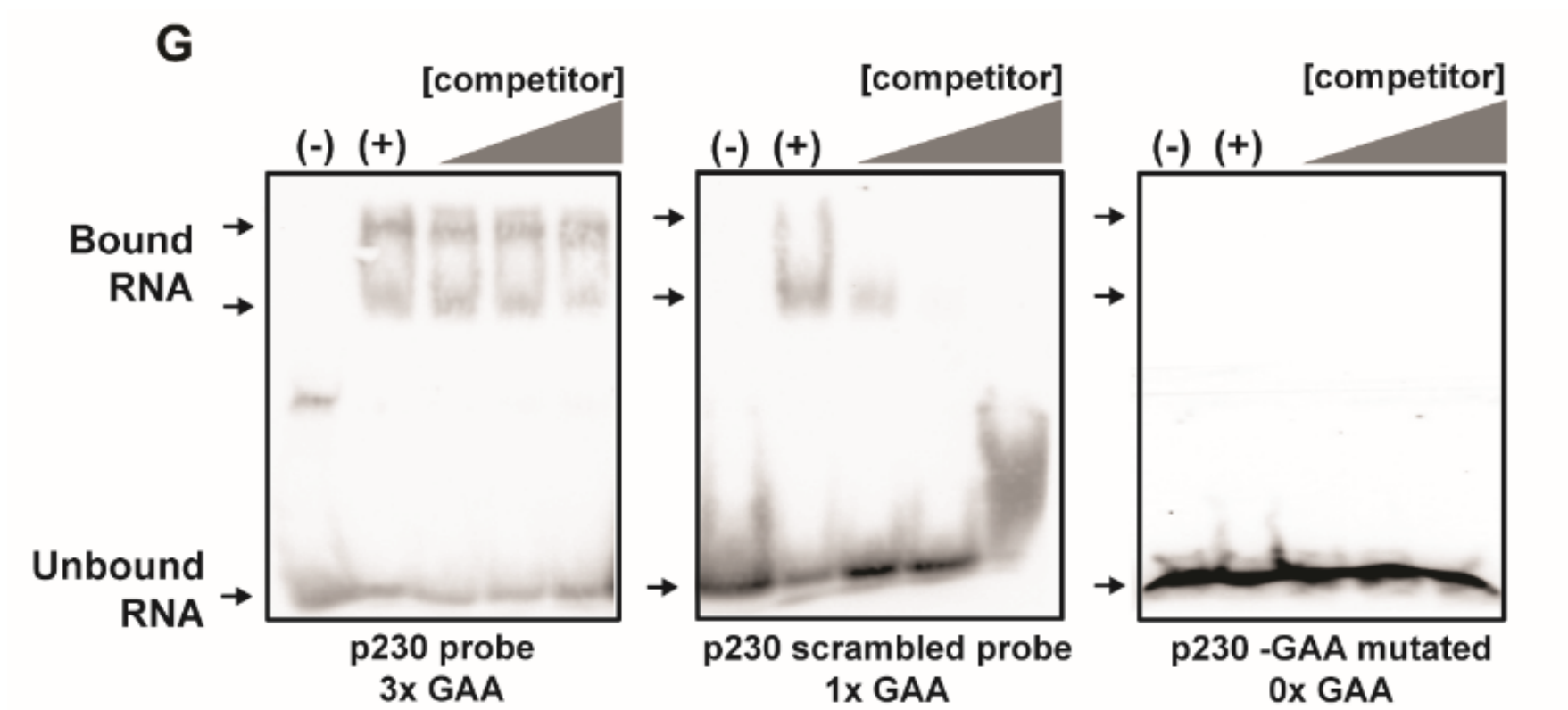

-For the RBNS, is an enrichment of GAA conclusive enough?

To ensure the robustness and significance of this result, we repeated the RBNS experiment in accordance with ENCODE guidelines for sequencing depth and number of concentrations tested, and included both a positive control (a known RNA-binding protein with characterized sequence specificity) and an input control in each experiment. Analysis of enriched sequences identified significant enrichment—defined as an R-value >2 per ENCODE guidelines—of sequences containing a common GAA core motif (Figures 4E and 4F; shown below). As described in our response to an earlier comment, the R-value is the appropriate significance metric for RBNS analysis (PMID: 24837674) and is not directly comparable to a p-value. These results are further supported by our new EMSA data, which confirm that a single GAA trinucleotide occurrence is sufficient for sequence-specific RNA binding by PfMD3 (Figure 4G).

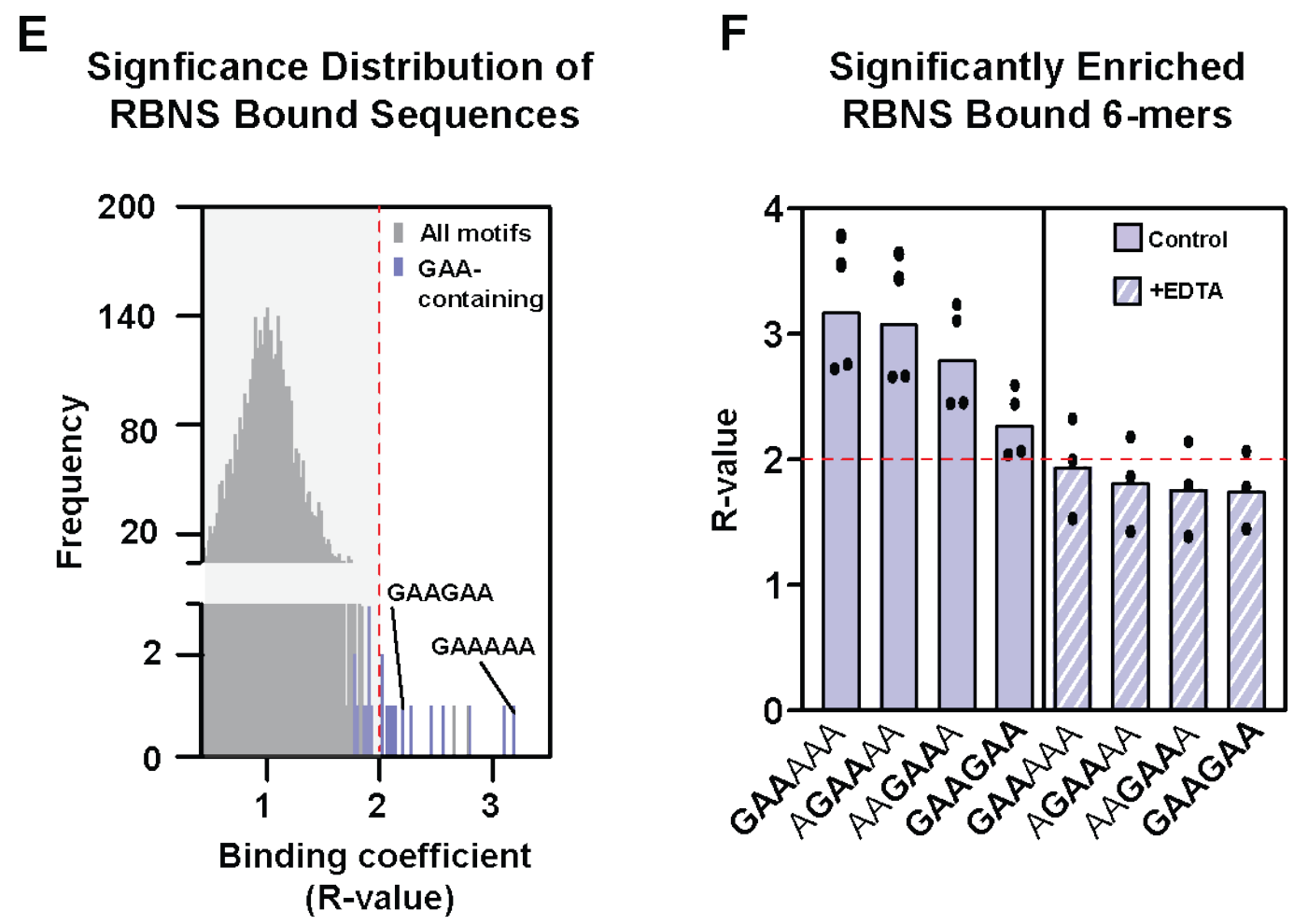

-Line 335 Fig S5A not B. legend of S5A and B mixed up

We thank the reviewer for pointing out this oversight and we have amended the text of this legend to refer to the correct figure panels.

Fig 5

The integration of the data brings the paper nicely together at this stage.

We thank the reviewer for this positive comment.

Fig 6

These data are interesting and the IP is interesting, but none of the identifications are validated by another method so some of the conclusions are quite bold in this part and could be transferred to the discussion.

We agree with all the reviewers that additional supporting evidence was necessary to further support the conclusions drawn from the *Pf*MD3 protein-protein interaction data. In our revised manuscript, we have included new experimental data validating the interaction between *Pf*MD3 and several of the top hits identified by IP-MS. Specifically, interactions between *Pf*MD3 and MPP10, as well as AMP-deaminase (AMPD), were verified by *in vitro* protein pulldown assays (Figs S6C and S6D; shown below). Additionally, sucrose gradient fractionation was performed to more precisely characterize the association of *Pf*MD3 with mRNPs and ribosomal subunits (Figure 6C; shown below). We have also revised this section of the results to more clearly describe the interactions supported by these complimentary methods, and have transferred conclusions that are not yet directly validated to the discussion, where they are presented with appropriate caution.

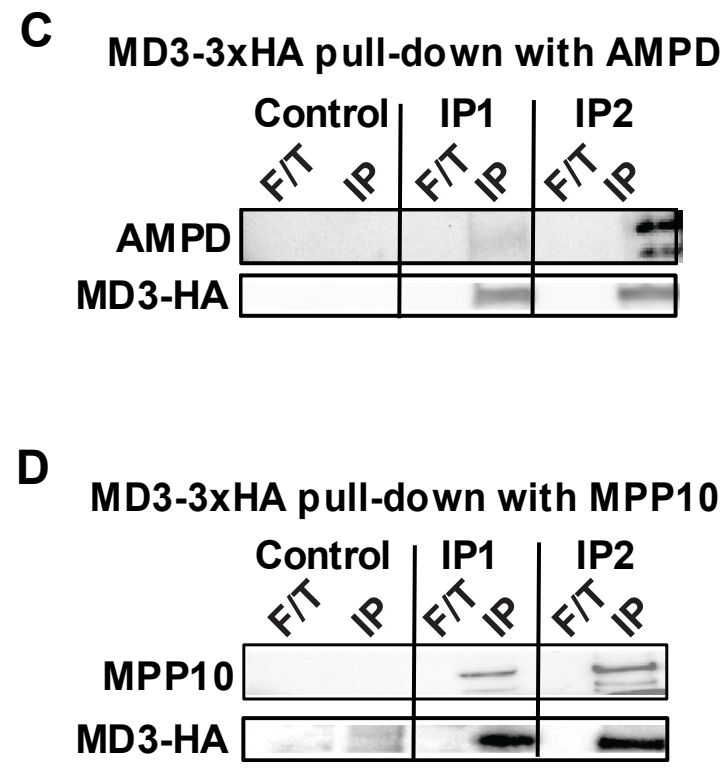

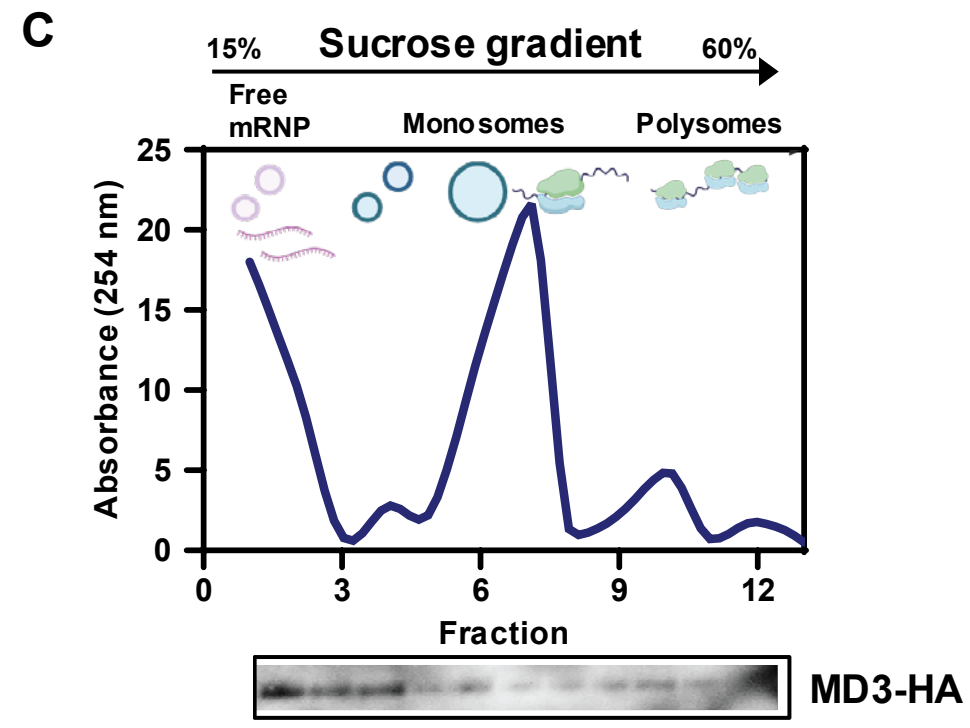

Reviewer #5 (Remarks to the Author):

I co-reviewed this manuscript with one of the reviewers who provided the listed reports. This is part of the Nature Communications initiative to facilitate training in peer review and to provide appropriate recognition for Early Career Researchers who co-review manuscripts.

RESPONSE TO REVIEWER COMMENTS

Dear Reviewers,

We are pleased to submit our further revised manuscript addressing the remaining concerns raised by the Reviewers. We sincerely thank the Reviewers for their continued, thoughtful engagement with our work.

The insightful comments provided in this round of review have allowed us to further strengthen the rigor and clarity of our scRNA-seq analysis and to ensure that our interpretive language accurately reflects the scope of our data.

We believe these revisions fully address all outstanding concerns and we look forward to your response.

Sincerely,

Heather J. Painter, Ph.D.

Senior Staff Fellow - Principal Investigator

U.S. Food & Drug Administration

Reviewer #1 (Remarks to the Author):

Van Biljon et al. provide a revised manuscript that has significantly addressed the concerns we raised, incorporated many of the suggestions and produced a much stronger manuscript. Other than some minor formatting problems, we have no further suggestions or comments on the manuscript.

We sincerely thank Reviewer #1 for their generous and encouraging assessment of our revised manuscript. We are pleased that the revisions have addressed their concerns and strengthened the work to their satisfaction.

Minor comments

Line 135: The wording in the following sentence is unclear and we recommend modifying. It implies whole genome sequencing can detect mRNA changes. This seems likely to be dealing with two different datasets (gDNA and mRNA sequencing).

'and whole genome sequencing (Figures S2C) which revealed a loss in pfmd3 mRNA abundance in gametocytes by RNA sequencing from ΔPfMD31.1 (Figure S2D, Table S1).'

We appreciate the reviewer for drawing attention to this ambiguous phrasing. The sentence has been revised to more clearly distinguish between the genomic and transcriptomic datasets as follows:

"We confirmed the genetic disruption in two clones, Δ*Pf*MD3$^{1.1}$ and Δ*Pf*MD3$^{1.2}$, by genotyping PCR (Figure S2B) and whole genome sequencing (Figures S2C). This disruption resulted in a loss of *pfmd3* mRNA abundance in gametocytes, as measured by RNA sequencing from Δ*Pf*MD3$^{1.1}$ (Figure S2D and Table S1)." (Lines 134-137)

Line 1625: 'with its male a development defect'. Is this meant to read 'with a male…..'?

We thank the reviewer for identifying this typographical error. The figure legend has been corrected as follows:

"Figure 3. Altered transcript and protein abundance in Δ*Pf*MD3 is strongly associated with a male developmental defect."

Reviewer #4 (Remarks to the Author):

The authors have done a thorough revision based on all other reviewers' comments. I have two further comments on the revised items:

We are grateful to the reviewer for their positive assessment of our manuscript and for recognizing the substantial revisions undertaken in response to the previous round of review.

The authors have made a significant effort by incorporating a single-cell dataset, but they don't exploit it to its full potential. It would be useful to incorporate a representation of developmental stages in each branch. The authors state "cells map along the entire male developmental trajectory"—this is too vague. We need to see the sampling of cells at different differentiation stages. It would also be useful to isolate the male branch and plot the integrated object without the MCA data to gauge the qualitative difference of the parasites during differentiation. There is a missed opportunity to perform dynamic DE in pseudotime to understand the timing of the MD3 effect. I think the authors should also include a comparison of other stages to contextualize the potential role (even if not essential) at other developmental stages, including in stalk cells.

We thank the reviewer for their thoughtful comments regarding the scRNA-seq analysis and agree that this dataset provides a valuable opportunity to further investigate the developmental timing of *Pf*MD3-dependent effects during gametocytogenesis.

To better represent developmental states captured within the Δ*Pf*MD3$^{1.1}$ dataset, we have revised the manuscript and supplementary figures. We now clarify in the figure legend that Figure S4D represents an independent UMAP of ΔPfMD3$^{1.1}$ cells encompassing the complete 12-day developmental time course, including both asexual and sexual stages, rather than a projection onto the NF54 reference malaria cell atlas. In addition, we have included a new diffusion map visualization of Δ*Pf*MD3$^{1.1}$ sexual gametocyte subset (Figure S4E). This analysis demonstrates that, "Despite the loss of *Pf*MD3 expression, the subset of Δ*Pf*MD3$^{1.1}$ sexual cells segregate into distinct early-developing gametocyte, male, and female clusters (Figure S4E), indicating that sex-specific transcriptional programs remain detectable in the mutant population." (lines 216-219).

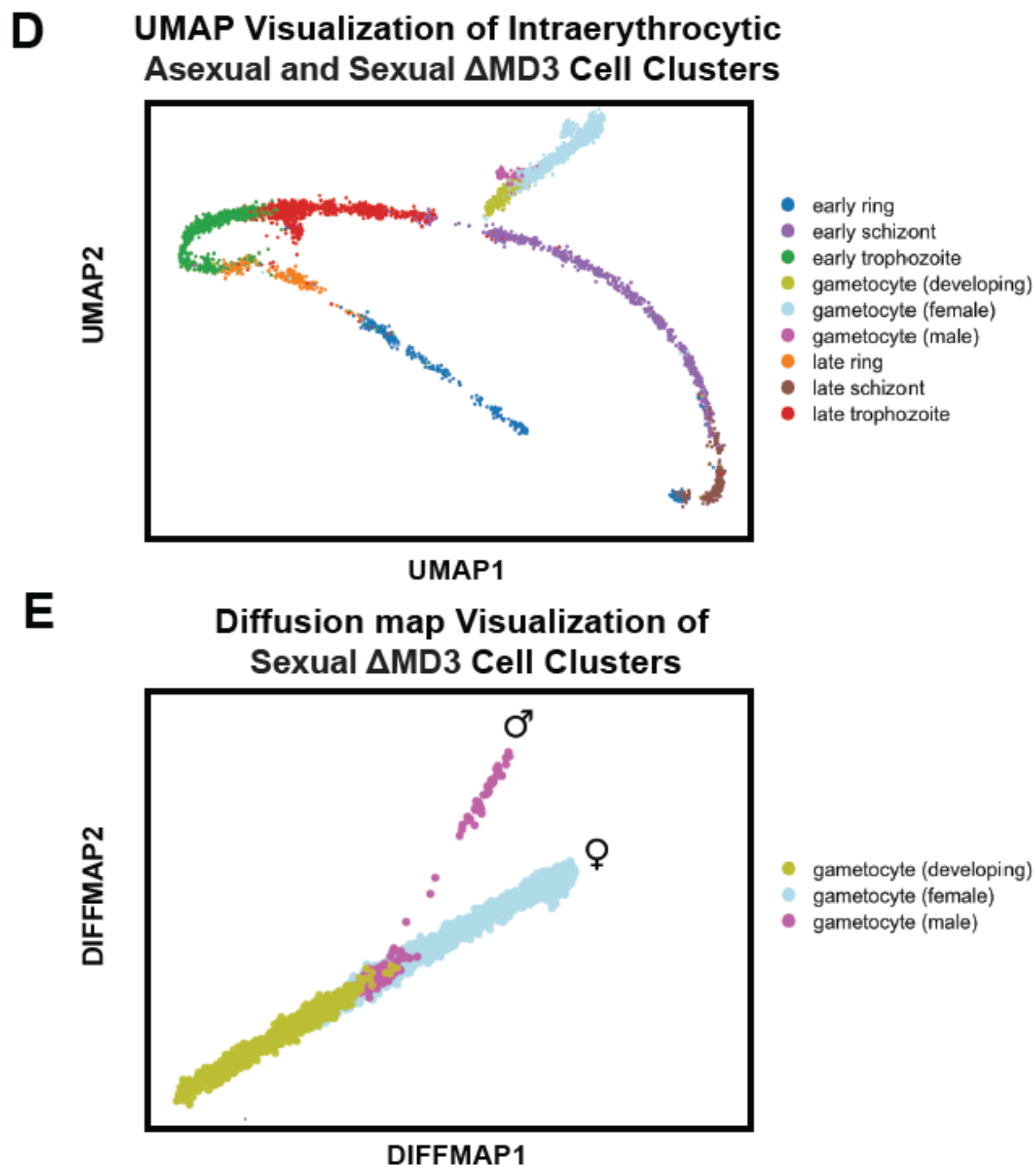

To address the reviewer's suggestion regarding dynamic differential expression analysis, we performed trajectory-based differential abundance analysis using tradeSeq across early gametocyte populations (stalk and branch) prior to lineage bifurcation, as well as along the male and female trajectories. These analyses identified transcriptional perturbations both before and after lineage divergence, which are provided in Table S7. Specifically, we have "identified 110 transcriptional changes shared during early gametocytogenesis (n = 67 increased and n = 43 decreased in Δ*Pf*MD3[1.1]). These included increased abundance of ribosomal and translation elongation factors, whereas genes involved in cytoskeletal organization *i.e.*, formin 1 (*pf3d7_0530900*)[78], dynein intermediate light chain (*pf3d7_0906400*)[79], ARP2/3 complex subunit 1 (*pf3d7_1118800*)[80], and tubulin–tyrosine ligase (*pf3d7_0514000*)[81] were reduced. It is possible that these changes associated with early gametocytes contribute to the reduced capacity for cellular remodeling required for male gametocytogenesis in Δ*Pf*MD3 parasites following lineage branching." (lines 236-244).

Notably, these early transcriptional changes coincide with the peak of *Pf*MD3 expression observed during Stage II gametocytogenesis in our bulk transcriptomic dataset, supporting a role for *Pf*MD3 during the earliest stages of sexual development. Following lineage bifurcation, a substantial set of genes remained differentially abundant across both male and female lineages (n = 225), indicating that the transcriptional consequences of *Pf*MD3 loss persist beyond early gametocytogenesis and culminate in targeted male-specific transcriptional changes (n = 129) during male gametocytogenesis.

Consistent with the reviewer's suggestion to examine earlier developmental stages, we have now incorporated the outcomes of our trajectory-based analysis of all sexual clusters into the manuscript. Together, these additions strengthen

the temporal interpretation of the scRNA-seq dataset and provide a more comprehensive assessment of *Pf*MD3-dependent transcriptional perturbations both before and after lineage bifurcation.

L166. This suggests that the overall decrease in gametocytemia is due to a sex-specific impact of PfMD3 in males. I don't think this is shown by the data. The male/female bifurcation is observed around stage III, yet the loss is already seen in stage II. There is also still a question of the numbers here: the % of gametocytes that are PF47+ or P230+ in the KO don't add up to 100%, suggesting the presence of potentially double-negative parasites (although I understand this is not double-stained). I am not suggesting new experiments but the authors should be more cautious.

We agree with the reviewer that our previous statement could have been interpreted ambiguously, particularly given that the impact of *Pf*MD3 deletion prior to Stage III gametocytogenesis cannot be directly assessed by measuring P230p and PF47 expression in Stage V gametocytes. We have therefore moderated our claim in the revised manuscript as follows:

"This suggests that *Pf*MD3 plays a role in the formation of male gametocytes. However, the loss of *Pf*MD3 parasites does not abrogate progression through gametocytogenesis as Δ*Pf*MD3 Stage V gametocytes appeared morphologically similar to wild-type parasites (Figure S2G)." (Lines 167-170)

We also agree with the reviewer that the P47+ and P230p+ counts cannot be directly compared, as both antibodies were raised in mice, precluding the simultaneous double-staining of the same slides. Our interpretation was therefore restricted to comparing the proportion of P47+ and P230p+ cells between the NF54 parent and the Δ*Pf*MD3 populations independently, rather than making direct comparisons between the P47+ and P230p+ populations in the same culture. We acknowledge the possibility of a double-negative gametocyte population cannot be excluded with the reagents and tools currently available to us, and we have ensured that our conclusions are appropriately cautious in this regard.
