# Supplementary material for: The CCCH-type zinc-finger PfMD3 promotes translation for malaria parasite transmission
Source: Nat Commun. 2026 Nov 3;17:11370. doi: 10.1038/s41467-026-77156-1 (PMC13731652; doi:10.1038/s41467-026-77156-1)

Supplementary information to:

The CCCH-type zinc-finger *Pf*MD3 promotes translation for malaria parasite transmission

Riëtte van Biljon[1] *et al.*

[1]Laboratory of Mucosal Pathogens and Cellular Immunology, Division of Bacterial, Parasitic, and Allergenic Products, Office of Vaccines Research and Review, Center for Biologics Evaluations and Research, Food and Drug Administration, Silver Spring, MD, USA

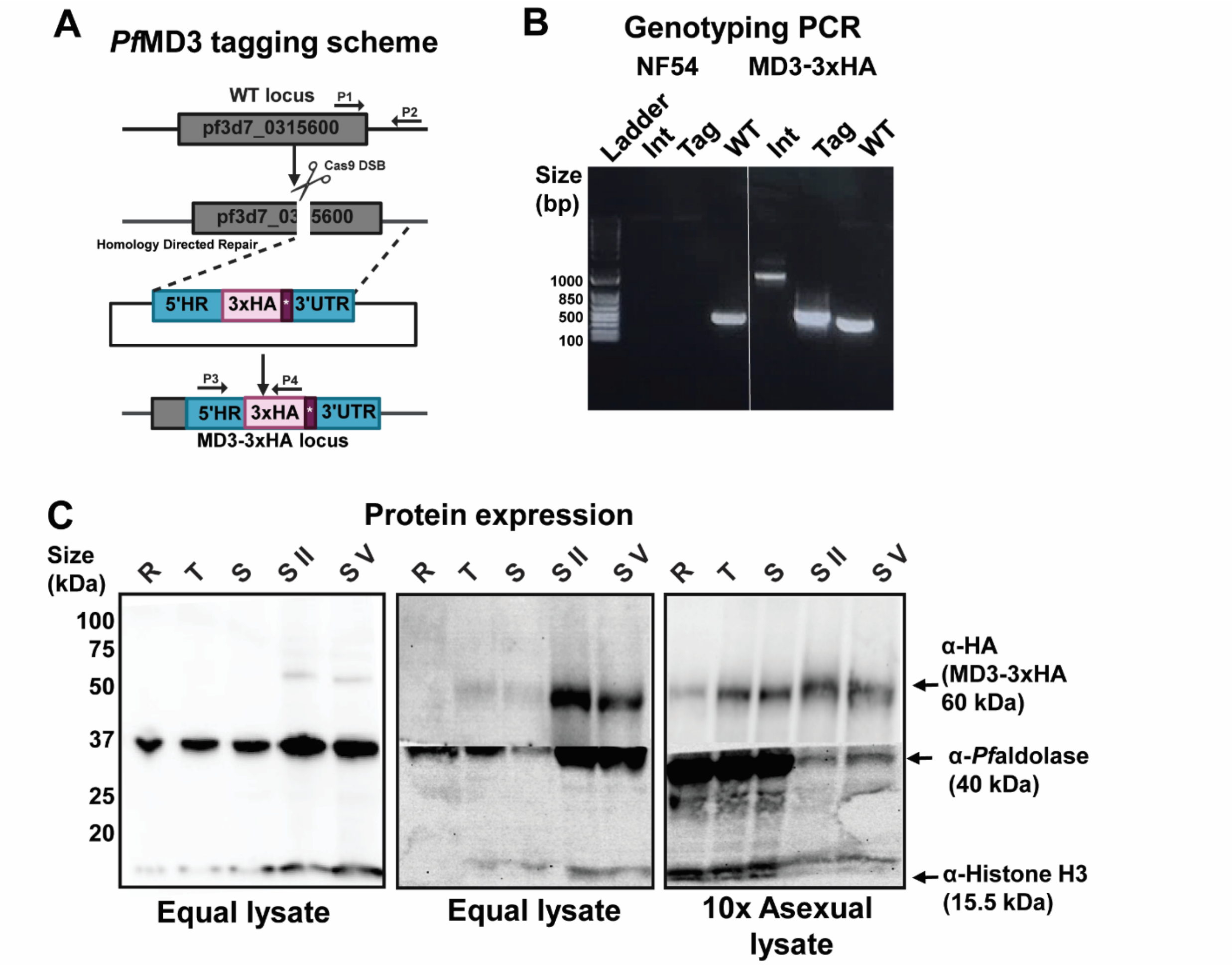

Figure S1. Verification and expression of *Pf*MD3-3xHA, related to Figure 1. (**A**) A CRISPR-Cas9 based approach was used to produce a clonal *Pf*MD3-3xHA tagged line in NF54e parasites (MRA-1000A) (schematic created in Biorender https://BioRender.com/itoihwh). (**B**) Primers P1+P2 (Table S6) were used as a positive control for the *Pf*MD3 locus (WT, 485 bp) while plasmid uptake was verified with P3+P4 (Tag, 612 bp) and integration of the tag at the 3' end of *Pf*MD3 verified with P1+P4 (Int, 1095 bp). (**C**) Protein expression of *Pf*MD3 was verified by Western blot showing the uncropped membranes quantified in Figure 1D here, with signal intensity compared to the *Pf*aldolase control quantified by densitometry in (D). Abbreviations: R = ring-stage, T = Trophozoite, S = Schizont, S II = gametocyte stage II, S V = gametocyte stage V, C = cytoplasmic extract and N = nuclear extract. Different lengths of exposure were applied to membranes for visualizing expression in asexual stages and gametocytes in order to obtain a clear image.

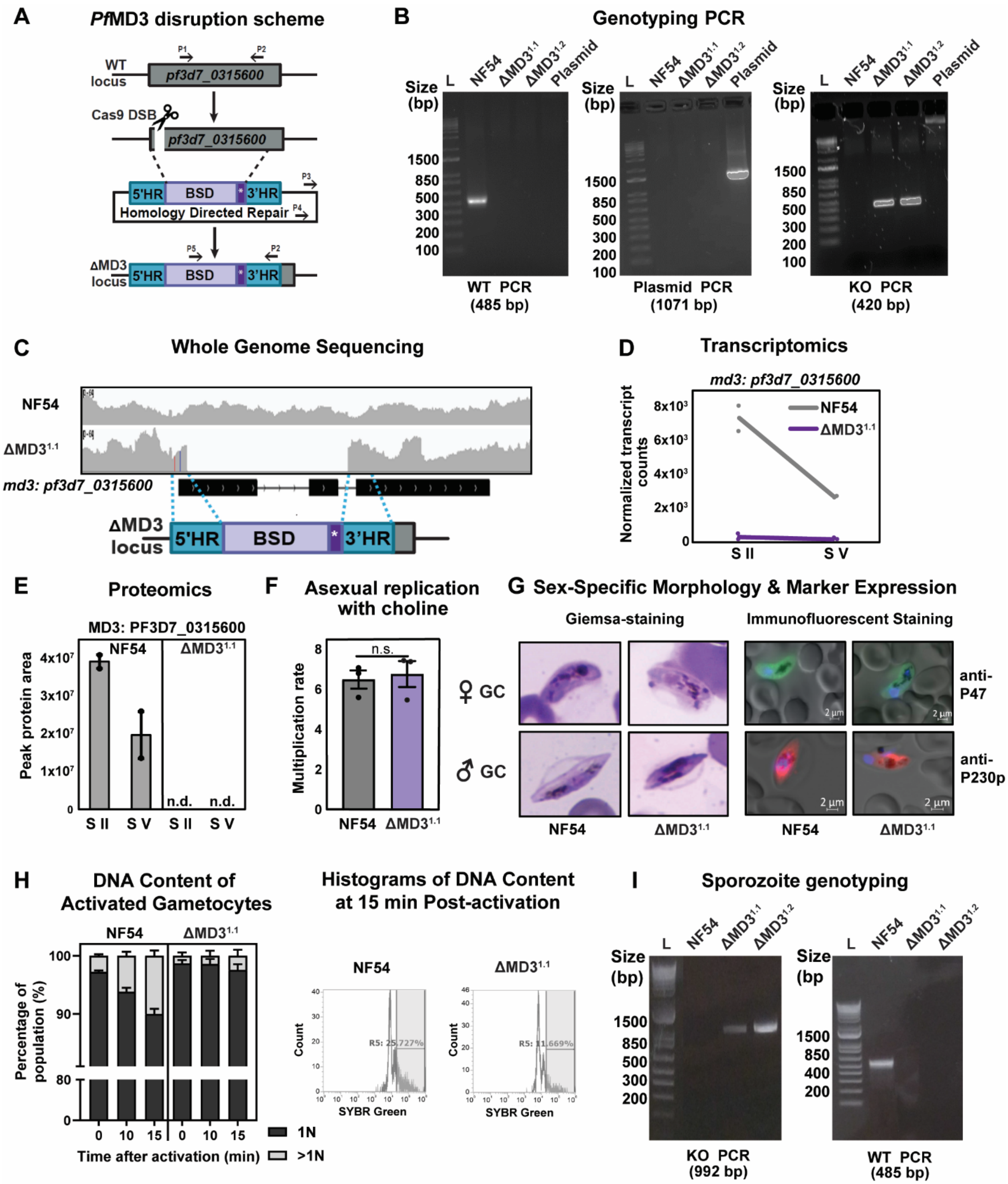

Figure S2. Verification of *Pf*MD3 genetic knockout, related to Figure 2. (**A**) Knockout strategy (schematic created in Biorender https://BioRender.com/itoihwh) and (**B**) genotyping PCR verification of the NF54e parent (WT PCR, P1 + P2, *Pf*MD3$^{1.1,1.2}$ (KO PCR, P5 + P2), and the CRISPR Cas9 plasmid (P3 + P4) by replacement of the 1st two exons of *Pf*MD3 with a blasticidin (BSD) resistance cassette following a Cas9 mediated double stranded break (DSB). (**C**) IGV screenshot of the MD3 locus from whole genome sequencing of the NF54e parental line and Δ*Pf*MD3$^{1.1}$. Verification of the expression of the *Pfmd3* transcript in both the Δ*Pf*MD3$^{1.1}$ and NF54e parent strain from (**D**) next-generation RNA-sequencing and (**E**) *Pf*MD3

protein expression with DIA-MS throughout gametocyte maturation (n = 2). (**F**) Relative multiplication rate of Δ*Pf*MD3$^{1.1}$ and NF54e parent strain was determined in the presence of 2 mM choline chloride to prevent conversion to gametocytes in the culture (n = 3) and significance tested with a two-tailed t-test, graph created in Graphpad Prism v10.0. (**G**) Representative morphology of mature stage IV-V gametocytes was captured for the NF54e parent compared to Δ*Pf*MD3$^{1.1}$ using light microscopy imaging of Giemsa-stained thin blood smears and immunofluorescent microscopy using either P230p antibody with mouse Alexa-568 secondary antibody to visualize male gametocytes or P47 antibody with rabbit Alexa-488 secondary antibody to visualize female gametocytes. (**H**) The ability of Δ*Pf*MD3 to produce male gametes was investigated by inducing exflagellation of gametocytes (day 15) and measuring nuclear content (with example gating strategy next to graph) via flow cytometry following staining with SYBR Green I in technical triplicate. (**I**) PCR confirmation that sporozoites generated from the NF54e parent and Δ*Pf*MD3$^{1.1}$ parasites to the mosquito, retained either the wild-type or disrupted MD3 locus (Primers in Table S6). Where appropriate, individual values for replicates are shown as dots.

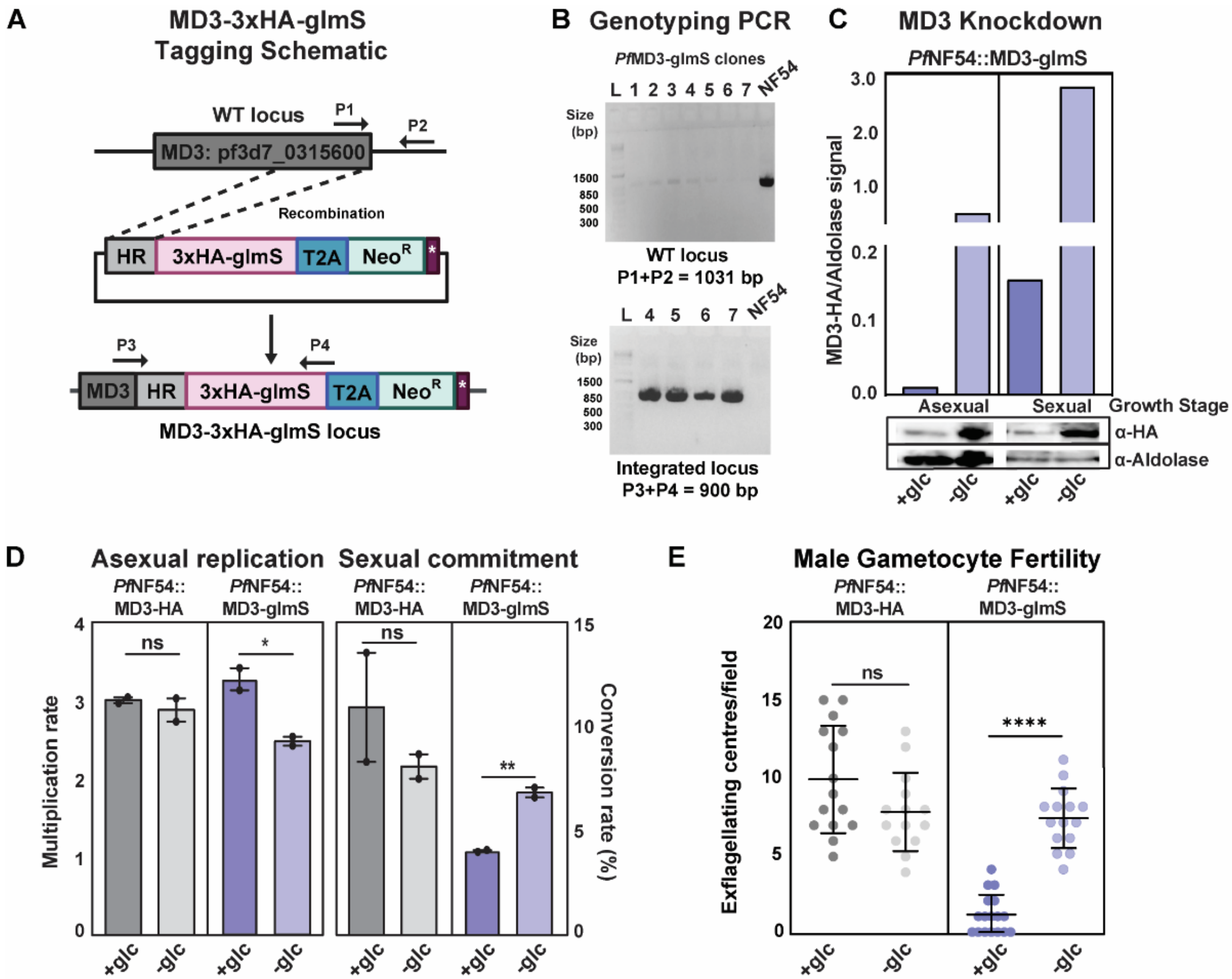

Figure S3. Confirmation of the role of *Pf*MD3 in maintaining *P. falciparum* male fertility. (**A**) The pSLI-glmS plasmid was used to produce a clonal line of *Pf*MD3-3xHA-glmS that would allow for glmS-ribozyme mediated knockdown of *Pf*MD3 mRNA upon addition of glucosamine (schematic created in Biorender https://BioRender.com/itoihwh). (**B**) PCR verification of *Pf*MD3-3xHA-glmS clones (1-7) with NF54e parent strain control, clone 7 was used for follow-up experiments. (**C**) Bar graph shows the degree of protein knockdown measured by western blot (HA signal compared to *Pf*aldolase) obtained for *Pf*MD3-3xHA-glmS following treatment with 1.25 mM glucosamine for 72 h, starting at either 8-12 h post-invasion in asexual development or Stage I of gametocytogenesis for sexual parasites. Following treatment with glucosamine, *Pf*MD3-3xHA glmS or *Pf*MD3-3xHA parasites were assessed for (**D**) asexual blood stage replication rate, percent gametocyte conversion rate, or (**E**) The ability of the parasite lines to produce male gametes and significance was tested using a two-tailed t-test. Each experiment was conducted with n = 2 biological replicates and for panels B and C parasitemia was counted using flow cytometry with at least 10 000 cells counted per sample while for D, exflagellation of gametocytes was induced on day 15 and the number of exflagellating centers per field were counted on a hemocytometer.

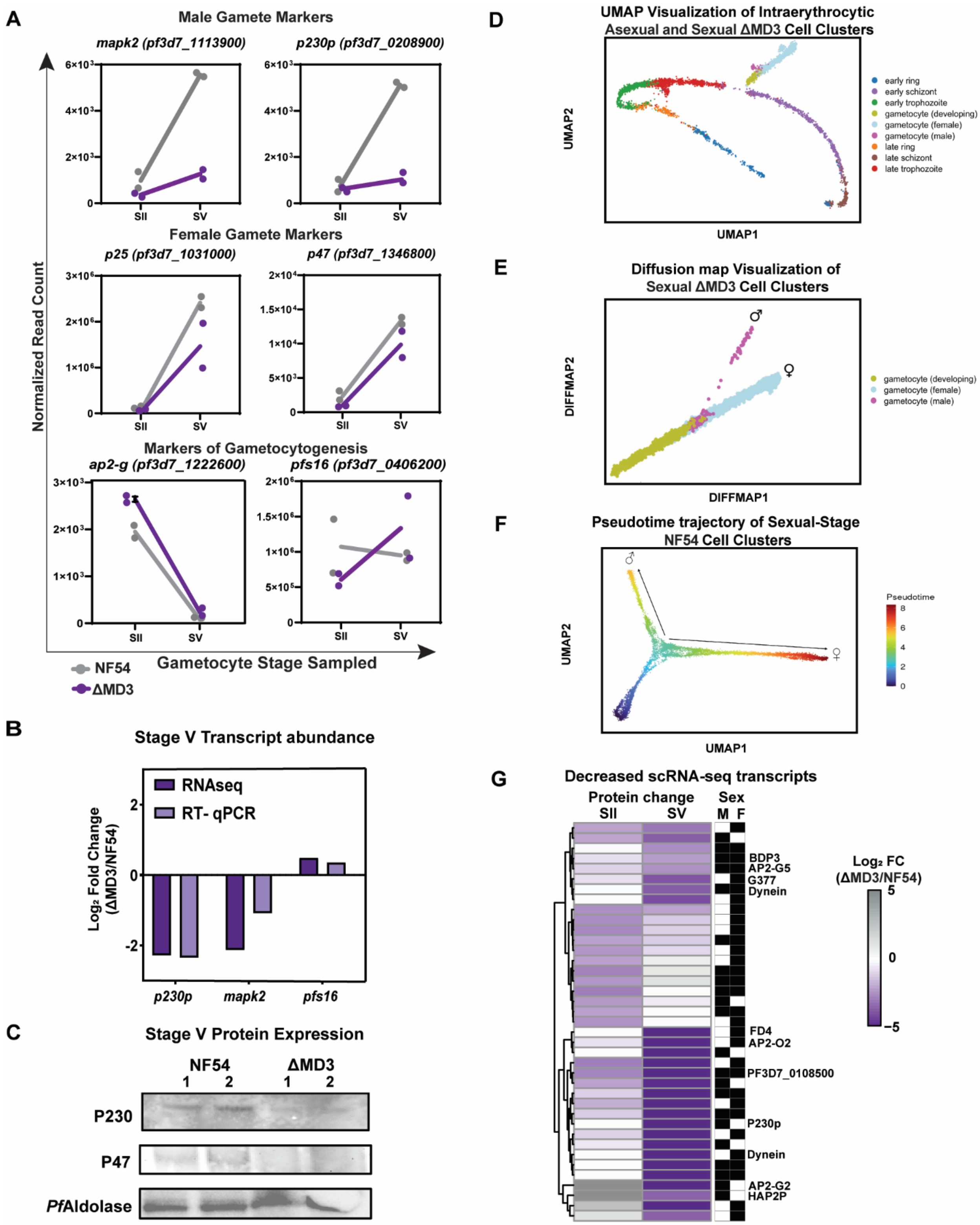

Figure S4. Molecular profile of Δ*Pf*MD3 gametocytes, related to Figure 3. (**A**) Known markers of gametocytogenesis and male or female development are plotted as line graphs with individual values for replicates shown as dots for the WT and KO lines respectively (n = 2) (Associated data in Table S2). (**B**) Comparison of transcript abundance of male markers P230p, MAPK2 and Pfs16 gametocyte markers in

Δ*Pf*MD3 compared for NF54 as measured by qPCR and RNA-seq respectively. (**C**) Validation of differential expression of P230 and P47 from proteomics data with western blot for n = 2 biological replicates (1, 2) as compared to *Pf*aldolase loading control. (**D**) UMAP clustering of all *Pf*MD3$^{1.1}$ cells encompassing the 12-day developmental time course, including both asexual and sexual stages and (**E**) diffusion map visualization of *Pf*MD3$^{1.1}$ sexual cells segregated into distinct clusters of early developing, male, and female gametocyte populations. (**F**) Slingshot-based lineage and pseudotime inference overlaid on the UMAP embedding of 3,106 subsetted ΔPfMD3$^{1.1}$ cells integrated with 7,988 subsetted NF54 gametocyte cells from the Malaria Cell Atlas. (**G**) Heatmap representation of differentially abundant scRNA-seq transcripts from either the male or female lineage (M, F) against Stage II and Stage V proteomics.

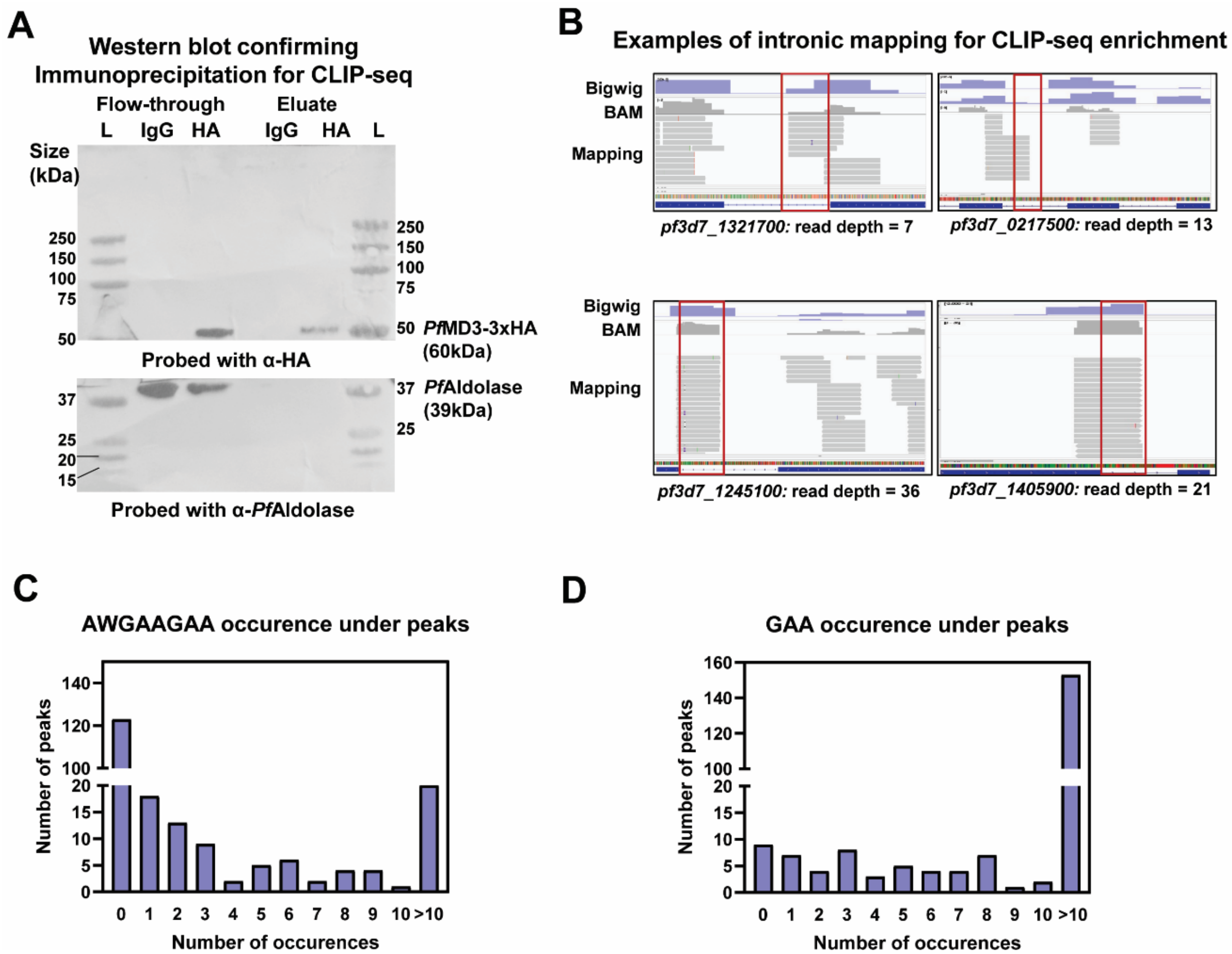

Figure S5. Confirmation of immunoprecipitation, intronic binding and sequence specificity from *in vivo* CLIP-seq and *in vitro* RBNS results, related to Figure 4. (**A**) Western blot shows chemiluminescent visualization of anti-HA and anti-*Pf*aldolase staining for the *Pf*MD3-3xHA immunoprecipitation with rat anti-HA antibody (1:2500) and rat anti-IgG antibody (1:2500) respectively. L = ladder, IP = Immunoprecipitation. (**B**) Examples of mapping of enriched CLIP-seq regions to introns in bound transcripts in the IP are shown compared to the input control. Bigwig tracks show input subtracted immunoprecipitation samples while the relative coverage over the introns is shown for BAM files filtered for mapping to unique sites in the genome. Enriched regions were visualized in IGV (v2.1.3). (**C, D**) Number of instances of occurrences of enriched motif from CLIP-seq (AWGAAGAA) or GAA are shown for areas occurring under CLIP-peaks.

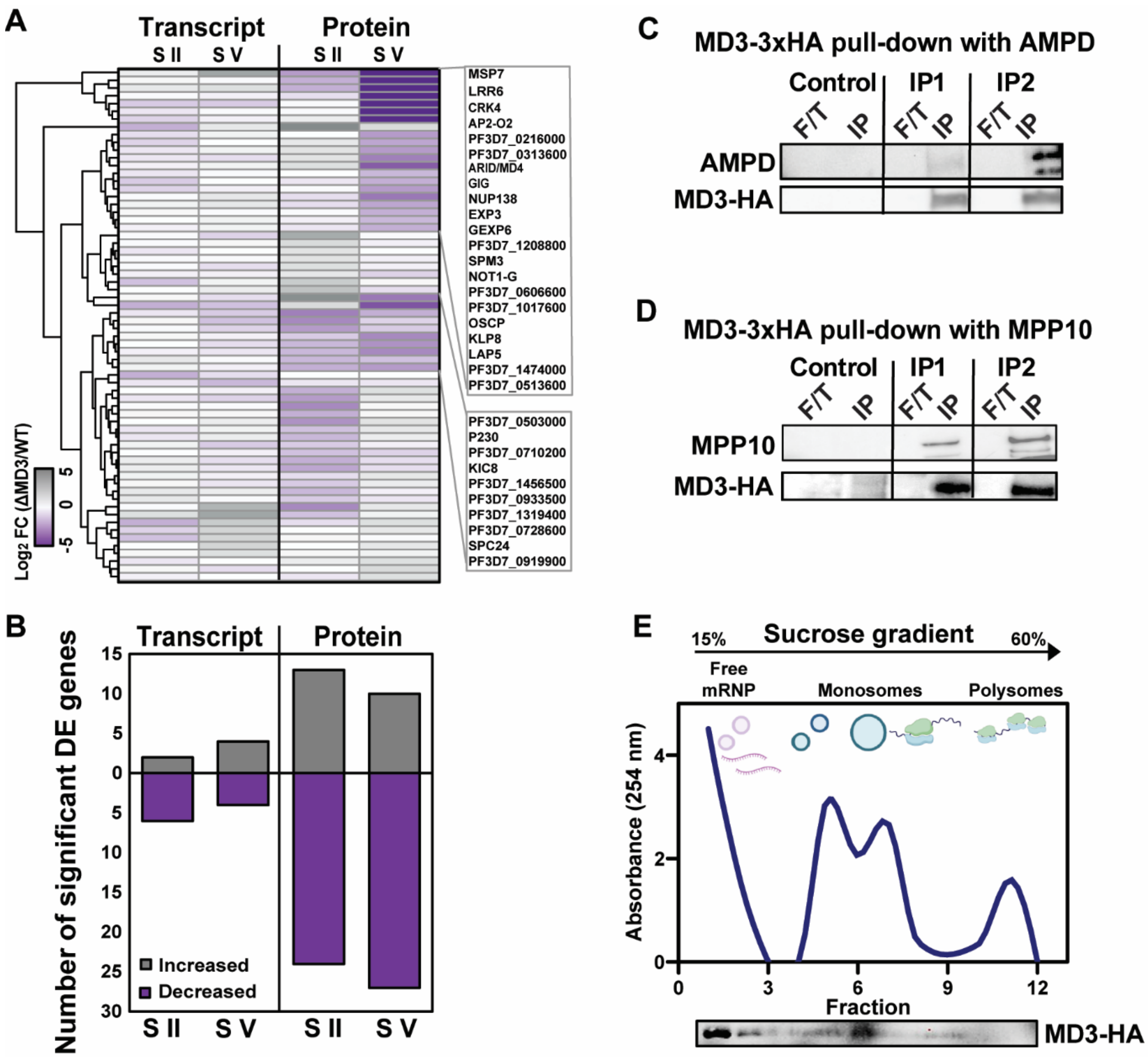

Figure S6. Gene expression of *Pf*MD3 targets change in Δ*Pf*MD3 parasites, related to Figure 5 and western blot images related to Figure 6. (**A**) Heatmap representation of $\log_2$ fold change of the transcript and protein abundance for 66 differentially expressed mRNA targets of *Pf*MD3 at Stage II and V of Δ*Pf*MD3$^{1.1}$ gametocyte development. (**B**) The histogram indicates the number of *Pf*MD3 mRNA targets detected by CLIP-seq that are differentially abundant on the protein or transcript level for each stage respectively. Cropped western blot shows confirmation of interaction of *Pf*MD3-3xHA with recombinant 6xHis-tagged MPP10 (**C**) and AMP deaminase (**D**) using protein pull down from *Pf*MD3-3xHA tagged lysate for n = 2 biological replicates (F/T = flow through, IP = immunoprecipitated sample). (**E**) Shows a western blot image and A_{254} trace related to sucrose gradient (fractions 1-11) profiling obtained for MD3-3xHA from a second biological replicate obtained as in Figure 6E. Trace graph was created in Graphpad Prism v10.1.0. with inset images showing mRNP/Monosomes and Polysomes created in BioRender. Van Biljon, R. (2026) https://BioRender.com/8j22dli.

Supplementary Note:

1. ***Pf*MD3 Orthologue Amino Acid Alignment** 10
2. ***Pf*MD3 and *Pb*MD3 Amino Acid Alignment** 12
3. **NCBI conserved domain search results** 13
4. **Cobalt multiple sequence alignment results comparing *Pf*MD3 to human RRBP1** 13
5. **Results from Nucleolar localization signal search:** 14

---

1. *Pf*MD3 Orthologue Amino Acid Alignment

Notations include: CCCH domain, nucleolar localization signal and <u>Ribosome receptor lysine/proline rich domain</u>

```
HEP_00157900        ---MTNAL-------SKEKVKSELEEKIKTWKELAQYKGLQNMSIERCFINVHHNEHNFQ
PocGH01_08032900    -------MTNSMEREHREELGKKLSEKAKKWLASSETKNSLCNSMKKRFIRMQREKYFSL
PRELSG_0410000      ------MDNNSKVNEHIENLNNTIIQKYKIWVDLPETRNILSTSVSRSFINIPYEKYKFN
PGAL8A_00400700     MDGNSKINNHE----HTENLNDAIIQKYKIWVDLPETRNILSTSVSRSFINIPYENYKFN
PGABG01_0315000     MNSNNNMLNNNSDNTHTGSINNGVPERDQEWVDSPETRNMLNSSVHKTFINIPYDNYYLG
PADL01_0314500      MNSNNNMLNNNSDNTPTGSINNDIPERDQEWVDSPETRNILNYSVNKTFINIPYENYYLG
PRCDC_0314900       MNSNHNMLKNNFTNAHTGYLNNGMPERCQVWVDLPETRNMLNSSVGKTFINIPYENYEFG
PF3D7_0315600       MNSNHNMLKNNFTNAHTGYLNNDMPERCQAWVDLPETRNMINSSVGKTFINIPYENYEFG
PPRFG01_0317000     MNSNHNMLKNNFTNAHNGYLNNDMPERSQAWVDLPETRNMINSSVGKTFINIPYENYEFG
PBILCG01_0315100    MNSNHNMLKNNFTNAHTGYPNKGMPERCKAWVDLPETRNMINSSVGKTFINIPYENYEFG
PBLACG01_0316000    MNSNHNMFNNNFTNAHTGYLNKGMPERCKAWIDLPETRNMLNSSVGKTFINIPYENYEFG
                                         . : :. : *   .: ..    *: . **.:  :::

HEP_00157900        LRRMHTIDTGDLLCHPTLHELPKRKLLNLNQVEA------NEYINQQDKSYNNDTFLMDN
PocGH01_08032900    LSRTYTVDAGDLVDDPKSLGLTEIEEIKRSFSERTYLRREEGFLRNYSSGENNLHHLFNV
PRELSG_0410000      LRRMNTIDTADLINKQKLCELPKISHKNISNTEN------INDYNKTDQIKSMNGDISNN
PGAL8A_00400700     LRRMNTIDTADLINKQKLCELPKIVNKSINSEEN------INDCNETDQIKSVNGDIRKN
PGABG01_0315000     LNELYTIDK-DVFDKCL-CELPKIDNLCTMFRKK------YNTAQNVHKGNNNKVNFHDN
PADL01_0314500      LNELYTIDTEDVFDKYP-CELPKIENLCTMFRKK------DNTAQNEDKGNNNNVDLHEN
PRCDC_0314900       LRRMYTIDTADLTNKQHLCALPKIENSFTMCANK------KNPAQHGNNGNNSHVNLHEN
PF3D7_0315600       LRRMYTIDTSDLTNKQHLCTLPKTEDSFTMCENK------KNPAQHGNNGNNFHANLHEN
PPRFG01_0317000     LRRMYTIDTSDLTNKQHLCALPKTEDSSTMCENK------KNPAQHGNNGNNFHANLHEN
PBILCG01_0315100    SRRMNTIDTADLANKKHLCALPKIENSFTMCANK------INTEQNRNKGNNNHVNLHEN
PBLACG01_0316000    LRRMYTIDTADLANKQLLCALPKIENSCTKCSNI------KNTSQNRKKGNNYHEILHGN
                         *:*  *:        *.:         :           .   .  .    :

HEP_00157900        SGFAFKGIKESMLHENSINRINGINKLSENGKCTGSIFEIKPIIDNNKININETEISALK
PocGH01_08032900    SAF------------------------------------------------PSHLNVANT
PRELSG_0410000      SSL--------------------------------------------------NEIKEEK
PGAL8A_00400700     NNC------------------------------------------------NLNEIKEDK
PGABG01_0315000     RSF------------------------------------------------NVQENDRFT
PADL01_0314500      RIC------------------------------------------------NVQENDRFT
PRCDC_0314900       KSF------------------------------------------------NVQENHRFP
PF3D7_0315600       KSF------------------------------------------------NEQENHRFP
PPRFG01_0317000     KSF------------------------------------------------NEQENHRFP
PBILCG01_0315100    RSF------------------------------------------------NVQENHRFT
PBLACG01_0316000    RNI------------------------------------------------NMQENHRFP

HEP_00157900        NGRTRHTSQTGRNFHIKSIGEL-------SFGTQFHLIGTCNPCRYEWTTGCYLAELCRF
PocGH01_08032900    QVNFKSTSSMYPLNNAIEEGNTRSRYGFPSFGTNFHKMGTCNPCRYEWTRGCHMGRYCRF
PRELSG_0410000      KKNMNVTKIKNQT-NLKSDDTN------PSFGTQFHEKGTCNPCRYEWTRGCHMGRFCRF
PGAL8A_00400700     KKYINGIKVKNQTY-LKNDDTN------PSFGTQFHEKGTCNPCRYEWTRGCHMGRFCRF
PGABG01_0315000     NNNTKDVNLRYPTHNFEYLNST------SSFGTQFHNKGKCKPCKYEWTRGCHMGLFCRF
```

```
PADL01_0314500      HNNTKDVNLRYPTHNFEYLNST------SSFGTQFHNKGKCKPCKYEWTRGCHMGLFCRF
PRCDC_0314900       QNNTKDVDIRNPTHNFQHVNSM------PSFGTQFHHKGRCSPCKYEWTRGCHLGSLCRF
PF3D7_0315600       QNNTKDVDIRNPTYNIQPVNNM------PSFGTQFHHKGRCSPCKYEWTRGCHLGSRCRF
PPRFG01_0317000     QNNTKDVDIRNPTYNIQHVNNM------PSFGTQFHQKGRCSPCKYEWTRGCHLGSRCRF
PBILCG01_0315100    QNNTKDVNNRNPIYNFQDANGV------PSFGTQFHNKGSCSPCKYEWTRGCHLGSFCRF
PBLACG01_0316000    QNNTKDVNIRNTIYNFQHLSSV------PSFGTQFHNKGRCSPCKYEWTRGCHLGSFCRF
                    :   .  .           .          ****:**  * *.**.**** **::.  ***

HEP_00157900        CHDSSHAPEGAKRVIPDFQTLVTTRDKLQNSLLS---------------------NILAS
PocGH01_08032900    CHDSSHVPSGTARVVPDPKTLANSKVKPENILTL-----------------------RT
PRELSG_0410000      CHHSSHVPLGTERVVPDPKILATTKVRPENILTF---------------------RKFAS
PGAL8A_00400700     CHHSSHVPLGTERVVPDPKILATTKVRPENILTF---------------------RKFAS
PGABG01_0315000     CHDRSHVPAGTKRVIPSPQDLAISRVKPQNIFIS---------------TILNENKKNRK
PADL01_0314500      CHDQSHVPAGTKRVIPSPQDLAISRVKPENIFIS---------------TILNENKKNRK
PRCDC_0314900       CHDQSHVPEGTQRVLPNPQELAITRVKPENIFTSPISRNNNAPSVTKPANILNGKKKNRK
PF3D7_0315600       CHDQSHVPEGTQRVVPNPQKLPITRVKAENNFTSPMSRNPDAPFVTKPGNILNGKKKNRR
PPRFG01_0317000     CHDQSHVPEGTQRVVPNPQKLPITRVKAENNFTSPMSRNPDAPFVTKPGNILNGKKKNRR
PBILCG01_0315100    CHDQSHVPEGTQRVIPDPRELAITRVKPENIFTSNVSRNTNAPFVTKPESISNGKRKKRI
PBLACG01_0316000    CHDQSHVPVGTQRVIPDPRELAITRVKPENIFTSNILRNTNAPFVAKPANISNGKKKNRT
                    **  **.* *: **:*. . *  :. . :* :

HEP_00157900        YRTNANGPANPNRPNNACKDKSS------------------KAYQNKNKNKNV------
PocGH01_08032900    TTDSRNPPLLAYPKRYPYDDKGEAD-----------------KILQKGNKDKKS------
PRELSG_0410000      YATSMNSLADVNSSNYIYEDKVKTY-----------------NNNKISSPNRNN------
PGAL8A_00400700     YATSMNSLLDANSPNHIYEDKVKTY-----------------NNSKMSSPNRNN------
PGABG01_0315000     YKKNENQGKRKGMENKIDDKKEEEK-MGNQTKEDKT-NNQT-KEHKTNNQTKEHKTNNQT
PADL01_0314500      YKKNENKGKRKGMKNKIDDKKEEEK-MGNQKKEHRT-NNQK-KEHKTNNQRKEH------
PRCDC_0314900       HKKNTNKRQRTGGENKMNSQKEEEKTMNSKKEEEKKTDSKK-KEDKMGNQKKEY------
PF3D7_0315600       YKKNANKRQRKGGENKMKSQKEEEKKMDSKKEEEKKMDSKKKEEKKMDSKKKED------
PPRFG01_0317000     YKKNANKRQRKGGENKMKSQKEEEKKMDSKKEEEKKMDSKK-KEDKMGNQKKEC------
PBILCG01_0315100    HRKNANKGQRKEKENKIDSKKEEKK-MDSKKEEEKM-DSKK-EEKKMNSKKEDK------
PBLACG01_0316000    HNKNANKGQSTGGEHKIDSKKEEEK-TDSKKKEEKK-DSKK-KEEKTDSKKKED------
                       . *         .   ..* .                   :  :   .    .

HEP_00157900        ----------------------------------------MNNNQNNPRIN-KKKFNKV
PocGH01_08032900    ---------------------------------------FSNNTNNFRNGSILKREGKN
PRELSG_0410000      ----------------------------------------LNNTNNYKNGN------SY
PGAL8A_00400700     ----------------------------------------INNANNYKNGN------NY
PGABG01_0315000     KEDKTNNQTKEDKTNNKTKEDKTNSQTKEDKTNNQTKEDKTNNQTKEDKTNN-QTKEDKT
PADL01_0314500      -------------------------------KTNNQTKEHRTNNQTKEHKTNN-QTKEHRT
PRCDC_0314900       ------------KRNNQKKERKRNNQKKERKRNNQKNERKTNNQKNENKTNN-QKNENKT
PF3D7_0315600       -------------------------------KMGNQKKECKINNQKKERKTNN-QTKERKT
PPRFG01_0317000     ---------------------------------------KINNQKKERKTNN-QTKERKT
PBILCG01_0315100    ----------------------------------------TDSKKEDKTDS-KKKECKT
PBLACG01_0316000    -------------------------------KMSNEKKESKKNNQTKDRKRNN-QTKEHKT
                                                            :. ::     .

HEP_00157900        ATFENNKKLNNYT---STSTNYQNINI-----KNMAINYKNNQHIHVHNVQN--------
PocGH01_08032900    KSQSNNRNQNNNRNQSRNRDQSSN--------RDRSNNRDQSNNRKQSNNRNWKNDRNKR
PRELSG_0410000      KNSSNLKKTSNY----KSTSNYKNTKVY----KNTNIYKNADNFVSINNLNN--------
PGAL8A_00400700     KNNSNFKKTSNY----KSVNNYKSIKVY----KNTNIYKNSDNYVNTNNLDN--------
PGABG01_0315000     NNQTKEDKTNNQTKEDKTNNQTKEDKTNNYSRRNKINHQKNKKSKKMNDNENKRNPQNIG
PADL01_0314500      NNQTKEHKTNNQTKEYRTNNQTKEHRTNNYSRENKINHQKNKKSQKMNDTENKRNPQNIG
PRCDC_0314900       NNQTNENKTNNQTNENKTNNQTKEHNIYNCPRGNKINYQNNKNSKKMNNVVNERNPQNIG
PF3D7_0315600       NNQTNENKTNNQTNENKTNNQTKEHNIYNCPRGNQMNYQNNKNSKKMNNVVNERTPPNIV
PPRFG01_0317000     NNQTNENKTNNQTKENKTNNQTKEHNIYNCPRENQMNYQNNKNSKKMNNVVNERNPPNIV
PBILCG01_0315100    NNQTKENKTNNQTKENKTNNQTKENNINN--QKNKNFHKNNKNSNKMNNIANERNPQNIG
PBLACG01_0316000    NNQTKEHKTNNQTKENKTNNQTKENKINF--QKNKNFHKKNKNSKKMNNIVNERNPKNIG
                     .  :  : .*       .  : ..         :       . .:      :   *

HEP_00157900        ----------------ENIIQLQSSNNNTCVDSHQQNMYNNIYSC--NLLIPMRVPNYLQ
PocGH01_08032900    IYEHMNNGSNDFVLNRNDNGIFGNYNHTNRYRNFEQNLYNN--DNMDKQEIFNNKNTNI-
PRELSG_0410000      ----------------TSYLNFKYYNDTNYFNNYEN--LSYCYNR--TKLFSNNKDNEF-
```

```
PGAL8A_00400700        ----------------TGYLNFKYYNDTNYFNNYEN--LSNCYNR--TKLLTNNKDNEF-
PGABG01_0315000        IFQRGRNQRRK--RNYNGYSKLNAWNYEYNYLPYKDYFFNYYYNTIISNVFED-------
PADL01_0314500         IFQRRRNQRRK--RNYNGYSKLNTWNNEYNYLPYKDYFFNYYYNTFISNVFENEEKNEL-
PRCDC_0314900          NTERNRNQTRK--INYNDYTTLNTSNCTYNHNPHEDDFLNGHLNAIISNIVSHNEENDL-
PF3D7_0315600          NTQRNRNQIKQ--INYNDYTTLNTCNCTYNHNPYKDNFLNGHLNSIISNIVAHNKENDL-
PPRFG01_0317000        NTQRNRNQIKQ--INYNDYTTLNTCNCTYNHNPYKDNFLNGHLNSIISNIVAHNKENDL-
PBILCG01_0315100       NSERNRNHIRP--INYNDYTTLNTWNCTHNYNPYEDDLLICYYNTFISNLVAHNEENAL-
PBLACG01_0316000       NSQGNTNQIRQ--INYNDYTTLNTWNCTYNYNPYEDDFLNCYYNAVISNVVALNEENNL-
                                         .   :   *        .::         .   .   .

HEP_00157900           LVTIPNNPYNVNIINKHGYRQYNYFNMYNNPHFMQNANSYNEFNLVS---
PocGH01_08032900       -----SRPLYTHARNKNTFKKYARSTSFTSNQVEKIAHQQEKTSDE----
PRELSG_0410000         -----HKTLDPFSKNKDTLKNFKELNSITKEYAIKTINSYQRIKLVS---
PGAL8A_00400700        -----HKTLDTFSNNKDTLKNFKELNSFTRESALKTINSYQRIKLVS---
PGABG01_0315000        --------------------------------------------------
PADL01_0314500         -----YKIFGIIVERDKTSEECELLNNFDSDLFVNLMNIHQSNMKPMLRY
PRCDC_0314900          -----SRIFCIIFKRDNAFEDYECLNLFVPDTVIHHMNIYPLNMVPMRRH
PF3D7_0315600          -----CRIFCIIFKRDNAFEDYKCSNLFVPDTVIHHMNIYPLNMAPMRRH
PPRFG01_0317000        -----CRIFCIIFKRDNAFEDYKCSNLFVPDTVIHHMNIYPLNMAPMRRH
PBILCG01_0315100       -----HRIFCIIFKRNNDFEDYECFNLYYPHSSVHHMSIYPPNMVPMWRY
PBLACG01_0316000       -----YKIFCIIFKRDNTFENYECLYFFDPDSVVHTMNIYQPNMVPMWRY
```

2. *Pf*MD3 and *Pb*MD3 Amino Acid Alignment

Notations include: CCCH domain, nucleolar localization signal and <u>Ribosome receptor lysine/proline rich domain</u>

```
CLUSTAL multiple sequence alignment by MUSCLE (3.8)

pbanka          MDIN---MKENKIEKYPIHKKN----KTKRWIDSSETRDILSNSVSNCFINIPQEKSKFK
pfal            MNSNHNMLKNNFTNAHTGYLNNDMPERCQAWVDLPETRNMINSSVGKTFINIPYENYEFG
                *: *   :*:*  : :. : :*     . : *:* .***:::..**.: ***** *: :*

pbanka          LRRMHTIDSADLVYGPKLYELPKIDKINMSSSENKQSSSSERKK--------NKDCNYET
pfal            LRRMYTIDTSDLTNKQHLCTLPKTED-SFTMCENKKNPAQHGNNGNNFHANLHENKSFNE
                ****:***::**.    :*  *** :. .:: .***:...:.  ::        ::: .::

pbanka          KSKTRCLMDTKSESTINSAVVPMESNDDNIPSFGTQFHEKGTCNPCRYEWTRGCHMGKFC
pfal            QENHRFPQNNTKDVDIRNPTYNIQPV-NNMPSFGTQFHHKGRCSPCKYEWTRGCHLGSRC
                :.: *    :...:  *....   ::.   :*:******** ** *.**.********:*. *

pbanka          RFCHHSSHVPIGTARVVPDPKTLATTKVRPENILTN--------------------QKI
pfal            RFCHDQSHVPEGTQRVVPNPQKLPITRVKAENNFTSPMSRNPDAPFVTKPGNILNGKKKN
                **** .**** ** ****:*:.*. *.*..** :*.                       :*

pbanka          ASYTETLNSL----------------------------VESKSPTNSHSDRTQTNNNF--
pfal            RRYKKNANKRQRKGGENKMKSQKEEEKKMDSKKEEEKKMDSKKKEEKKMDSKKKEDKMGN
                  *.:. *.                            ::**.  :.: *  .:.::::

pbanka          -----KLIEHITKFDYNNITTYQDEGSQNNYSISKKLQNLSKLSNNSNKKKIYK----NN
pfal            QKKECKINNQKKERKTNNQTKERKTNNQTNENKTNNQTNENKTNNQTKEHNIYNCPRGNQ
                     *: :: .: . ** *. .. ..*.* . ::: * .* .*:::::**:    *:

pbanka          NYYVGPYNLNNMDPLC-----------------IPYINFSNYMN-DYDQGIYNYFIINPY
```

```
pfal            MNYQNNKNSKKMNNVVNERTPPNIVNTQRNRNQIKQINYNDYTTLNTCNCTYNH---NPY
                  *  .   *  ::*:  :                  *   **:.:*  .  :   :   **:    ***

pbanka          ---FYSTFKNYILPNALVNNLPKNNITK---ILKPAKPIMGNYAIQSIIS----------
pfal            KDNFLNGHLNSIISNIVAHN-KENDLCRIFCIIFKRDNAFEDYKCSNLFVPDTVIHHMNI
                   *  .  .  *  *:.*  :.:*   :*::  .     *:     .   :  :*    ..::

pbanka          -----------
pfal            YPLNMAPMRRH
```

3. NCBI conserved domain search results

Pssm-ID: 428305 [Multi-domain] **Cd Length:** 141 **Bit Score:** 41.61 **E-value:** 1.55e-04

```
                                                       10        20        30
                                                   ....*....|....*....|....*....|....*.
seqsig_MNSNH_5dc550b7b9727e8d654101985b212b05 260 QKEEEKKMDSKKEEEKKmDSKKKEEKKMdSKKKEDK 295
Cdd:pfam05104                                  13 QRRELKKTQSPKSEKKK-KEKKKEKKKK-SKKKEEK 46
```

4. COBALT multiple sequence alignment results comparing *Pf*MD3 to human RRBP1

Query1: PF3D7_0315600
Query2: human RRBP1 (Ribosome receptor lysine/proline rich domain)

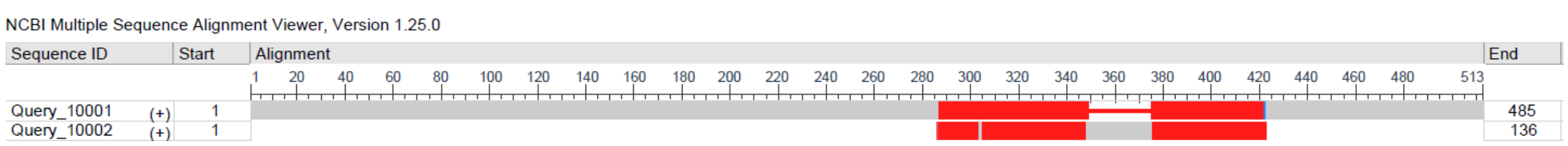

Sequences corresponding to alignment with identity and conservation indicated:

```
287 MDSKKKEDKMGNQKKEC-KINNQKKERKTNNQTKERKTNNQTNENKTNNQTNENKTNNQTKE----------------
-
1
MKETSYEEALANQRKEMAKTHHQKVEKKKKEKTVEKKGKTKKKEEKPNGKIPDHDPAPNVTVLLREPVRAPAVAVAPTP

    ----------HNIYNCPRGNQMNYQNNKNSKKMNNVVNERTPPNIVNTQRNRNQIKQ 485
    VQPPIIVAPVATVPAMPQEKLASSPKDKKKKEKKVAKVEPAVSSVVNSIQVLTSKAA 136
```

5. Results from Nucleolar localization signal search:

NoLS predictions for protein PF3D7_0315600

228-PGNILNGKKKNRRYKKNANKRQRKGGENK-256

273-EEKKMDSKKKEEKKMDSKKKEDKMGNQKKECKINNQKKERKTNNQTKERKTNNQT-327

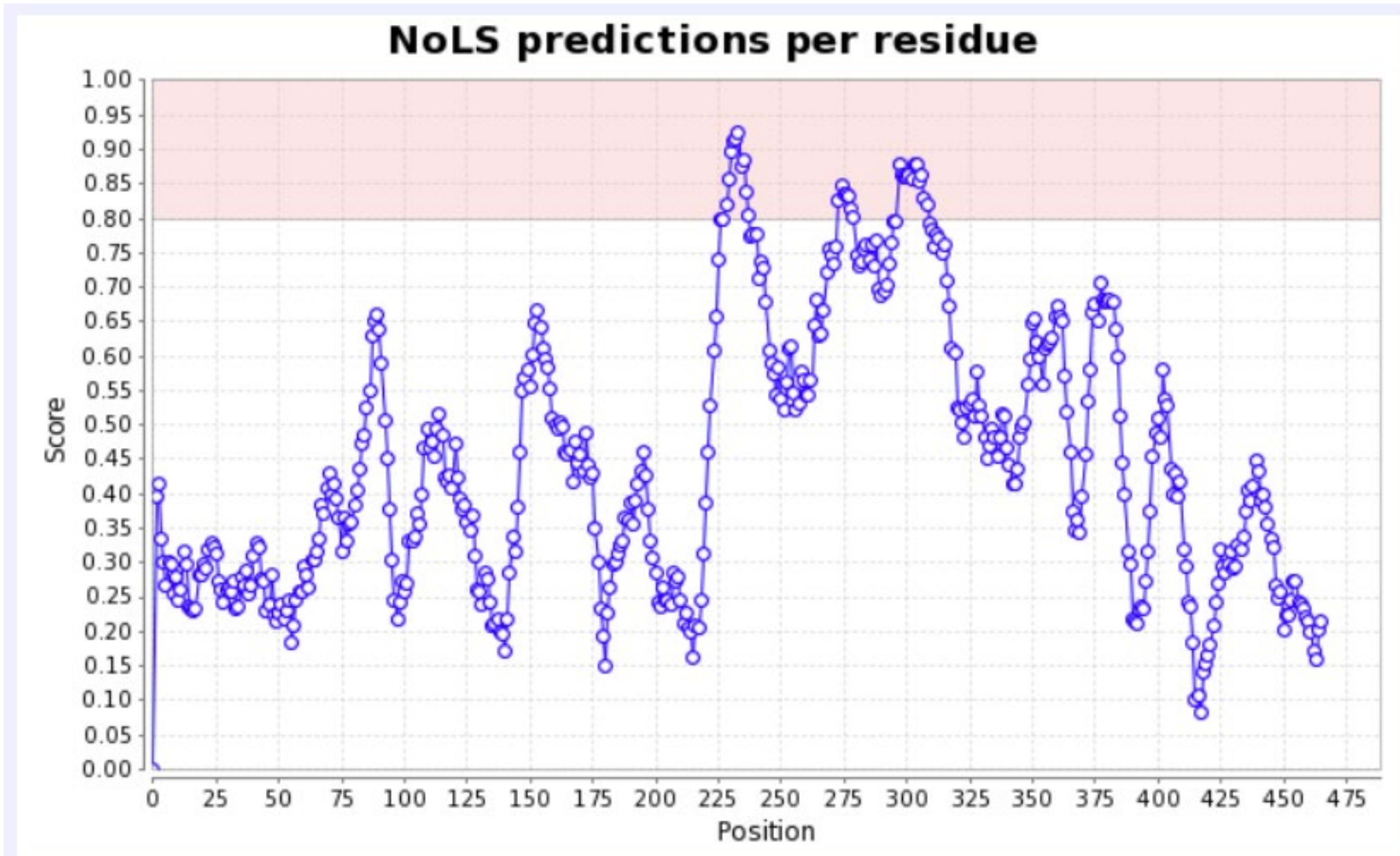

Supplementary source data

Uncropped gel and western blot images:

Figure S1B

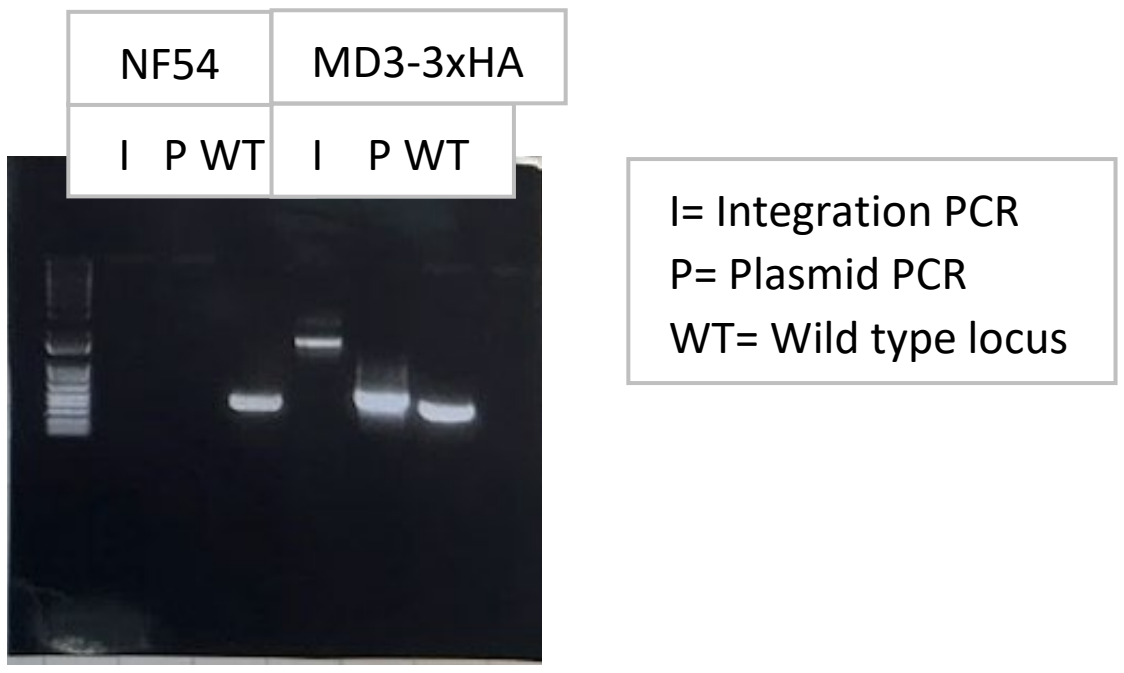

Figure S2B

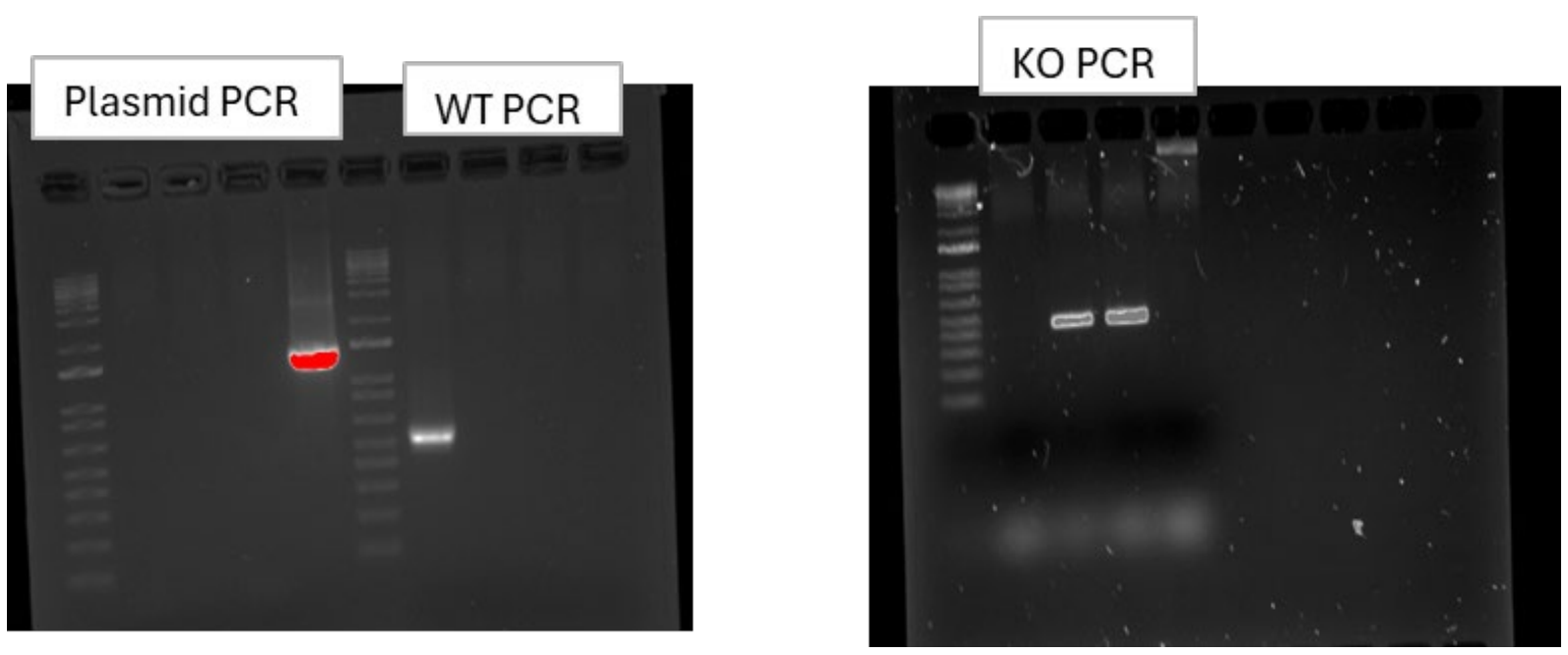

Figure S2I

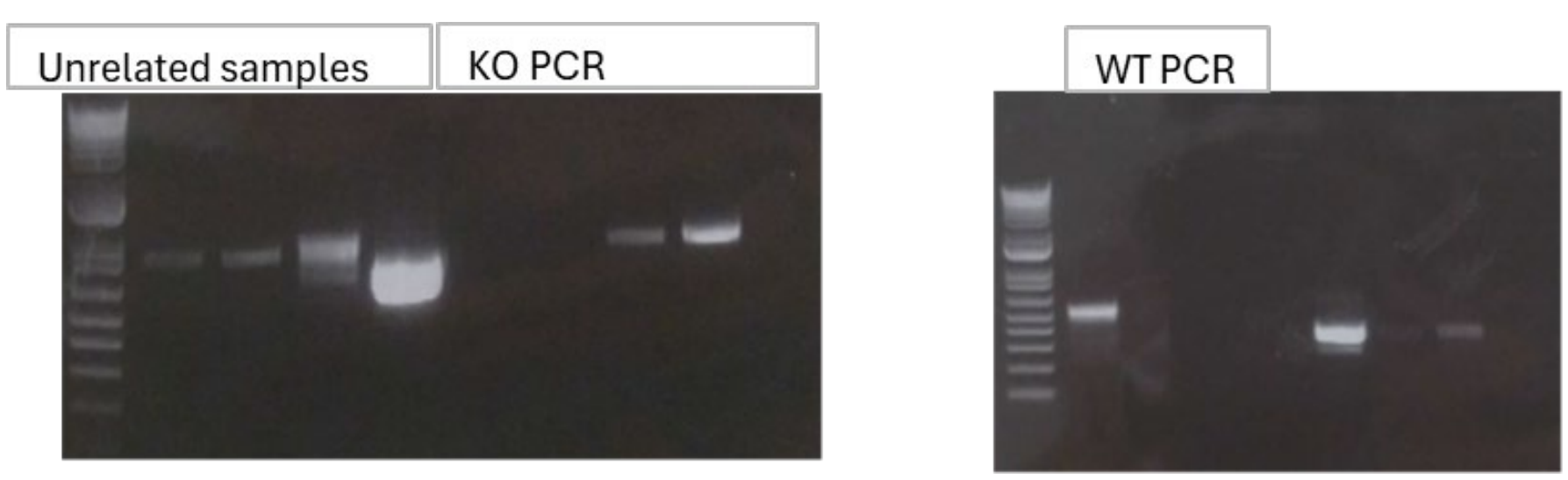

Figure S3

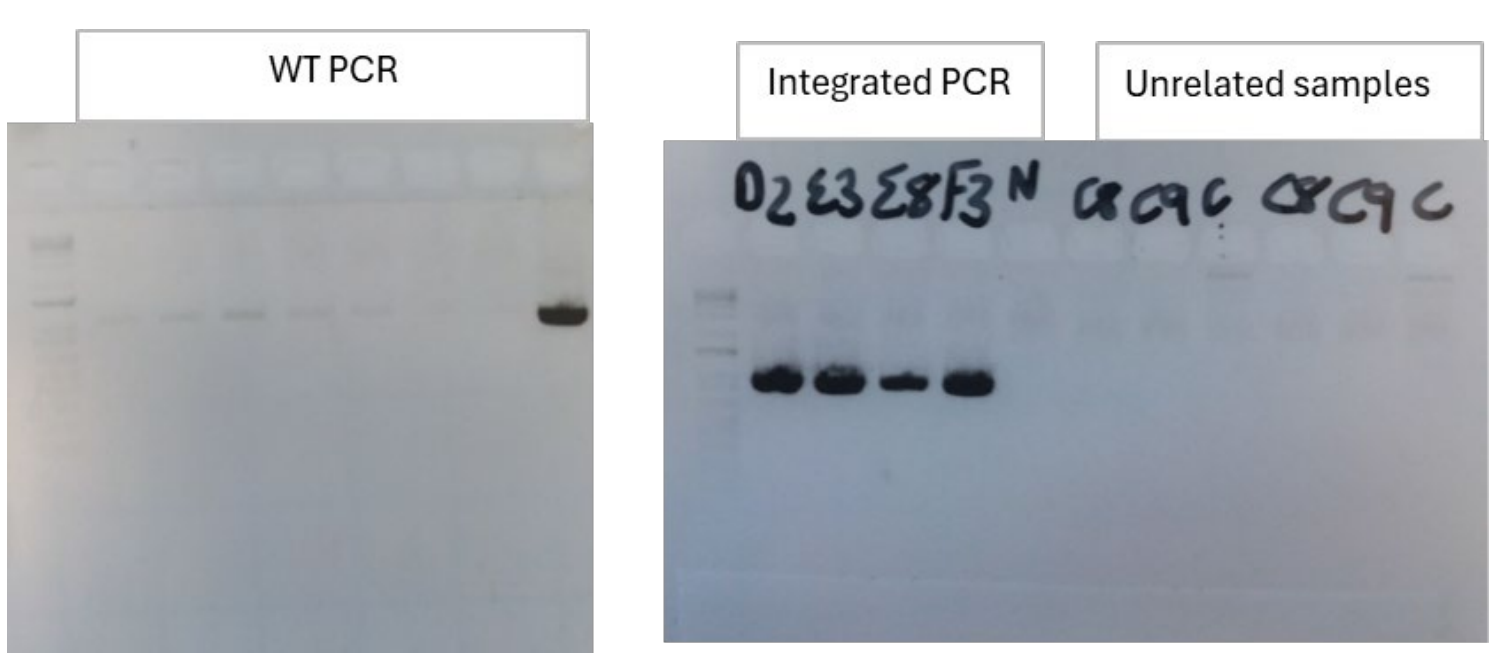

Figure S3C

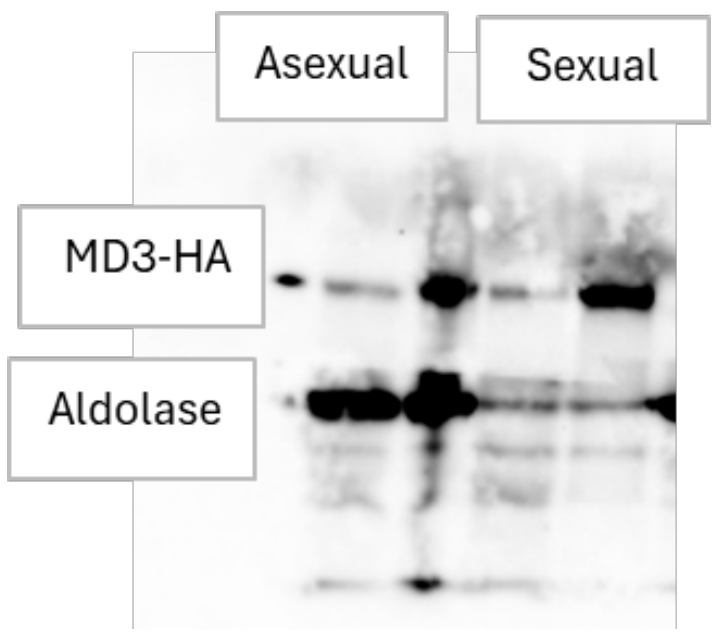

Figure S4C

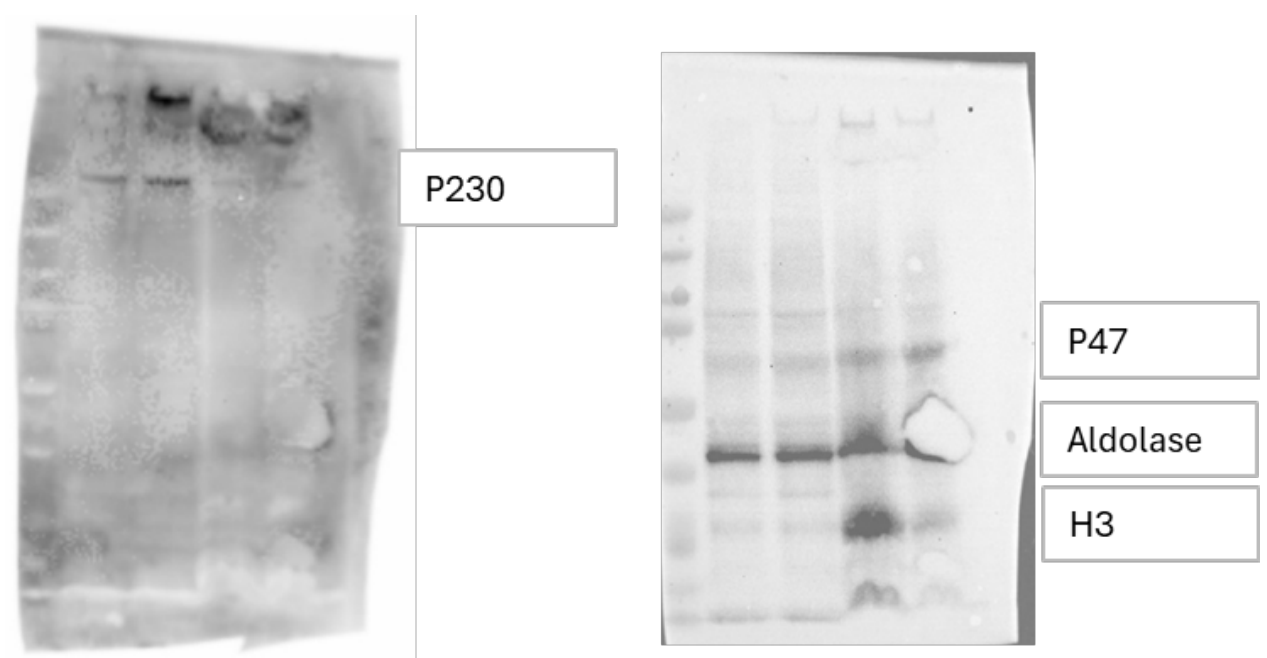

Figure S5A

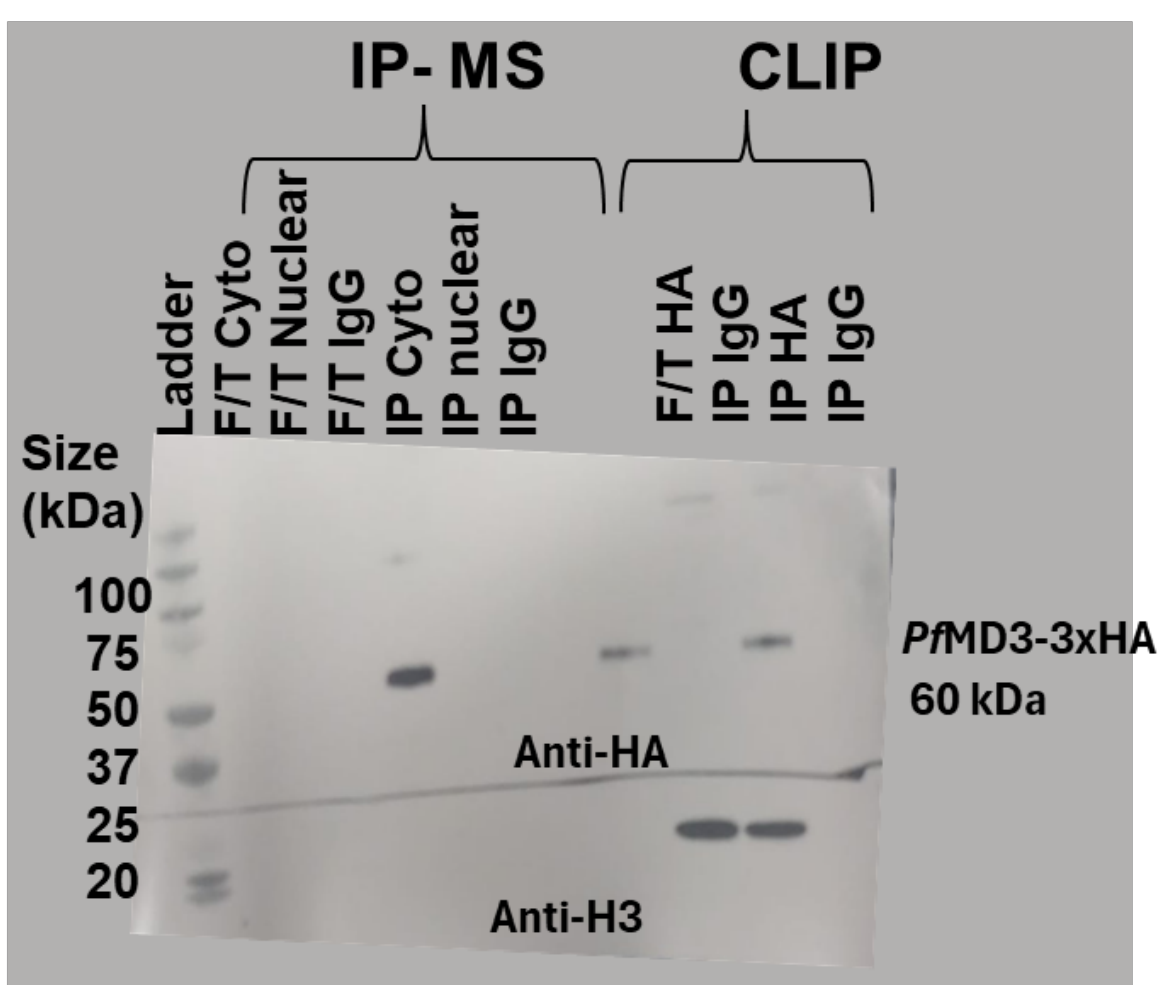

Figure S6C

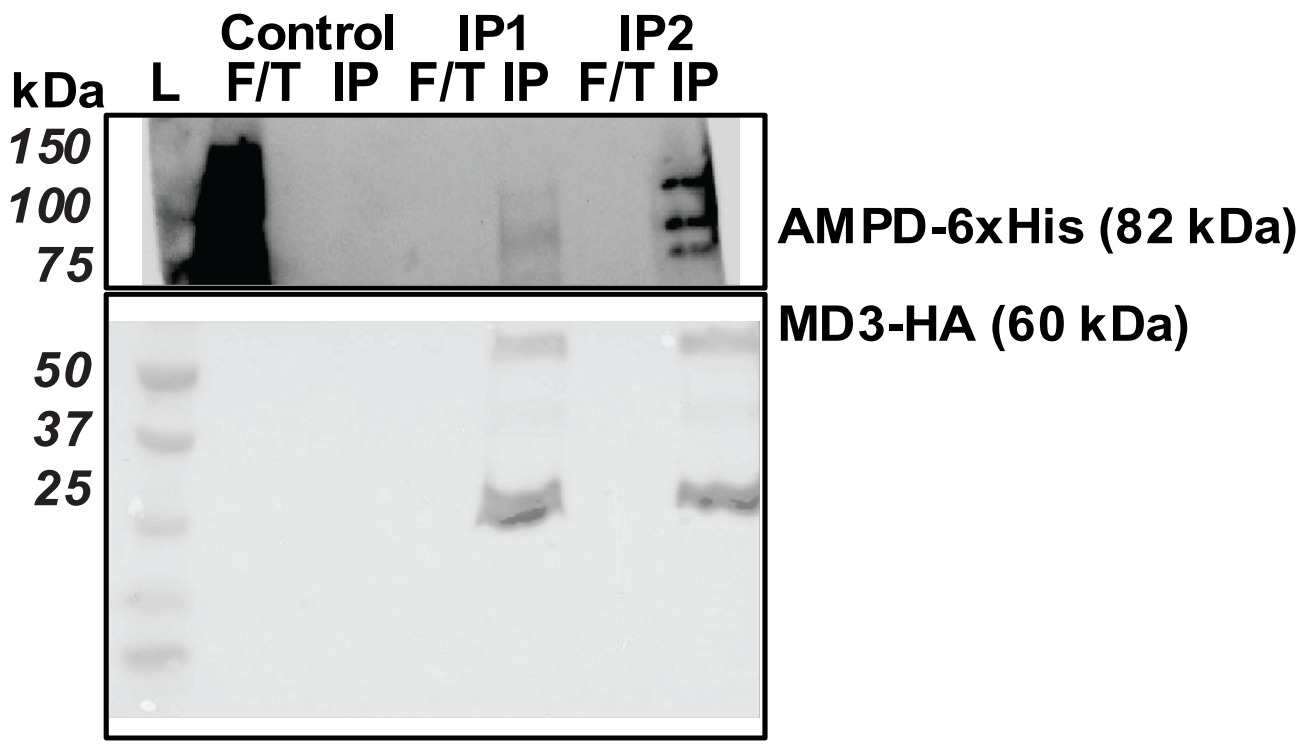

Figure S6D

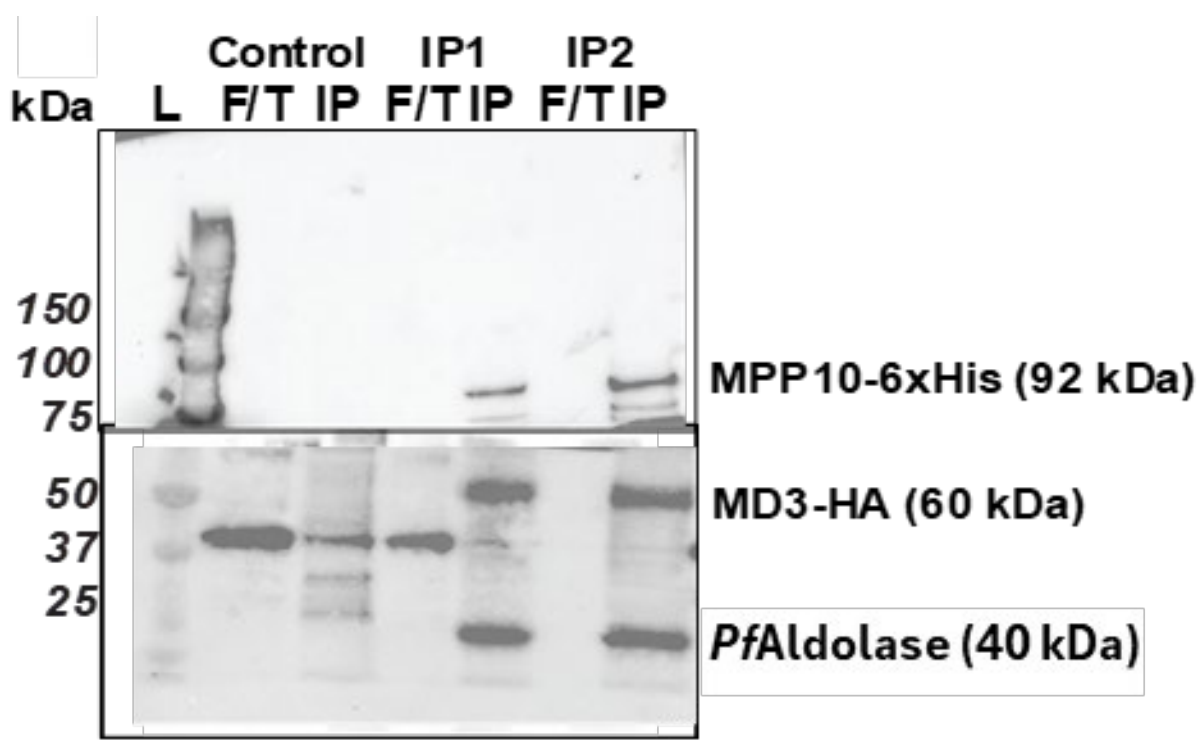

Figure S6E

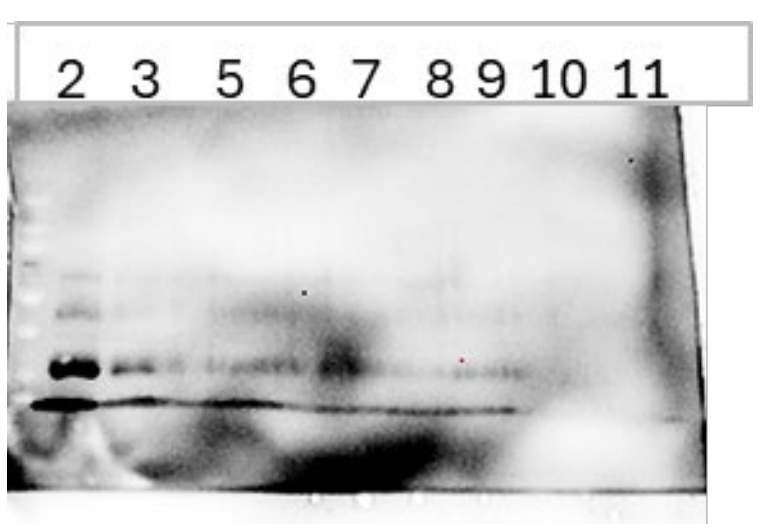

Supplement: Supplementary file 3 [file 41467_2026_77156_MOESM1_ESM.pdf]
