# Supplementary material for: The CCCH-type zinc-finger PfMD3 promotes translation for malaria parasite transmission
Source: Nat Commun. 2026 Nov 3;17:11370. doi: 10.1038/s41467-026-77156-1 (PMC13731652; doi:10.1038/s41467-026-77156-1)
Supplement: Supplementary file 4 [file 41467_2026_77156_MOESM2_ESM.pdf]

Description of Additional Supplementary Files for:

The CCCH-type zinc-finger *Pf*MD3 promotes translation for malaria parasite transmission

Title: Supplementary Data 1

Description: Data pertaining to bulk transcriptomic analysis of PfMD3 knockout parasites.

Title: Supplementary Data 2

Description: Data pertaining to proteomic analysis of PfMD3 knockout parasites.

Title: Supplementary Data 3

Description: Data pertaining to CLIP-seq analysis of PfMD3-3xHA parasites.

Title: Supplementary Data 4

Description: List of mRNA targets bound by PfMD3-3xHA identified by CLIP-seq that were significantly DE in either the transcriptomic (Table S1) or proteomic (Table S2) datasets

Title: Supplementary Data 5

Description: Data pertaining to IP-MS analysis of PfMD3-3xHA parasites

Title: Supplementary Data 6

Description: Data supporting genotyping for parasite lines used in the study, including primer and probe sequences and results of whole genome sequencing

Title: Supplementary Data 7

Description: Data pertaining to single cell RNA-seq transcriptomic analysis of PfMD3 knockout parasites
